# Spatiotemporal development of coexisting wave domains of Rho activity in the cell cortex

**DOI:** 10.1038/s41598-021-99029-x

**Published:** 2021-09-30

**Authors:** Siarhei Hladyshau, Mary Kho, Shuyi Nie, Denis Tsygankov

**Affiliations:** 1 School of Biology, Georgia Institute of Technology, Atlanta, GA USA; 2 Wallace H. Coulter Department of Biomedical Engineering, Georgia Institute of Technology and Emory University, Atlanta, GA USA

**Keywords:** Computational models, Dynamical systems

## Abstract

The Rho family GTPases are molecular switches that regulate cytoskeletal dynamics and cell movement through a complex spatiotemporal organization of their activity. In *Patiria miniata* (starfish) oocytes under in vitro experimental conditions (with overexpressed Ect2, induced expression of Δ90 cyclin B, and roscovitine treatment), such activity generates multiple co-existing regions of coherent propagation of actin waves. Here we use computational modeling to investigate the development and properties of such wave domains. The model reveals that the formation of wave domains requires a balance between the activation and inhibition in the Rho signaling motif. Intriguingly, the development of the wave domains is preceded by a stage of low-activity quasi-static patterns, which may not be readily observed in experiments. Spatiotemporal patterns of this stage and the different paths of their destabilization define the behavior of the system in the later high-activity (observable) stage. Accounting for a strong intrinsic noise allowed us to achieve good quantitative agreement between simulated dynamics in different parameter regimes of the model and different wave dynamics in *Patiria miniata* and wild type *Xenopus laevis* (frog) data. For quantitative comparison of simulated and experimental results, we developed an automated method of wave domain detection, which revealed a sharp reversal in the process of pattern formation in starfish oocytes. Overall, our findings provide an insight into spatiotemporal regulation of complex and diverse but still computationally reproducible cell-level actin dynamics.

## Introduction

Rho family small GTPases are the key regulators of cytoskeletal organization and dynamics^1,2^ and have been a subject of an extensive research effort in the biomedical community. Advanced molecular biosensors^3–5^ and optogenetic tools^6–8^ revealed a complex, coordinated spatiotemporal dynamics of Rho activity during cell motion. Although GTPases and their regulators guanine nucleotide exchange factors (GEFs) and GTPase-activating proteins (GAPs) are a part of a large and complex signaling network^9^, the emerging cell-level activity of GTPases exhibits characteristic patterns of wave propagation or static formations that have been studied mathematically in many other biological contexts^10–17^. Recently, a particular type of mathematical models, mass-conserved activator-substrate (MCAS) models, also referred to as wave-pinning models^18–21^, have gained popularity because they account for the switch-like behavior of GTPases due to their autocatalytic activity^22^ and successfully capture polarization patterns observed in experiments^20,21,23–25^. The wave-pinning model can be viewed^19^ as a subclass of MCAS models with a particular form of the non-linear dependence (Hill-function) in the reaction terms of the reaction–diffusion equations^18,26,27^. Alternatively, researchers have also used quadratic functions^19,23^ and different terminology for these models^28^. In all these models, active and inactive forms of GTPases are presented with diffusion coefficients that differ by orders of magnitude, because GTPases are considered to be membrane-bound in active form and cytosolic in inactive form (see Ref.^29^ for more details). Recently, a model that explicitly accounts for bulk-surface interactions of GTPases in an arbitrary-shaped cell was developed by Cusseddu et al*.*^26^. MCAS models include autocatalytic (positive) feedback that amplifies small perturbations and allows cell to generate a strong, persistent response to a weak local cue. Coupling of autocatalytic activation with a local inhibition (negative feedback) generates diverse dynamic regimes depending on the parameters representing the strength of feedbacks^30^. For example, Holmes et al.^27^ showed that even a one-dimensional model (1D) with three components, including active and inactive forms of a nucleation-promoting factor and the inhibition through F-actin, could generate a stable polarization patch, oscillating polarization patch, reflecting waves, a single terminating wave, wave train, and more exotic formations.

**Figure 1:**
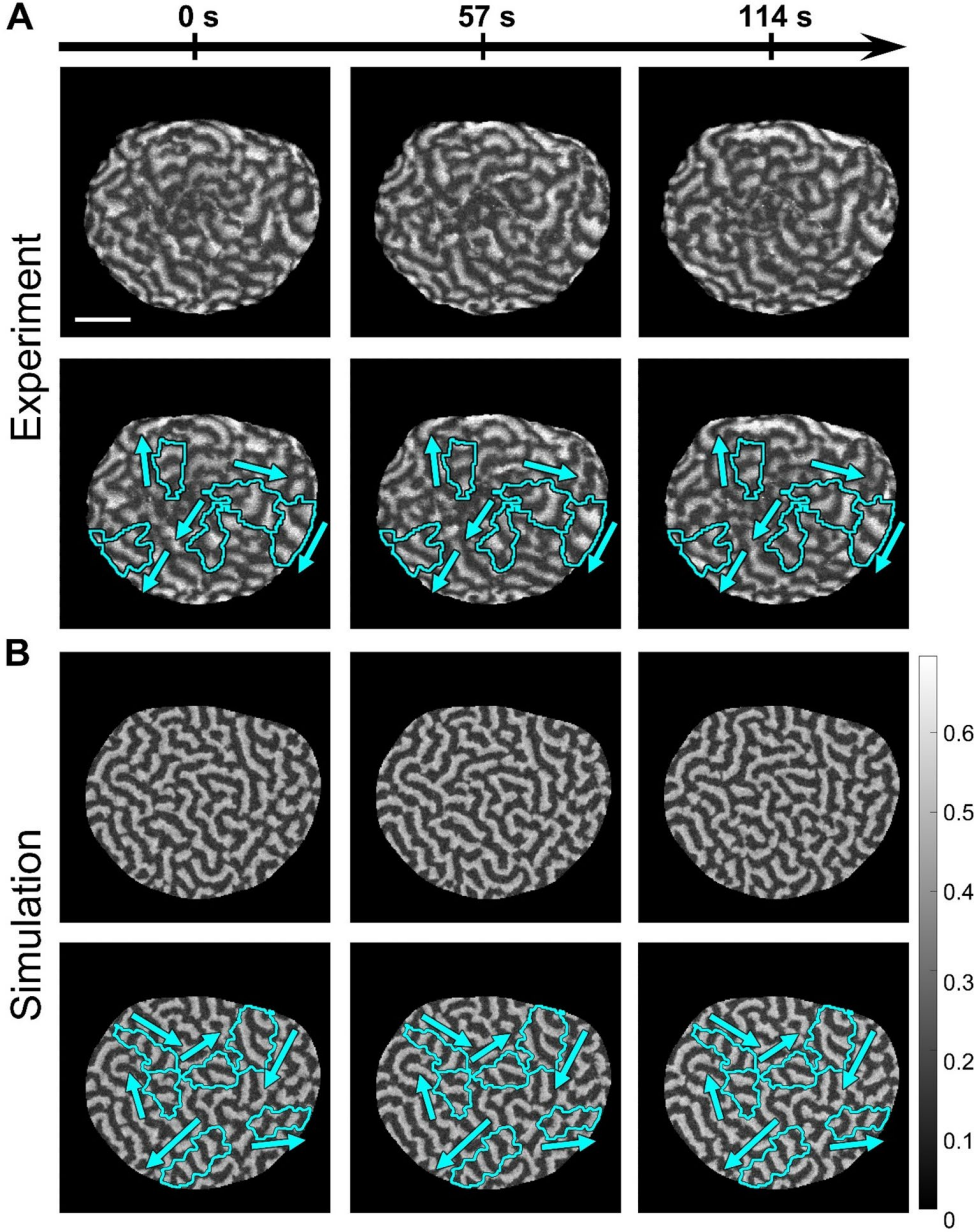
Cortical waves of Rho activity in *Patiria miniata* oocytes. Here oocytes have overexpressed Rho-GEF Ect2, induced expression of ∆90 cyclin B, and are treated with roscovitine. Both the experiment^31^ and our model show similar overall dynamics and the presence of wave domains. (**A**) Snapshots of wave propagation in the experiment. The images are generated from the data published in Bement et al*.*^31^ with the permission of the authors. The scale bar is 50 μm. (**B**) Snapshots of wave propagation in our simulation (${k}_{0}=0.2$, ${s}_{2}=1$, ${\alpha }_{1}=3$, ${\alpha }_{2}=0.1$). The cyan contours outline wave domains as defined in the following section, while the arrows indicate the direction of wave propagation in these domains. For the full dynamics see Supplementary Video 16 in Ref.^31^ and our Supplementary Video S1.

In this work, we use a two-dimensional version of the MCAS model coupled with local inhibition to investigate a particular type of wave dynamics that, to our best knowledge, was not studied in the biological context but was recently observed in *Patiria miniata* (starfish) oocytes in-vitro^31^. Shortly after the initiation of anaphase, starfish oocytes generate cortical waves of the active form of Rho-GTPase, followed by the front of F-actin polymerization. It was shown that such waves are involved in the process of cytokinetic furrow formation^25,31^. Under experimental conditions when cells overexpress Rho-GEF Ect2, the waves propagate throughout the whole cell cortex. The initiation of waves is closely associated with the progression of the cell cycle and is regulated by Cdk1 activity. The arrest of Cdk1 inactivation by ∆90 cyclin B terminates wave dynamics because Cdk1 inactivation is needed for the excitability of the cell cortex (see Ref.^31^ for details). Under these conditions, the pharmacological treatment of cells with roscovitine (that inhibits Cdk1) activates cortical waves that propagate on the whole cell scale (because of Ect2 overexpression) and are not perturbed by the formation of cytokinetic furrow. An interesting aspect of such dynamics is partitioning of the whole cell area into seemingly independent regions of wave propagation, henceforth referred to as *wave domains* (Fig. 1A). Each domain is characterized by coherent wave propagation with a small variance of the direction of wave vector. In the neighboring domains, waves propagate in different (often the opposite) directions with respect to each other. Such dynamics is very different from the formation and competition of multiple spiral waves, which is often observed in excitable media^13,16,17^. Indeed, spiral waves have wave vectors that point in all directions away from the spiral center. Our simulations show a strikingly close resemblance of the model behavior and the experimental time-lapse records (Fig. 1B and Supplementary Video S1). Visually, the dynamics is very complex because the domains do not remain static but gradually change their shape and number over time. However, our analysis reveals the underlying processes that drive the initiation and development of wave domains.

In the original paper^31^, the authors proposed a computational model of actin and Rho dynamics in cell cortex. This model was also studied in detail in the follow-up paper by Goryachev et al*.*^25^. However, the focus of this modeling effort was to investigate the formation of a static cytokinetic furrow in the presence of dynamics waves. This model was not applied to describe wave domains and the process of their development.

One of the challenges in the analysis of the spatiotemporal behavior in a biological system is that an experimental observation may correspond to a transient behavior of the model. For example, in our model, one of the competing wave domains eventually wins, taking all the available space. This happens on a time scale much larger than the cell cycle. This is a problem because formal mathematical analysis of dynamical systems typically relies on identifying steady states and performing the stability analysis in their vicinity^32,33^. In the recent work of Liu et al*.*^34^ the local perturbation analysis (LPA) was applied to investigate the bifurcation diagram of the 1D wave-pinning model from Holmes et al*.*^27^. Given the complicated structure of the diagram, the authors acknowledged that oscillations predicted by Hopf bifurcations should be interpreted with caution, because these oscillations can be damped by diffusion. Thus, the resulting dynamics of the system cannot be inferred directly from the diagram. The authors recognized that explanation and interpretation of the LPA analysis remain an open problem. Previously, other authors^35^ applied Lyapunov Exponent Theory to predict Turing diffusion-driven instability. However, this approach does not allow to predict the properties of the pattern during the phase of exponential amplitude growth, which is an important part of our study of the wave domain formation. Thus, to study the details and the scope of possible transient behaviors (far from a steady state), we still have to rely on a systematic analysis of the parameter space using simulations.

In this work, we first characterize the parameter regimes responsible for different types of dynamic behavior. We show that oscillatory behavior and instability of the homogeneous steady state is not enough for the formation of wave domains and additional restrictions on the activation rate and the strength of negative feedback are required. Next, we show that the observed dynamic regimes are preceded by the development of distinct low-intensity patterns at an earlier stage of the formation process. Finally, we show that the way the system transitions from low-intensity patterns to high-intensity dynamics defines the resulting types of spatiotemporal Rho activity.

Furthermore, we explored the effects of strong intrinsic noise in the system and showed that in addition to starfish dynamics, our simulations also *quantitatively* agree with previously published^31^ and our own data on F-actin dynamics in *Xenopus laevis* (frog) embryos. However, this dynamic behavior is drastically different from the starfish oocytes. Our analysis showed that frog dynamics corresponds to a different regime in parameter space when wave domains cannot be developed.

An important part of this work is a methodology that we developed for the automated identification of wave domains in time-lapsed image records. We used this method for quantitative characterization of wave domains and comparison of our simulation with experimental examples of Rho activity in starfish oocytes. Our analysis revealed a time point when Rho dynamics *abruptly* changes on the cell level. At this moment, the process of wave domain development reverses to a gradual breakup ending with the loss of wave propagation.

## Results

### Overview of the model behaviors and the definition of wave domains

**Figure 2:**
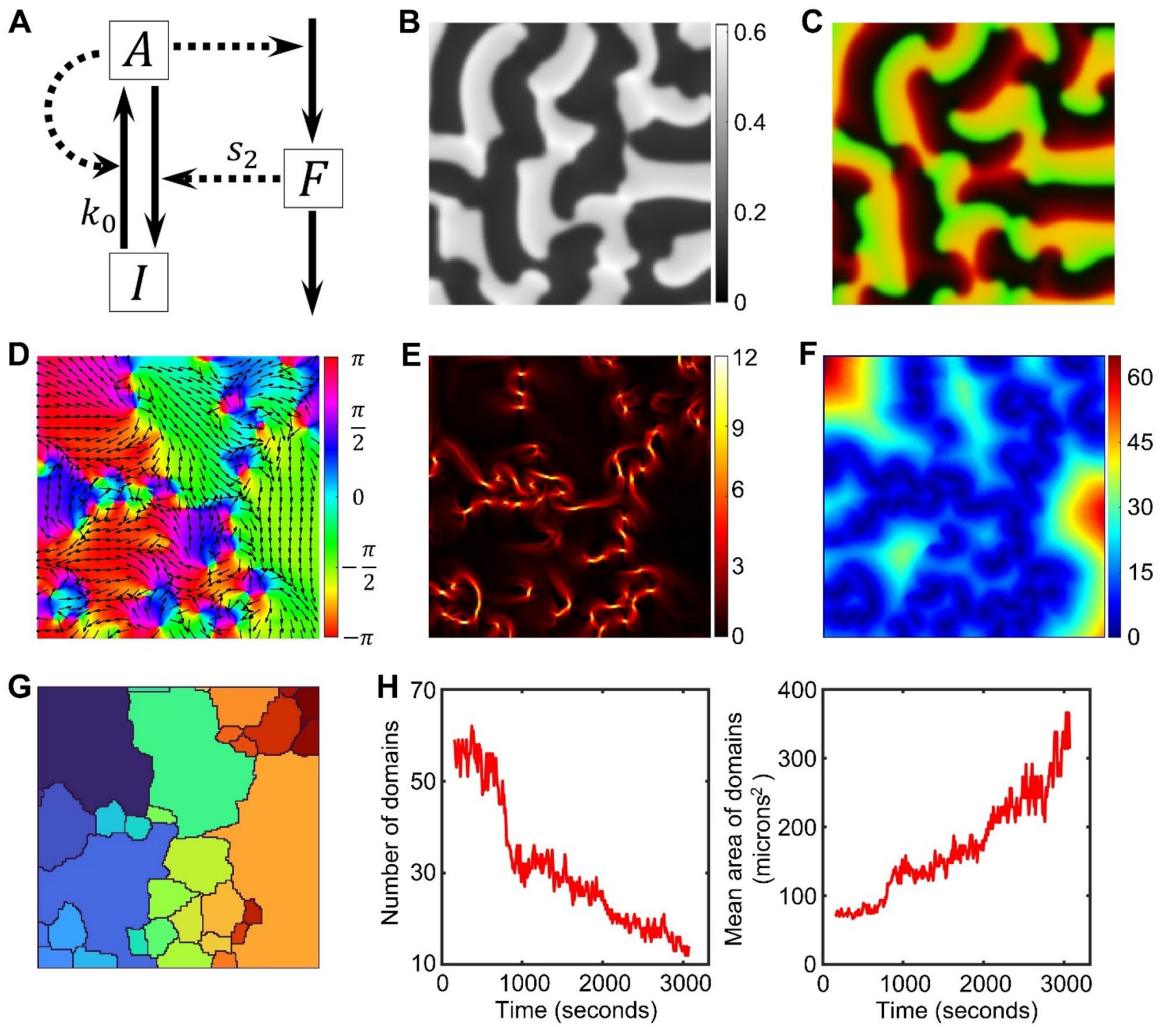
The model and an illustration of the wave domain concept. (**A**) Diagram of the signaling motif. $A$ and $I$ represent active and inactive forms of the signaling molecule, while $F$ represents a local inhibitor (see “Methods” for equations and Table 1 for parameter values). (**B**) The resulting distribution of component $A$ in a snapshot of the simulation with ${k}_{0}=0.15$, ${s}_{2}=0.8$ using homogeneous initial conditions. (**C**) An image of two merged channels: green channel represents activator $A$ and red channel represents inhibitor $F$. The concentrations were scaled as $\left[C-min(C)\right]/\left[\mathit{max}\left(C\right)-min\left(C\right)\right]$. (**D**) The colormap of the direction angles, $\theta$, of the wave vectors at the same time point as B and C. Arrows represent the direction of wave vectors. (**E**) The colormap of the magnitude of a local change (gradient) in the directions of the wave vectors. Regions of high gradient are located at the edges of wave domains. (**F**) The colormap of our metric of the coherence distance. (**G**) Segmentation of the coherence distance with the modified watershed algorithm and after merging domains with similar values of the mean $\theta$. (**H**) The dynamics of wave domains. The number of regions detected by the automated segmentation pipeline is decreasing (left panel), while the mean area of regions is increasing over time (right panel).

Some of the dynamic regimes of our two-dimensional model (Fig. 2A and “Methods”) are analogous to the one-dimensional version studied by Holmes et al*.*^27^. However, the increase of dimensionality can also lead to the emergence of novel dynamic behaviors (Supplementary Figure S1 and Supplementary Video S2).

In 1D model with weak negative feedback, the initial local spike of activity develops into a polarized state, which is a characteristic property of the MCAS systems. In the presence of strong inhibition, the model shows rich dynamics^27,30^. In the so-called *excitable regime*, the system returns to the spatially homogeneous state if the initial local stimulus is weak. With a strong enough local stimulus, the model generates single terminating or reflecting traveling waves. Alternatively, in the *oscillatory regime*, in the presence of the limit cycle and the unstable homogeneous state^27^, small perturbations are sufficient to generate wave trains or more exotic patterns. In the 2D case, the formation of a near-boundary patch naturally corresponds to the polarized state in 1D. However, in the excitable and oscillatory regimes, the 2D model generates spiral waves, which cannot be observed in the 1D setup, but is a well-characterized feature of excitable systems previously studied with other models^36–38^.

Intriguingly, in addition to the well-known spiral waves, in a particular region of parameter space of the 2D model, the system generates a more complex dynamic behavior (Fig. 1A,B), which closely resembles dynamics of the active form of Rho GTPase and F-actin in the cortex of starfish oocytes in-vitro^31^. In this parameter regime, the system self-organizes from the homogeneous unstable state into multiple coexisting regions with persistent directions of the wave propagation that are different among the regions. We refer to these formations as *wave domains* and define them as sub-regions of the cell area with.

1. low variance of the wave vector direction,
2. size larger than the wavelength of the activity waves propagating inside, and
3. time scale of their boundary movement larger than the time scale of the wave propagation.

Figure 2B provides an example of such dynamics. Similar to spiral waves, the fronts of high concentration of the activator $A$ (green channel in Fig. 2C) are followed by an increased concentration of the inhibitor $F$ (red channel in Fig. 2C) and then, by refractory regions (dark regions with low concentration of both activator and inhibitor in Fig. 2B,C). However, visual representation of the directions of the wave vector (Fig. 2D and Supplementary Figure S2) clearly shows domains of unidirectional propagation, which is different from the surrounding areas. Typically, the shapes of the wave domains are elongated in the direction of the wave propagation inside the domain. The domains can be outlined along the regions of steep gradient of the direction of wave vectors (Fig. 2E). Previously, regions with high variance of wave directions (phase-defects) were reported by La Porta et al*.*^39^ for ethanol–water mixtures in the presence of temperature gradient. Also, regions of locally coherent waves were observed in vitro in the bacterial Min system^40^, although the overall system behavior is different from the actin waves in starfish oocytes. Recently, a computational model of Rho signaling in starfish oocytes was proposed by Tan et al*.*^41^ based on complex Ginzburg–Landau equation^42^. In this study, the authors investigated the properties of topological defects. Similar to the modeling effort from Bement et al*.*^31^ and Goryachev et al*.*^25^, the authors did not aim to investigate the process of formation and development of wave domains, although visually it seems that the complex Ginsburg–Landau equation can reproduce the dynamics with multiple regions of coherent wave propagation.

### Automated identification of wave domains in time-lapse images

To investigate the properties of wave domains and their formation process in an unbiased way, we developed a method for automatic identification of the regions of coherent wave propagation. We used the time-averaged values of the directions of wave vectors (see “Methods” “Characterization of the spatial distribution of the direction of wave vectors.”) to suppress the regions with minor temporal fluctuations and to highlight the regions where wave propagation is persistent over a significant period of time. The shapes of such regions change over a longer time than the oscillation period of the activator dynamics. Based on the wave vector angles, $\theta$, we define a metric of the coherence distance for each pixel as the maximal radius of the circle, within which the circular standard deviation^43^ is smaller than a predefined critical value (${C}_{cr}=0.4$, Fig. 2F and Supplementary Figure S3A,B). Next, the metric is processed with our customized watershed algorithm. This algorithm is different from the MATLAB’s watershed^44^ in that flat regions of the input image are automatically merged with the regions that grow during the watershed process (the algorithm is available as a separate function with all other processing scripts at [https://github.com/tsygankov-lab/WaveDomains]). This modification reduces over-segmentation. The result of the watershed segmentation is further post-processed to merge the regions with the difference of mean ${\theta }_{m}$ values smaller than 0.5 radians and the length of their interface (${l}_{cr}$) larger than 10% of the square root of the domain area (Fig. 2G and Supplementary Figure S3C–E).

This method allowed us to perform the quantitative analysis of wave domains by computing their number and size as functions of time. In case when the initial homogeneous state is unstable, the system starts forming a large number of small regions, which gradually decreased in number and increase in size, thus generating wave domains over the course of the simulation (Fig. 2H).

### A balance of the basal activation rate and the negative feedback defines a distinct wave domain regime of the parameter space

**Figure 3:**
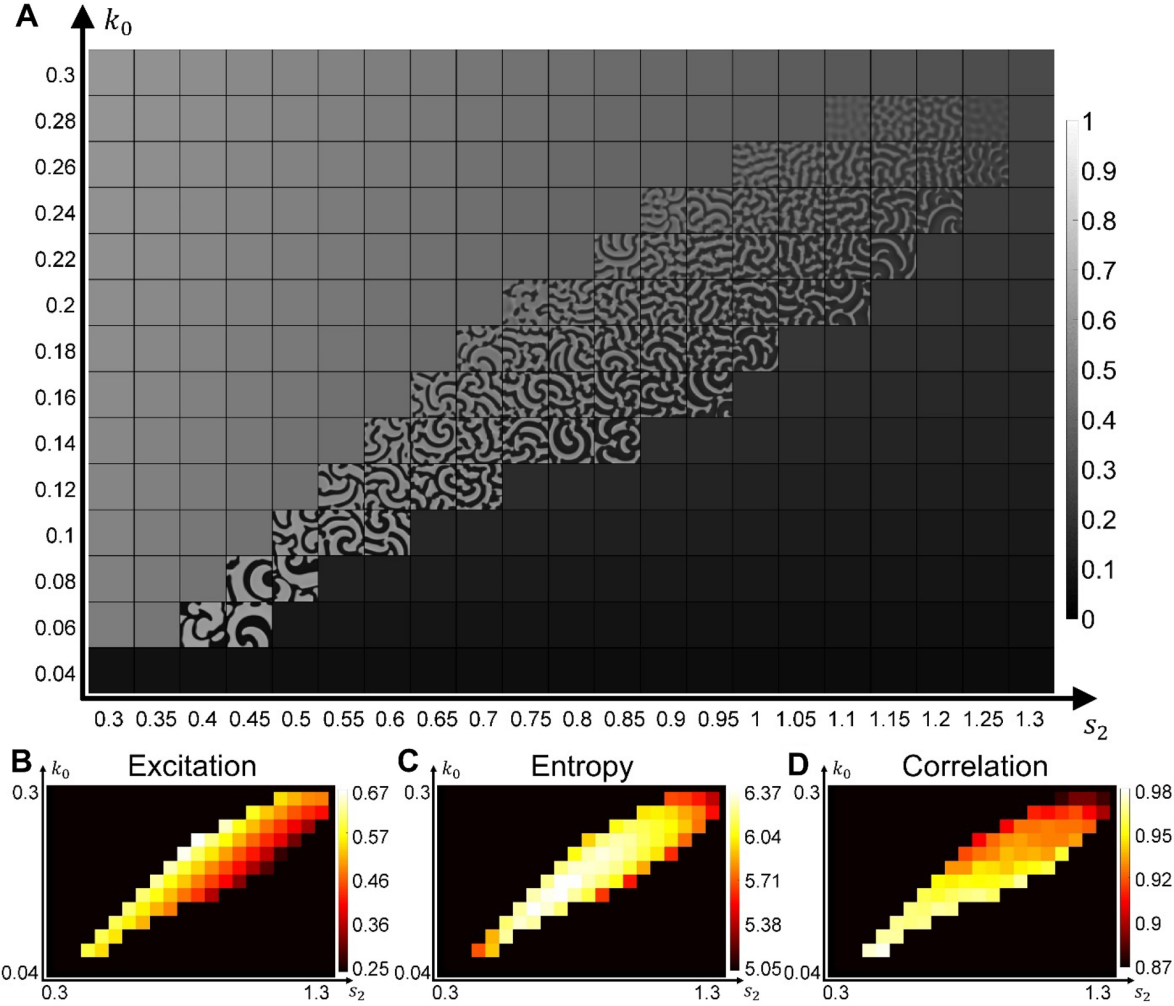
Parameter scan and textural analysis of the emerging patterns. (**A**) The result of model simulations for a range of parameters ${k}_{0}$ and ${s}_{2}$ (see also Supplementary Video S3). (**B**) The colormap of the excitation measure (see “Methods”) for the parameter space in (**A**). (**C**,** D**) The textural measures of pattern entropy and correlation for the parameter space in (**A**). Each measure was averaged over 100 simulation steps after the formation of waves from the initial homogeneous state.

Next, we investigated how the formation of wave domains is regulated by the basal Rho activation rate (parameter ${k}_{0}$, which together with the parameter $\gamma$ characterizing the strength of autocatalytic activation contributes to the total rate of Rho activation) and by the inhibition through F-actin (parameter ${s}_{2}$ in the negative feedback term, which together with the basal deactivation rate ${s}_{1}$ contributes to the total rate of Rho deactivation). The choice of these two parameters for the analysis is motivated by previous studies of this model in 1D^27^ that have shown diverse regimes of the system’s dynamics. Figure 3A shows the parameter-space scan, with the snapshots of the spatiotemporal patterns in the regime of wave dynamics, while the excitable regime remains inactive for the homogeneous initial conditions with the minimal noise, ${\alpha }_{\text{1,2}}={10}^{-15}$ (see Supplementary Video S3). The increased inhibition is associated with the increase in the size of the refractory region (Fig. 3A) and the decrease of the total level of Rho activity (Fig. 3B). Both increasing and decreasing inhibition (the left and right sides of the regime of wave dynamics) lead to the formation of large spirals and a decrease in the image entropy (Fig. 3C, Supplementary Video S3, and “Methods”). The entropy is highest in the middle region of the parameter space, where we see the formation of wave domains. Finally, in the lower-left region, where both activation and inhibition are low, waves have large activation fronts, which prevents the formation of wave domains and leads to patterns with a high image correlation (Fig. 3D).

Thus, wave domains are formed only for a subset of parameter values, corresponding to the regime of wave dynamics where the proper balance between the basal rate of activation and the strength of negative feedback is maintained.

### The spontaneous formation of wave domains proceeds in two distinct stages of dynamic behavior

**Figure 4:**
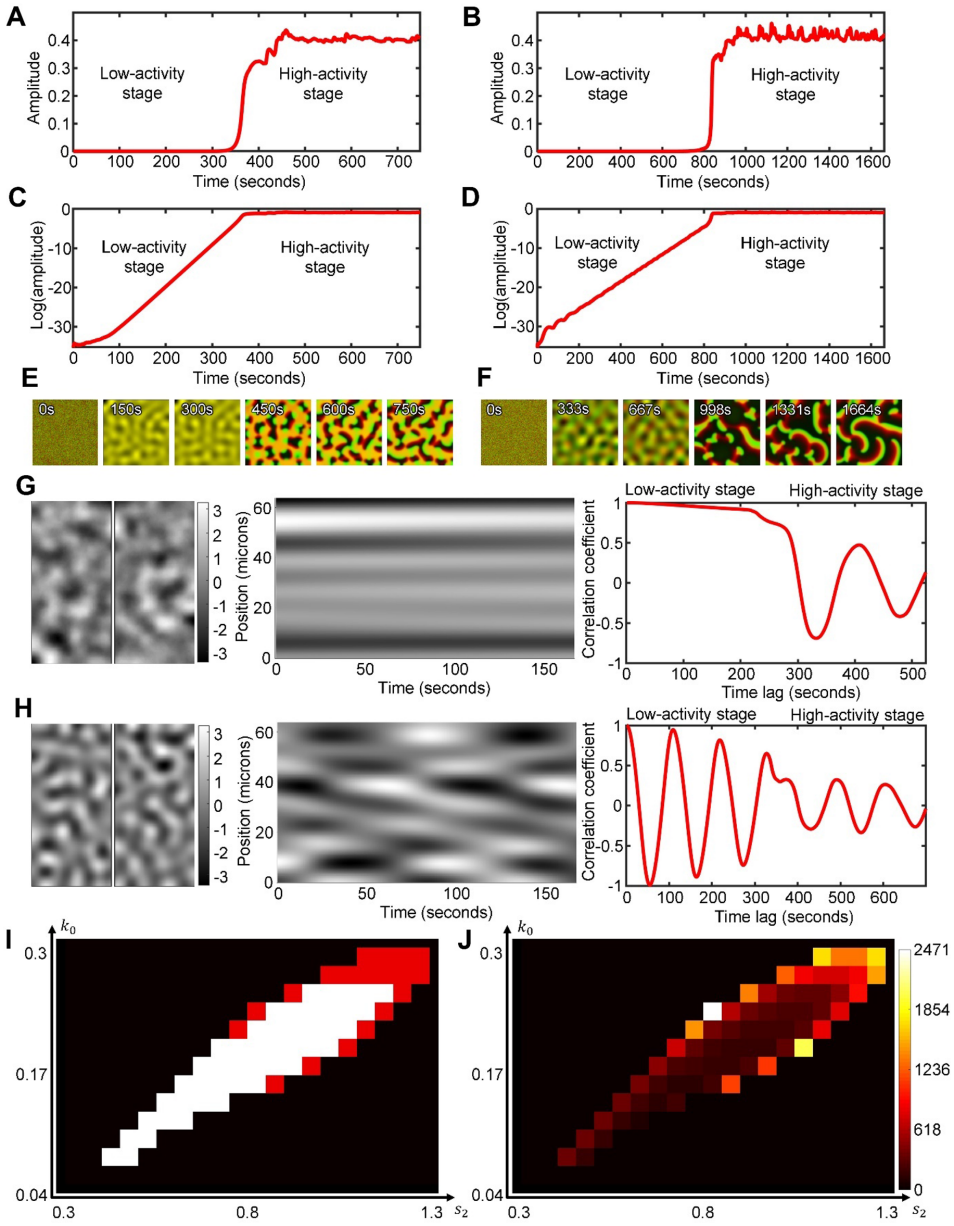
Two stages of pattern development and characteristics of the regimes in the low-activity stage. (**A**,**B**) The amplitude of the activator pattern, $\underset{{x},{y}}{\text{max}}A-\underset{{x},{y}}{\text{min}}A$, during the low- and high-activity stages for the quasi-static low-activity regime (${k}_{0}=0.2$*,* ${s}_{2}=0.8$) and the oscillatory low-activity regime (${k}_{0}=0.2$*,* ${s}_{2}=1.1$), respectively. (**C**,**D**) The same plots as in (**A**), (**B**), but in the log scale. (**E**,**F**) A series of snapshots of the system behavior from the same parameters as in (**A**,**C**) and (**B**,**D**), respectively (see also Supplementary Videos S4–S6). The concentrations were scaled as $\left[C-\text{min}C\right]/\left[\text{max}C-\text{min}C\right]$ both for the inhibitor (red channel) and the activator (green channel) before the channels were merged together. (**G**,**H**) (Left panels) Snapshots of the system in the quasi-static (${k}_{0}=0.2$, ${s}_{2}=0.8$) and oscillatory (${k}_{0}=0.2$, ${s}_{2}=1.1$) regimes of the low-activity stage; top and bottom panels respectively. (Central panels) Kymographs of the system dynamics along the vertical lines shown in the left panels. For comparison of patterns with different amplitude, the concentration values were scaled to zero mean and standard deviation equal to one (mean value was subtracted from each pixel and then divided by standard deviation). (Right panels) Temporal autocorrelation plots, showing the correlation coefficient as a function of the time lag (see “Methods”). (**I**) Classification of the parameter space with white and red colors indicating the quasi-static and oscillatory regimes, respectively. (**J**) Colormap showing the duration of the low-activity stage (in s).

To better understand the process of wave domain formation, we looked closer at how the system behavior changes over time (Fig. 4A,B). In our simulations, small perturbations in the homogeneous state start to grow persistently (low-activity stage) until the difference between the maximal and minimal concentrations of the activator reaches and stays at a nearly constant level (high-activity stage). The distinction between the stages becomes clearly visible when the activity level is plotted in log scale (Fig. 4C,D). To be precise, we define the stage of low-activity as a period when the slope of the pattern amplitude in log scale, $\frac{d}{\mathit{dt}}\text{log}\left(\underset{x,y}{\text{max}}A-\underset{x,y}{\text{min}}A\right)$ is larger than $\frac{{10}^{-3}}{a.u. \; of \; time}$. The stage of high-activity is defined as a period after the low-activity stage when the slope of the pattern amplitude in log scale is smaller than $\frac{{10}^{-3}}{a.u. \; of \; time}$. The value of the pattern amplitude, $\underset{x,y}{\text{max}}A-\underset{x,y}{\text{min}}A$, right before the system transitions from the low-activity stage to the high-activity stage (i.e., at time when the slope of the pattern amplitude in log scale crosses the threshold $\frac{{10}^{-3}}{au \; of \; time}$), is visualized for different model parameters in Supplemental Figure S4A.

Although the activity in the earlier stage grows exponentially with time, its spatial distribution has a specific pattern with a well-defined periodic structure (see time points 150 s and 300 s in Fig. 4E and time points 333 s and 667 s in Fig. 4F). In order to see the activity at this low level, the images are scaled individually between the minimal and maximal concentration values of the current time point. Depending on parameters, such patterns in the low-activity stage can be quasi-static (Fig. 4G) or oscillatory (Fig. 4H). In the quasi-static case, special patterns do not change significantly over time, while in the oscillatory case, spatial patterns change cyclically with a regular period. The quasi-static low-activity stage develops at intermediate values of the negative feedback (white region in Fig. 4I and the central part of the parameter space in Fig. 3C), when the pattern entropy is high. In this regime, both activator and inhibitor are co-localized and have identical spatial distributions (Supplementary Video S4, S5). In contrast, the oscillatory low-activity stage is associated with high or low negative feedback and high values of ${k}_{0}$ (red regions in Fig. 4I and the low entropy part of the oscillatory regime in Fig. 3C). In this regime, the activator and inhibitor periodically switch spatial peaks moving out of phase (Supplemental Video S6). The quasi-static and oscillatory regimes are also associated with different durations of the low-activity stage. We measured the duration of the low-activity stage as the period of linear growth of the pattern amplitude in log scale before the constant value of the amplitude is reached (Fig. 4C,D). Supplementary Figure S5 shows the slope of the amplitude growth in log scale for simulations with different parameters and at different times of the process. The slope is highest in the central part of the parameter space where the system reaches the high-activity stage faster than in the regimes that are closer to the periphery of the region of wave dynamics. Thus, the duration of the low-activity stage is small when the strength of negative feedback (parameter ${s}_{2}$) has intermediate values but rapidly increases at higher and lower values of ${s}_{2}$ (Fig. 4J). In contrast to the low-activity stage, the high-activity stage is always dynamic. At this stage, the inhibitor and activator concentrations do not overlap in space, and the time delay between their peaks drives the wave propagation.

### The directions of the wave vectors are defined during the transition from low- to high-activity stage

The regimes of the low-activity stage generate different mechanisms of transition to the high-activity stage. In the first case, the system switches the behavior from quasi-static to dynamic (Fig. 4G). In the second case, one type of dynamic behavior turns into a different one (Fig. 4H). Here we investigate the details of this process. Specifically, we ask what defines the directionality of wave propagation in both cases.

**Figure 5:**
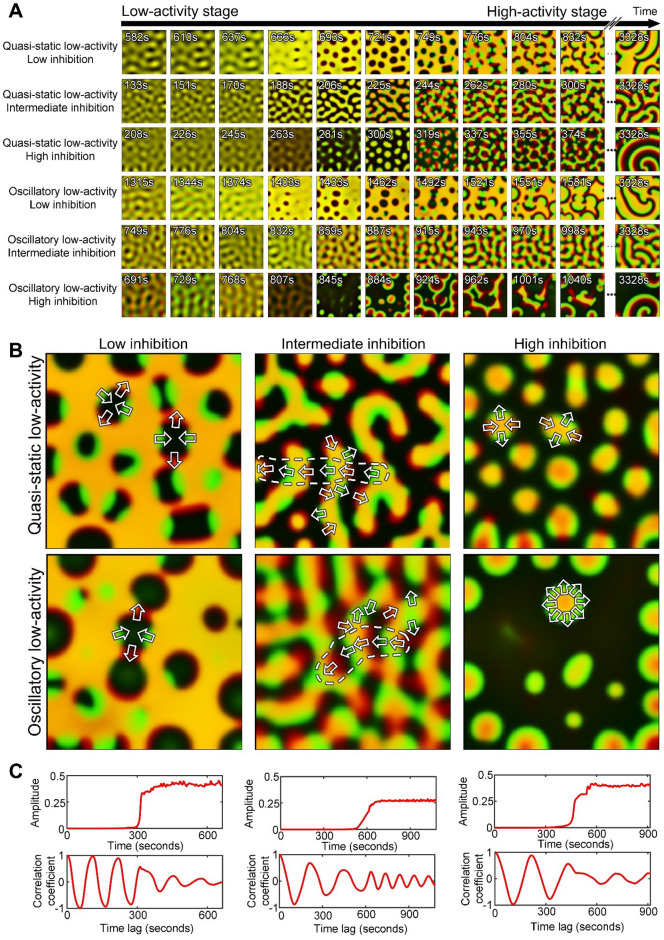
The transition of the system between the low- and high-activity stages. (**A**) Snapshots of the patterns at low- and high- activity stages for different values of the inhibition strength: (1) weak (${k}_{0}=0.18$, ${s}_{2}=0.7$ for quasi-static and ${k}_{0}=0.2$, ${s}_{2}=0.75$ for oscillatory regimes), (2) balanced (${k}_{0}=0.2$, ${s}_{2}=0.9$ for quasi-static and ${k}_{0}=0.26$, ${s}_{2}=1.05$ for oscillatory regimes), and (3) strong (${k}_{0}=0.2$ 2, ${s}_{2}=1.1$ for quasi-static and ${k}_{0}=0.2$, ${s}_{2}=1.1$ for oscillatory regimes). (**B**) Snapshots of the system at the beginning of the high-activity stage. Arrows indicate the direction of wave propagation. Dashed outlines indicate vector alignment on a larger scale. Images in (**A**), (**B**) show two merged channels: green for the activator and red for the inhibitor. (**C**) The activity amplitude and temporal autocorrelation for the oscillatory low-activity stage with weak negative feedback (left), balanced activation and inhibition (center), and strong negative feedback (right).

The quasi-static low-activity stage forms periodic patterns that stay spatially fixed while the amplitude of the activity grows. However, during the transition to the high-activity stage, the shape of patterns changes. For low inhibition values (parameter ${s}_{2}$) the system forms *reversed spots*^45^. For intermediate values of inhibition, the system develops *labyrinth-like structures*. Finally, for high values of inhibition, we observe *localized spots* (Fig. 5A). These patterns closely resemble the well-known Turing patterns observed, for example, in the Gray-Scott model^16,46^. Such patterns appear when both activator and inhibitor are co-localized. In Fig. 5A, yellow color is produced by merging two channels: green for the activator and red for the inhibitor. Wave propagation is triggered when the activator and inhibitor are spatially displaced relative to each other (Fig. 5B). The displacement occurs along the perimeter of the patterns in a periodic manner with the activation patch (green) followed by the inhibitor patch (red). This periodicity leads to the formation of small regions of coherent wave vectors with alternating directions along the Turing-like patterns (Fig. 5B). For a longer perimeter of the labyrinth patterns, we see more alignments of the wave vectors. This alignment explains why wave domains are observed at an intermediate inhibition strength (Fig. 3A,C) when more complex patterns have high entropy. In contrast, for high and low values of the parameter ${s}_{2}$, spots and reversed sports initiate waves propagating in radial directions toward and outward the centers so that there is no alignment on the larger scale. (Fig. 5B).

Interestingly, during the transition from the oscillatory low-activity stage, the system with weak negative feedback also forms reversed spots but only transiently (Fig. 5A). If the negative feedback is strong, the system forms circular waves with the wave vector pointing in all directions (Fig. 5B). In both cases, no wave domains are observed. However, when activation and inhibition are balanced, the transition between low- and high-activity stages continues smoothly without the formation of any distinct transient patterns (Fig. 5A). Consequently, the system with unbalanced activation and inhibition shows a phase-shift in the oscillation upon the transition (left and right panels in Fig. 5C), while no phase-shift is observed otherwise (central panels in Fig. 5C).

**Figure 6:**
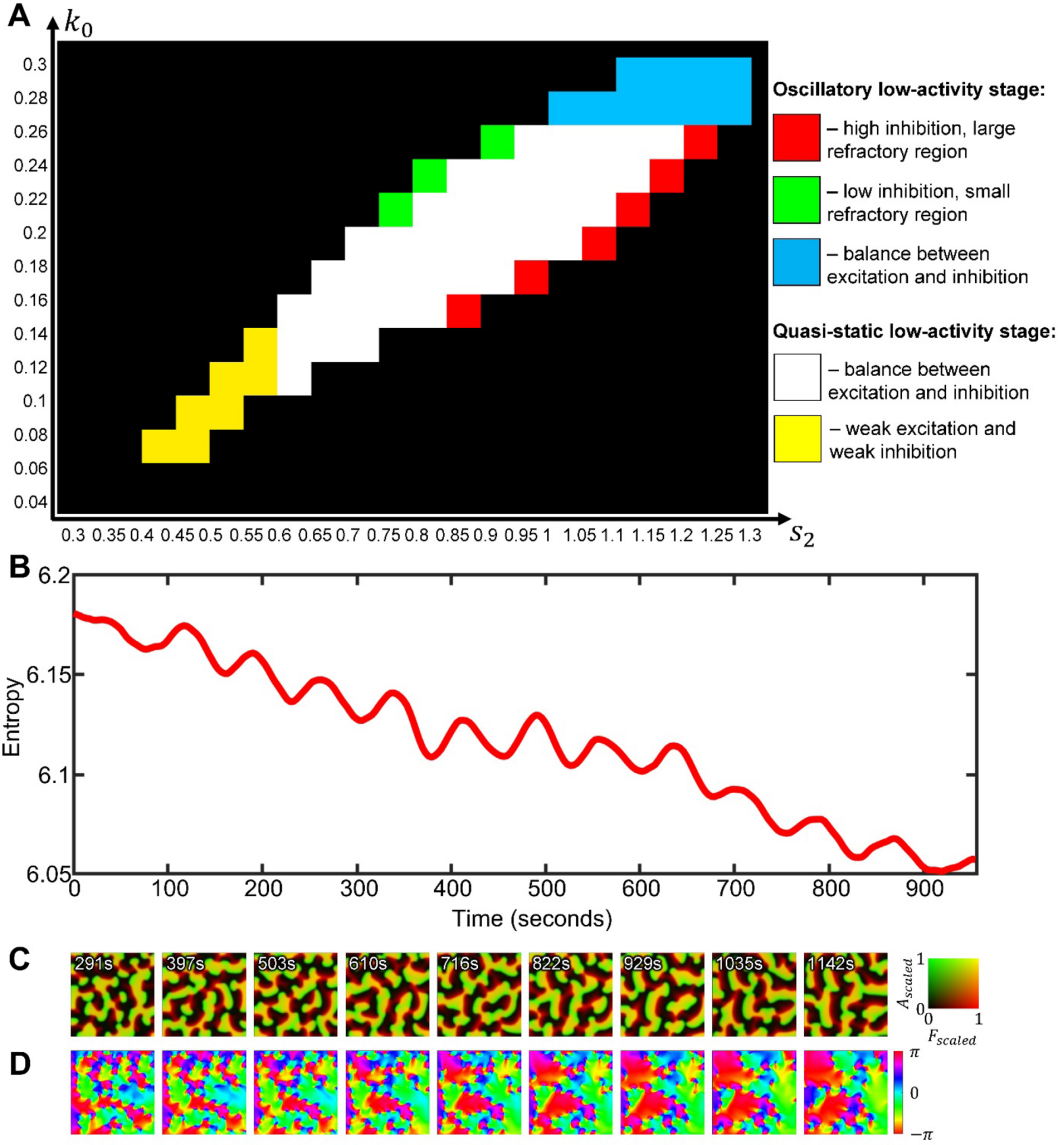
Classification and long-term dynamics of wave domains. (**A**) Different types of system’s behaviors in the space of parameters representing the basal activation rate and the strength of negative feedback. (**B**) Textural entropy as a function of time during the growth of wave domains (${k}_{0}=0.15$, ${s}_{2}=0.8$). (**C**) Snapshots from the simulation in B with the simultaneous visualization of the inhibitor and activator in the system (merged channels). (**D**) Colormaps of the direction of wave propagation in (**C**) showing the increasing size and decreasing number of the wave domains over time. The values of wave vector angles were averaged with a running window of 83 s.

Figure 6A shows regime classification based on the properties of the two stages of pattern development. When the activation and inhibition are balanced, the total levels of the active and inactive forms of the signaling molecule in the cell are similar to each other so that the activation front and the refractory region are also close in size. This is a situation when patterns of low-activity stage are quasi-static, while in the high-activity stage, intermediate values of inhibition lead to the formation of wave domains (the white region in Fig. 6A). When both activation (${k}_{0}$) and inhibition (${s}_{2}$) are weak (the yellow region in Fig. 6A), the system generates large spiral waves with a prolonged front of activation. In the opposite regime, when both activation and inhibition are strong (blue region in Fig. 6A), the system operates in the oscillatory regime during the low-activity stage and forms wave domains in the high-activity stage. This regime also has equivalent total levels of the active and inactive form of the molecule. In the border-line regions of the parameter space (green and red regions of Fig. 6A), the system forms spiral waves with a larger activation front or a larger refractory region, respectively. Therefore, the balance of activation and inhibition is the main requirement for the formation of wave domains.

So far, we considered pattern development from an initial state with a perturbation by a very small noise. However, strong persistent noise in the system may have a significant effect of its spatiotemporal behavior. To explore the system’s response to noise, we repeated the parameter scan shown in Fig. 3A but with large values for ${\alpha }_{1}$ and ${\alpha }_{2}$ in Eqs. (1–3) (Supplementary Figures S6, S7 and Supplementary Video S7). One effect of the noise is that high-activity patterns develop in a larger area of the parameters space because strong perturbations push the system away from the homogeneous state, which would otherwise remain stable. Another, more interesting, effect is the disruption of the wave domain formation in the parameter regime with oscillatory low-activity stage and the balance between excitation and inhibition (cyan region in Fig. 6A). With high noise, this parameter regime generates random patches of activity with little or no lateral movement. Such flickering closely resembles Rho and F-actin dynamics in frog oocytes^31^ and frog blastomeres in our experiments (see “Methods”). In contrast, the parameter regime with quasi-static low-activity stage and the balance between excitation and inhibition (white region in Fig. 6A), where the amplitude of the pattern is larger (see Supplementary Figure S4A), remains more robust to noise and still generates wave domains that closely resemble Rho dynamics in starfish oocytes (Supplementary Figure S7). Supplementary Figures S8–S10 illustrate a good quantitative agreement between our simulations in these two parameter regimes of the model and the cortical dynamics in starfish oocytes and frog blastomeres under different experimental conditions (see also Supplementary Videos S8 and S9). The detailed description of our quantitative analysis of the experimental and simulated data is provided in the Supplementary Text.

### The size and number of wave domains gradually change during the high-activity stage of the pattern development

It is important to note that once formed, wave domains continue to grow and compete with each other so that the number of domains decreases with time, which is characterized by a gradual decrease in the image entropy (Fig. 6B). The shape of the domains also changes. At the beginning of the high-activity stage, they have shapes elongated in the direction of the wave vector. Over time, wave domains become wider and acquire a cone-like shape (Fig. 6C,D). These changes in the properties of wave domains depend on the parameters of the model. In the central part of the parameter space (when the basal activation rate and negative feedback have intermediate values), a larger number of smaller wave domains coexist within the simulation domain. In the regimes that are closer to the periphery of the region of wave dynamics (where negative feedback is relatively weak or strong), the number of wave domains is smaller, but their size is larger (see Supplementary Figure S11).

Similarly, the strength of the autocatalytic activation, parameter $\gamma$, affects the number and size of wave domains, as illustrated in Supplementary Figure S12A and Supplementary Video S10. Analogously to the (${s}_{2},{k}_{0}$) parameter space, wave domains form in the central part of the (${s}_{2},\gamma$) parameter space, where the excitation level has intermediate values (Supplementary Figure S12B) and the pattern entropy has high values (Supplementary Figure S12C). However, the image correlation doesn’t show as strong of a trend as it does with the changing parameter ${k}_{0}$ (compare Fig. 3D and Supplementary Figure S12D). On the other hand, increasing parameter $\gamma$ leads to patterns with a higher amplitude (Supplementary Figure S12E), while increasing the basal activation ${k}_{0}$ has the opposite effect (Supplementary Figure S4A). Also, the same way as in the (${s}_{2},{k}_{0}$) parameter space, the duration of the low-activity stage is increasing towards the periphery of the Turing-unstable region (Supplementary Figure S12F), where the low-activity stage changes from the quasi-static to the oscillatory type (Supplementary Figure S12G). Finally, the central part of the Turing unstable region with quasi-static low-activity stage is associated with increased number and decreased area of wave domains as illustrated in Supplementary Figure S13 for a range of $\gamma$ values with the fixed value ${s}_{2}=0.9$ and after averaging over 10 simulations for each $\gamma$.

In the experimental data^31^ on starfish oocytes, we also see the progressive increase of the domain sizes (Supplementary Video S1). However, this process does not continue long enough to see a single wave domain winning the whole area. In summary, all the apparent features of the wave domains in our simulations are consistent with the experimental observations that motivated this work.

**Figure 7:**
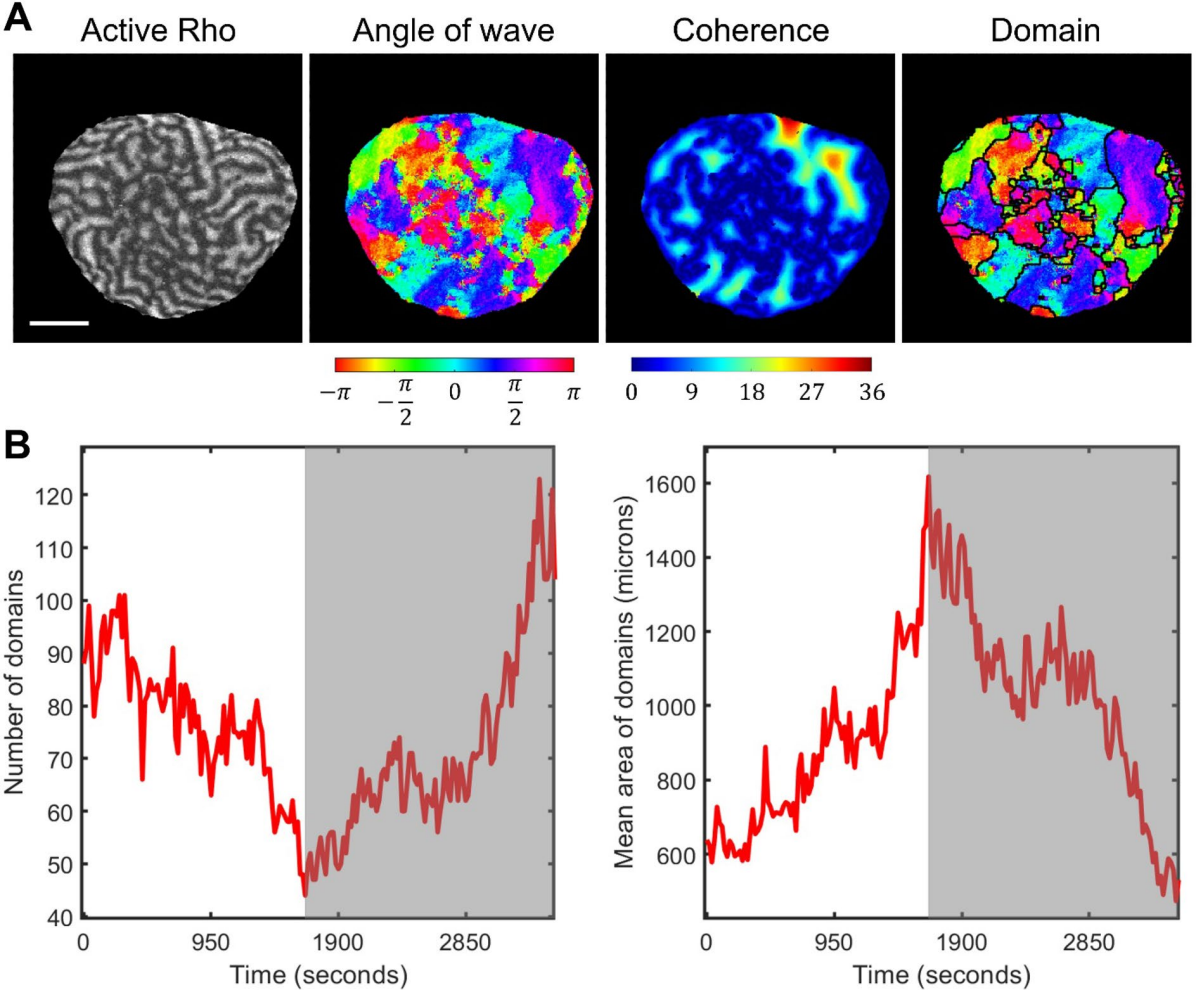
Wave domains analysis for experimental starfish data. (**A**) The pipeline of wave domain identification in starfish experimental images, including active Rho pattern, colormap of wave vector directions, colormap of the coherence distance, and wave domain segmentation. The scale bar is 50 μm. The image in the left panel is generated from the data published in Bement et al*.*^31^ with the permission of the authors. (**B**) The mean size of wave domains as a function of time for starfish oocyte. The grey area indicates the period of time after the sharp switch in the cell behavior.

In addition to the textural characterization, we also applied our automated method of wave domain detection to the starfish experimental data. Figure 7A illustrates the processing pipeline, which includes computing and averaging the wave vector angles, finding the coherence distance, and segmenting it to extract regions of coherent wave propagation (see “Methods”). In agreement with our definition, regions are identified as wave domains if their size is larger than the width of activation front. The lifetime of these wave domains is also larger than the period of oscillation. The initial stage is consistent with the simulation results showing the growth of wave domains’ size and decrease in the number of wave domains (compare Figs. 2H and 7B). However, in the later part of the experimental record, the systems behavior changes abruptly (gray areas in Fig. 7B, see Supplementary Figure S14 for the expanded analysis of additional cells available from Bement et al*.*^31^). The dynamics reverses into increasing the number of wave domains and decreasing the size of domains until the wave propagation completely ceases. The sharp reversal in the domain growth takes place when the average size of the domain is approximately 1000–1500 square microns. Although our model is not built to capture this dynamic reversal, our method of wave domain identification allows us to pinpoint the moment of time when some biological process triggered the change in the system behavior. This behavioral switch could be related to a change in the rate of Cdk1 activity and the rearrangement of cortical microtubules, which may impact the actin dynamics/Rho activity in the oocyte.

## Discussion

Wave domains of actin polymerization and active Rho in the cortex of starfish oocytes may appear as a subtle feature of the cytoskeletal regulation. However, this behavior emerges from a complex intertwined signaling network, and the ability of a computational model to accurately capture such a phenomenon is important for the mechanistic understanding of the underlying processes. In this work, we showed that the development of wave domains has a non-trivial temporal progression. Before wave domains become clearly visible, the system goes through a period of pattern formation with activity level orders of magnitude smaller than the activity at the later stage, the stage that we can compare with the experimental observations of Bement et al*.*^31^. Interestingly, types of patterns in this low-activity stage and the way they become destabilized determines whether we see spiral waves or wave domains. Specifically, wave domains emerge in two cases: (1) when the low-activity patterns are spatially static, have the labyrinth-like organization, and become destabilized through a displacement of the activator and inhibitor peaks along the perimeter of the pattern shapes, and (2) when the low-activity patterns are oscillatory and the transition to high-activity patterns occurs with the preservation of the oscillation phase. In both cases, the strengths of the basal activation and negative feedback have to be balanced so that the total levels of the active and inactive forms of the regulator (e.g., Rho GTPase) are close to each other. In addition to the unstable homogeneous state in the presence of a limit cycle, this balance becomes a necessary requirement for the emergence of the observed wave dynamics. If the balance is broken and the activation starts to dominate over inhibition or vice versa, the wave domains are not formed and instead we see spiral waves.

We make clear distinction between spiral waves and the wave domains by representing Rho activity with a spatiotemporal map of the wave vector directions and applying our segmentation pipeline for automatic detection of the wave domain outlines. In wave domains, the directions are coherent and parallel to the long sides of the domain. When the domains are adjacent to each other at their long side, the waves propagate in opposite directions parallel to the interface between the domains. As opposed to spiral waves, wave domain formation is a higher level of complexity as indicated by the higher textural entropy of the dynamic patterns. Furthermore, in the high-activity stage, the dynamics continue to transform so that domains slowly increase in size and decrease in number. Therefore, in this work, even though we characterized different stages of pattern development, we always dealt with transient dynamics. The experimentally observed dynamics is also transient in the sense that it occurs only during a part of the cell cycle.

In this study we used computational modeling to characterize a particular type of spatiotemporal behavior of a Rho signaling motif and to establish the origin and conditions under which the system can exhibit such behavior. Further study is needed for a more formal and comprehensive mathematical characterization of the system. Previously, various mathematical approaches have been used to predict characteristics of spatial patterns and their dynamics, including Lyapunov Exponents^35^ in general, and Linear Stability Analysis^16^ in particular, as well as Local Perturbation Analysis (LPA)^29^. However, to our best knowledge, there are no approaches developed to analyze non-linear reaction–diffusion systems during the exponential growth of the pattern amplitude following a transition to a qualitatively different dynamic behavior (as described in this work). Recently, Liu et al*.*^34^ (see Fig. 7E,F) reported a bifurcation diagram obtained with LPA for the 1D version of the model that we used for this study. The authors acknowledged that the dynamics regimes of the full system are not exactly aligned with LPA regimes. Thus, further investigation is required to relate the structure of the phase space predicted with LPA to the classification, that we obtained in our work based on the properties of the low-activity stage and the ability of the system to form wave domains.

In our study, we observed oscillatory low-activity stage that transitions into wave propagation. The out-of-phase oscillations that gradually transition into traveling waves are well known. It was described in the work of Meinhardt^47^ in the context of a three-component system and was inspired by the seminal work of Turing^10^, where such out-of-phase oscillations were also predicted. In this three-component model, the activator with autocatalytic properties is coupled with two inhibitors with local and global (long-range) actions. In the same work, Meinhardt discussed the Min system, which is involved in the process of septum localization prior to cell division in bacteria and stated that the mechanism of global inhibition could work through substrate depletion (the concept originally introduced in his earlier works with Gierer^48,49^). Our system also has the depletion of inactive form of Rho, which is a substrate for the autocatalytic component, and the local inhibition with F-actin. However, the model that was proposed by Meinhardt does not include the mass conservation of its components and does not account for the certain basal activation rates. Another difference is that in Meinhardt’s model, the local inhibitor decreases the autocatalytic rate of the activator, while in our model F-actin increases the inactivation rate of active Rho. Finally, in Meinhardt’s work, the patterns were observed for a constant value of the amplitude and, to the best of our knowledge, the properties of the patterns during the exponential amplitude growth were not characterized. The transition of a quasi-static pattern (in the form of spots, reversed spots, or labyrinth) to traveling waves was also not reported before.

Recently, oscillatory patterns that are different from traveling waves were observed in an artificially engineered cell cortex and were referred as coherent oscillations of Rho and F-actin (Supplemental Movie 3 in^50^). However, such oscillatory patterns are drastically different from those that were previously observed in starfish and frog oocytes experiments^31^. Interestingly, a similar dichotomy between patterns observed in living cells and in artificial lipid bilayers is also known in the context of Min system^51^. The relationship of oscillatory patterns in an artificial cortex to the oscillatory low-activity dynamics reported here is an open question for future studies. A model that accurately reproduces wave dynamics in both living cells and artificial bilayer systems can provide insight into the origin of this dichotomy and inform researchers about regulatory pathways and molecular targets that could be modulated to transition between the different dynamic regimes.

Intriguingly, quantifying wave domains characteristics (their sizes and number) over time revealed that, in starfish oocytes, the development of the wave domains agrees with the model up to a point of a sharp dynamic reversal. Some cell-cycle event triggers the switch from increasing the size of the wave domains to decreasing them until the wave propagation stops. The presence of such event was not apparent from the visual inspection but became evident with the quantification of wave domains (Fig. 7B and Supplementary Figure S14). This result emphasizes the power of the concept of wave domains, which serves as a characteristic feature for comparative analysis of the very complex wave dynamics evolving over time on the whole cell level.

We also showed that accounting for intrinsic noise is critical for capturing a range of possible dynamic behaviors in different model organisms or, potentially, in the same organism but under different perturbations. We described the effects of the strong noise on the pattern formation and showed that our model quantitatively reproduces wave dynamics in both starfish (experimentally perturbed to overexpress of Rho-GEF Ect2, induce expression of ∆90 cyclin B, and control the initiation of wave propagation with roscovitine) and frog (wile-type) cells. Previously, Bement et al*.*^31^ showed that the transition between these two phenotypes and increase in the regularity of wave fronts is dependent on positive feedback through Rho-GEF Ect2. Our model predicts that the difference between the two regimes (transition from regular waves that form wave domains to irregular waves like in frog cells) could also be dependent on negative feedback from F-actin.

In this report, we quantitatively characterized spatiotemporal patterns using textural measures, autocorrelations plots, and our novel tool for wave domain detection, but we only scratch the surface of this overlooked phenomenon. In future studies, experimental perturbations that affect either the autocatalytic or negative feedback in the Rho signaling motif could disturb the balance enough to shift the system into different dynamic regimes predicted by the model. This, in turn, could provide an insight into the role of different signaling components in the cytoskeletal regulation. Our results indicate that, in addition to the regulation of Rho activation (e.g., through Ect2, which is a GEF for Rho GTPase^31^), the negative feedback from F-actin plays a key role in regulating the activity patterns, although the molecular mechanisms involved in this feedback are not fully understood yet. Recently, Kamps et al*.*^6^ reported that oscillatory Rho dynamics in motile cells is regulated by negative feedback from myosin, which can be considered as a potential mechanism of inhibition in oocytes. Also, a biological role of the wave domain formation remains to be investigated.

In future studies, the biological community can benefit from our automated wave detection methodology because it is a novel characteristic, which is more intuitive than a set of textural measures, such as image entropy, correlation, and energy. If wave domains are present in a set of imaging data, the statistics of their sizes, shapes, and numbers describe the *geometric* structure of the wave patterns that can be used to explain subtle differences in cell dynamics under different perturbations or under different natural conditions during the cell cycle.

## Methods

### Reaction–diffusion model of Rho GTPases activity

For this study, we have built a two-dimensional reaction–diffusion model based on a one-dimensional version by Holmes et al*.*^27^. GTPase kinetics is represented with the mass-conserved activator-substrate (MCAS) motif that describes switching between the active (GTP-bound) and inactive (GDP-bound) forms with autocatalytic feedback, presumably through guanine nucleotide exchange factors (GEFs). The negative feedback depends on F-actin, which is polymerized in response to the activation of nucleation-promoting factors downstream of Rho signaling. F-actin suppresses Rho activity through a mechanism, which is not fully understood but is assumed in multiple studies^27,31,52^. The minimal signaling model for such a process can be represented as a three-component system:

1 $$\frac{\partial A}{{\partial t}} = f + D_{A} \frac{{\partial^{2} A}}{{\partial x^{2} }} + D_{A} \frac{{\partial^{2} A}}{{\partial y^{2} }} + \alpha_{1} \xi_{1} ,$$

2 $$\frac{\partial I}{{\partial t}} = - f + D_{I} \frac{{\partial^{2} I}}{{\partial x^{2} }} + D_{I} \frac{{\partial^{2} I}}{{\partial y^{2} }} - \alpha_{1} \xi_{1} ,$$

3 $$\frac{\partial F}{{\partial t}} = k_{n} A - k_{s} F + \alpha_{2} \xi_{2} ,$$

where

4 $$f = \left( {k_{0} + \gamma \frac{{A^{3} }}{{A_{0}^{3} + A^{3} }}} \right)I - \left( {s_{1} + s_{2} \frac{F}{{F_{0} + F}}} \right)A$$

and

$$D_{A} = \frac{0.001}{3}, D_{I} = \frac{0.1}{3}, k_{0} \in \left[ {0, 0.3} \right], \gamma = 1, A_{0} = 0.4$$, $$s_{1} = 0.5, s_{2} \in \left[ {0, 1.3} \right], F_{0} = 0.5, k_{n} = 0.1, k_{s} = 0.025,\alpha_{1,2} \in \left[ {10^{ - 15} ,3} \right]$$.

Here, we refer to $A$, $I$, and $F$ as active and inactive forms of a GTPase (or a GEF, or a nucleation-promoting factor in other contexts) and F-actin, for consistency with the experimental study of Rho activity^31^ and the original notations in Ref.^27^. Component $A$ diffuses slowly on the membrane. Its autocatalytic activation is represented with the Hill function $\gamma \frac{{A}^{3}}{{{A}_{0}}^{3}+{A}^{3}}$. Component $I$ diffuses fast in the cytosol. Our model does not account for spatial translocation of actin mesh, so component $F$ has zero diffusion. The negative feedback from F-actin is represented with the Hill function: ${s}_{2}\frac{F}{{F}_{0}+F}$. The activation and deactivation terms also include constant basal rates, ${k}_{0}$ and ${s}_{1}$, respectively. The total amount of $A$ and $I$ is conserved (see more about the mass conservation below), so that the model does not account for changes in GTPase expression level and is suitable for fast processes within the cell cycle.

Equations (1–3) also include noise terms generated as Gaussian random numbers, ${\xi }_{1}$ and ${\xi }_{2}$, with zeros mean and the standard deviation of one, so that ${\alpha }_{\text{1,2}}$ is the magnitude of noise. These terms represent random fluctuations in the number of molecules within the cell area of one pixel. Since the total number of GTPase molecules in the cell is set to be constant, the noise contributions to the equations for $A$ and $I$ have opposite signs. In our simulations, noise with ${\alpha }_{1}={\alpha }_{2}={10}^{-15}$ was sufficient to initiate wave propagation from the unstable homogeneous state. We also used higher magnitudes to reproduce the noisy dynamics in the starfish and frog data (${\alpha }_{1}=3, {\alpha }_{2}=0.1$ in Fig. 1A, Supplementary Figure S6 and ${\alpha }_{\text{1,2}}\in \{{10}^{-15}, \text{1,2},\text{3,4}\}$ in Supplementary Figure S7).

We solve the partial differential Eqs. (1–3) using the forward Euler method (Eq. 5) with time step $\Delta t=0.001$ and spatial step $h=0.02$. Such discretization satisfies the criterion of stability for the difference schemes $r=\frac{D\Delta t}{{h}^{2}}<\frac{1}{2}$, where D is the diffusion coefficient^16,53^. To implement no-flux boundary conditions for an arbitrary cell shape, we applied the modified five-point stencil of the 2D Laplace operator ${L}_{i,j}^{t}$, so that:

5 $$C_{i,j}^{t + 1} = C_{i,j}^{t} + \Delta t\left\{ {f_{i,j}^{t} + \frac{{D_{C} }}{{h^{2} }}L_{i,j}^{t} } \right\},$$

6 $$\begin{aligned} L_{i,j}^{t} & = M_{i + 1,j} \left( {C_{i + 1,j}^{t} - C_{i,j}^{t} } \right) - M_{i - 1,j} \left( {C_{i,j}^{t} - C_{i - 1,j}^{t} } \right) \\ & \quad + M_{i,j + 1} \left( {C_{i,j + 1}^{t} - C_{i,j}^{t} } \right) - M_{i,j - 1} \left( {C_{i,j}^{t} - C_{i,j - 1}^{t} } \right), \\ \end{aligned}$$

where ${C}_{i,j}^{t}$ is the concentration of $A$, $I$ or $F$ in position $(i,j)$ at time $t$, $M$ is the binary mask representing the cell (${M}_{i,j}=1$) and the background (${M}_{i,j}=0$), ${D}_{C}$ is the diffusion coefficient of $A$ or $I$ (while the diffusion of the component $F$ is negligible) and $f$ is the reaction term (see Eqs. 1–4). The discrete Laplace operator ${L}_{i,j}^{t}$ consists of four fluxes. For boundary pixels of the cell mask, the fluxes to the adjacent pixels of the background are multiplied by ${M}_{i,j}=0$, thus, ensuring no loss of concentration through the boundary. The analytical solution of the system (1–4) without noise is guaranteed to be positive as it satisfies the quasi-positivity conditions and Lemma 1.1 in Ref.^54^. Indeed, for $A\ge 0, I\ge 0, F\ge 0$, ${f}_{A=0}={k}_{0}I\ge 0, {-f}_{I=0}=\left({s}_{1}+{s}_{2}\frac{F}{{F}_{0}+F}\right)A\ge 0$, ${k}_{n}A\ge 0$. However, if an independent random variable representing noise is added (${\alpha }_{1}{\xi }_{1}$ and ${\alpha }_{2}{\xi }_{2}$), this condition can be violated. To prevent such violation and guarantee the positivity of the solution at each iteration, our algorithm checks if the added noise brings low concentration to a negative value and resets negative value to zero. To ensure that this reset for one component doesn’t affect mass conservation, the algorithm also adds the overshoot value to the other component.

In the case of a square simulation domain (Fig. 3A, Supplementary Figure S1), we used the square mask (${M}_{i,j}$) of size 202 × 202 with one-pixel-wide padding to define the background, so that the total size of the domain was 200 × 200. The solution was saved every 1000 iterations (1 au of time) and the overall simulation time was 4000 au of time, which is equivalent to 3320 s based on the scaling described in Supplementary Figure S15 and in the Supplementary Text ($1$ au of time is $\text{0.83}$ s, $1$ au of distance is $15.93$ microns). For the frog data, the temporal autocorrelation function is not periodic and, thus, we determine temporal scaling by matching the time intervals where the correlation coefficient drops from 1 to 0.5, which gives $1$ au of time = $\text{1.56}$ s. For the homogeneous initial conditions (Fig. 3A) in simulations with noise (${\alpha }_{\text{1,2}}={10}^{-15}$), we used the values of reagents: ${A}_{i,j}^{0}=0, {I}_{i,j}^{0} =1$,${F}_{i,j}^{0}=0$, for all grid points $i,j\in [\text{2,201}$] of the simulation domain. In simulations with no noise and initial excitation in the center of the simulation domain of the size 202 × 202 (Supplementary Figure S1), we used the initial conditions $${A}_{i,j}^{0}=\left\{\begin{array}{c}5, i,j\in [\text{96,105}]\\ 0, i,j\notin [\text{96,105}]\end{array}\right.$$, ${I}_{i,j}^{0}=1-\frac{{\sum }_{i,j}{A}_{i,j}^{0}}{{200}^{2}}$, and ${F}_{i,j}^{0}=0$, for $i,j\in [\text{2,201}]$. In Fig. 1B, we used the mask (${M}_{i,j}$) with the irregular cell shape extracted from the experimental image. The bounding square of the cell mask was resized to 659 × 659 pixels (the whole mask is 793 × 793 pixels) so that, based on the estimated scaling factors and the selected spatial step, the size of the cell in the simulation (~ 210 microns) was consistent with the experimental data. In this case, the initial conditions were homogeneous as in Fig. 3A ${A}_{i,j}^{0}=0, {I}_{i,j}^{0} =1$,${F}_{i,j}^{0}=0$ for $i,j\in [\text{0,793}$] and ${M}_{i,j}=1$.

To verify the positivity of our numerical solutions with and without noise directly, we computed minimal and maximal values of concentrations for each simulation in Fig. 3A and Supplementary Figure S1 across all saved states (see Supplementary Figures S16, S17). There were no cases when the value of concentration was negative. We also verified that the total Rho mass is conserved with the precision over $1\pm {10}^{-11}$ of the total initial Rho mass in all our simulations with and without noise (Supplementary Figure S18). See also Supplementary Figure S19 for examples of the concentration changes over time in different parameters regimes and for different values of noise. To verify the stability of our finite difference scheme, we compared the solution with different time steps, ${\Delta t=10}^{-3}$ and $\Delta t={10}^{-5}$ au (Supplementary Figure S20). In these two cases, simulations were performed without noise but with identical random perturbation in the initial conditions: ${A}_{i,j}^{0}={10}^{-4}\left|{\xi }_{i,j}\right|, {I}_{i,j}^{0} =1-{10}^{-4}|{\xi }_{i,j}|$,${F}_{i,j}^{0}=0$ for $i,j\in [\text{1,201}$] and ${M}_{i,j}=1$, where the mask is the same as in Fig. 3A and ${\xi }_{i,j}$ is Gaussian random variable with zero mean and standard deviation equal to one.

We also verified the oscillatory behavior in our solution by an alternative PDE solver based on the Finite Elements Method as implemented in MATLAB PDE Toolbox (see Supplementary Text and Supplementary Figures S21–S24). In this verification, we used the square domain of size 4 × 4 au (the same as in Fig. 3A and in Supplementary Figure S1) and triangulated it with the maximal length edge of 0.05 au. The initial conditions were chosen with random perturbation in each node of the grid: ${A}_{i,j}^{0}={10}^{-6}\left|{\xi }_{i,j}^{1}\right|, {I}_{i,j}^{0} =1-{10}^{-6}|{\xi }_{i,j}|$,${F}_{i,j}^{0}={10}^{-6}\left|{\xi }_{i,j}^{2}\right|$. Finally, to check the sensitivity of the formation of wave domains to the geometry of a simulation domain, we performed simulations with three different masks: square with 1 pixel padding, circle, and irregular shape (Supplementary Figure S25). Here, the overall size of the simulation domain is 602 × 602, the initial conditions are homogeneous (${A}_{i,j}^{0}=0, {I}_{i,j}^{0} =1$,${F}_{i,j}^{0}=0$ for $i,j\in [\text{0,602}$] if ${M}_{i,j}=1$), and the noise values are small (${\alpha }_{\text{1,2}}={10}^{-15}$).

The described implementation of the PDE solution is available at [https://github.com/tsygankov-lab/WaveDomains].

### Characterization of system’s dynamics over the course of pattern development

To visualize patterns with the amplitude growing exponentially from a homogeneous state, we scaled the solution as ${A}_{scaled}=\frac{A-{\text{min}}\left(A\right)}{{\text{max}}\left(A\right)-{\text{min}}\left(A\right)}$. For kymographs representing the dynamics of the system across the secant line of the domain, the solution was scaled as ${A}_{scaled}=\frac{A-{\text{mean}}\left(A\right)}{{\text{std}}\left(A\right)}$ to show the normalized deviation of the concentration from the mean value. The duration of the stage of rapid amplitude growth, ${T}_{g}$, was measured as the period of linear increase in the log-transformed amplitude ${a}_{l}\left(t\right)={\text{log}}\left(\underset{i,j}{\text{max}}\left({A}^{t}\right)-\underset{i,j}{\text{min}}\left({A}^{t}\right)\right)$, such that $\frac{d}{dt}{a}_{l}(t)>0.001\frac{1}{au\; of \; time} (>0.0083\frac{1}{s} )$.

The amplitude of the pattern at the end of low-activity stage in Supplementary Figure S4A was plotted at the time ${T}_{g}$.

### Temporal autocorrelation analysis

To analyze pattern dynamics, we used temporal autocorrelation using Pearson’s linear correlation (see Supplementary Figure S2A) coefficient as:

$$C^{t} \left( \tau \right) = \mathop {{\text{corr}}}\limits_{i,j} \left( {A_{i,j}^{t} ,A_{i,j}^{t + \tau } } \right) = \frac{{\mathop \sum \nolimits_{i,j \in D} \left( {A_{i,j}^{t} - \overline{A}^{t} } \right)\left( {A_{i,j}^{t + \tau } - \overline{A}^{t + \tau } } \right)}}{{\sqrt {\mathop \sum \nolimits_{i,j \in D} \left( {A_{i,j}^{t} - \overline{A}^{t} } \right)^{2} \cdot \mathop \sum \nolimits_{i,j \in D} \left( {A_{i,j}^{t + \tau } - \overline{A}^{t + \tau } } \right)^{2} } }} .$$

Here, the pattern at time $t$, ${A}_{i,j}^{t}$, is compared with the pattern after a time lag $\tau$, ${A}_{i,j}^{t+\tau }$, and ${\overline{A} }^{t}$ is the average value of $A$ at time $t$ over the whole simulation domain $D$. The correlation coefficient increases or decreases in the range $[-1, 1]$ as the patterns at time $t$ and $t+\tau$ overlap more or less with each other, respectively. Next, we plot the correlation coefficients as functions of the time lag $\tau$. In the case of quasi-static behavior, the temporal autocorrelation coefficient is close to a constant value of one, while in the case of oscillatory behavior, it exhibits periodic oscillations. A regime was classified as oscillatory if the difference between the maximum and minimum values of the temporal autocorrelation coefficient in the window $\tau \in [\text{0,0.4}{T}_{g}]$ from $t=0.5{T}_{g}$ (where ${T}_{g}$ is the duration of low-activity stage defined above) was bigger than one:

$$\mathop {\max }\limits_{\tau } C^{t} \left( \tau \right) - \mathop {\min }\limits_{\tau } C^{t} \left( \tau \right) > 1.$$

We have chosen the values of $\tau$ and $t$ so that the window starts after the low-activity patterns are formed ($t=0.5{T}_{g}$) and ends before the high-activity stage is reached ($t+\tau <0.9{T}_{g}$).

Using these autocorrelation functions, we estimated the period of oscillations in the low-activity stage by computing the mean distance between peaks of the function. The locations of the peaks were determined with the built-in MATLAB function *findpeaks*. The results of this analysis are included in Supplementary Figure S4B.

### Quantitative analysis of wave propagation using textural features

To compare regimes of wave propagation, we applied the following texture analysis. The experimental data was preprocessed with ImageJ^55^ to crop the frame of cell cortex of 100 × 100 μm. The experimental and simulation data were then resized to have identical resolution of 100 × 100 pixels. Within the time window that was used for the analysis, the data was scaled as ${A}_{scaled}=\frac{A-\text{mean}\left(A\right)}{\text{std}\left(A\right)}$. The image entropy was computed with the MATLAB function *entropy*. The image contrast, correlation, energy, and homogeneity were computed with the function *graycoprops(GCM)*, where the gray-level co-occurrence matrix GCM was computed with the function *graycomatrix*. We also used a measure of the excitation level, which was computed as the fraction of the area, where the concentration of the activator is higher than its mean value. The dimensionality reduction and visualization were performed with the MATLAB functions *pca* and *biplot*. The pipeline for the full texture analysis is shown in Supplementary Figure S26.

### Characterization of the spatial distribution of the direction of wave vectors

To compute the directions of wave vectors from the time-series data and to estimate the direction of wave propagation, we compared the state of the system at each pixel $({x}_{i},{y}_{i})$ at time $t$ with the pixels $({x}_{j},{y}_{j})$ in the convolution window $W$ around the pixel $({x}_{i},{y}_{i})$ at time $t+\tau$. The similarity of pixel $({x}_{i},{y}_{i})$ with pixels $({x}_{j},{y}_{j})$ was computed based on the difference in the concentration |${A}_{{x}_{i},{y}_{i}}^{t}-{A}_{{x}_{j},{y}_{j}}^{t+\tau }|$ and in the spatial Sobel gradient $\left|\text{grad}\left({A}_{{x}_{i},{y}_{i}}^{t}\right)-\text{grad}\left({A}_{{x}_{j},{y}_{j}}^{t+\tau }\right)\right|$ (see Supplementary Figure S2B,C). Thus, we defined the vector of wave propagation as a weighted sum of vectors pointing from $({x}_{i},{y}_{i})$ to $({x}_{j},{y}_{j})$:

$$\vec{k}\left( {x_{i} ,y_{i} } \right) = \mathop \sum \limits_{j \in W} \frac{{\vec{r}\left( {x_{j} ,y_{j} } \right) - \vec{r}\left( {x_{i} ,y_{i} } \right)}}{{\left( {1 + \left| {A_{{x_{j} ,y_{j} }}^{t + \tau } - A_{{x_{i} ,y_{i} }}^{t} } \right| + \left| {{\text{grad}}\left( {A_{{x_{j} ,y_{j} }}^{t + \tau } } \right) - {\text{grad}}\left( {A_{{x_{i} ,y_{i} }}^{t} } \right)} \right|} \right)^{n} }}.$$

Here $\overrightarrow{r}\left({x}_{i},{y}_{i}\right)$ is a radius vector from the origin to the pixel with coordinates $({x}_{i},{y}_{i})$ and $n$ is a positive number. As a power of the sum of 1 and two positive numbers, the value of the denominator is always larger than 1. Higher values of $n$ help to sharpen the difference in the contribution of pixels from the convolution window that are more or less similar to the $i$th pixel in terms of the concentration values and the gradient of the concentration. We used the value $n=5$, however, the final results of our segmentation pipeline turned out to be not sensitive to this parameter (Supplementary Figure S27). For our simulated data with a small value of noise ($\alpha ={10}^{-15}$), we used 5-pixel convolution window (${w}_{c}$) and the time lag ${\tau }_{c}=8$au. The computed values of wave vector angles, $\theta$, were averaged with the running window of ${T}_{av}=300$ frames (300 au or 249 s). For the analysis of starfish oocyte data, we used 5-pixel convolution window and $\tau =1$ frame (19 s) and time averaging over 15 frames. The average values were computed using the circular mean^43^. For the visualization, we plotted the angles, $\theta$, for each image pixel with the cyclic HSV (hue, saturation, and value) colormap, which generates the same color for the lowest value of the colormap (zero) as for its highest value ($2\pi$). To visualize the magnitude of a local change in the direction of wave vectors, $\theta$, we used Sobel gradient of the image of values $\left(\text{sin}\theta +\text{cos}\theta \right)$. Finally, for the analysis of experimental data with moving cell edge, we extracted the overlapping part of cell masks within the averaging time window.

### Frog embryo, microinjection, and microscopy

Ovulation of *Xenopus laevis* was induced by injecting 0.5 ml Chorionic Gonadotrophin (1000 U/ml, Sigma) into the dorsal lymph sac of the female animal the night before. On the second day, eggs were gently squeezed into a petri dish and mixed with a small piece of testes isolated from male frogs. After fertilization, eggs were dejellied in 3% cysteine in 0.1 × MMR (Marc’s Modified Ringer’s, 100 mM NaCl, 2 mM KCl, 2 mM CaCl_2_, 1 mM MgCl_2_, 5 mM HEPES, pH7.4) and washed in 0.1 × MMR. For microinjection, embryos were changed into 3% Ficoll solution in 0.5 × MMR. Embryos were injected at 1–2 cell stage with 0.5 ng of EGFP-Utrophin into the animal pole. Injected embryos were maintained at 18C for 2–3 h till they reached stage 6 (32-cell stage), before fertilization membrane was carefully removed by a pair of forceps. The embryos were then mounted into glass bottom dish filled with 0.1 × MMR and positioned with animal pole facing the cover glass. The embryos were hold into position by a small piece of cover glass raised by a ring of silicone grease. Imaging was acquired with PerkinElmer Spinning Disk Confocal microscope using a 60× (1.49 NA) objective. Around 4 µm z-stacks were captured at about 1.7 s intervals for 15 min.

All protocols in this study were carried out in accordance with the recommendations of United States Department of Agriculture (USDA) Animal Welfare Act Regulations and Public Health Service Policy and is approved by Georgia Institute of Technology Institutional Animal Care and Use Committee (IACUC number A100001) and Georgia Institute of Technology Biosafety Committee (IBC number R18021). The present animal investigation was conducted in consideration of the National Institutes of Health guide for the care and use of Laboratory animals (NIH Publications No. 8023, revised 1978) and complied with the ARRIVE guidelines.

**Table 1:** Parameters of the model.

Notation	Description	Value
${D}_{A}$	Diffusion coefficient of active Rho	$\frac{0.001}{3}\left(\frac{\upmu{\text{m}}^{2}}{\text{s}}\right)$
${D}_{I}$	Diffusion coefficient of inactive Rho	$\frac{0.1}{3}\left(\frac{\upmu {\text{m}}^{2}}{\text{s}}\right)$
$${k}_{0}$$	Basal activation rate of Rho	$$[0, 0.3]$$$$\left(\frac{1}{\text{s}}\right)$$
$\gamma$	Rate constant of autocatalytic activation of Rho	$1\left(\frac{1}{\text{s}}\right)$
${A}_{0}$	Parameter controlling the sensitivity of positive feedback of Rho to the concentration of active Rho	$0.4 {G}_{T}$
${s}_{1}$	Basal deactivation rate of Rho	$0.5\left(\frac{1}{\text{s}}\right)$
${s}_{2}$	Rate constant of negative feedback from F-actin on Rho	$\left[0, 1.3\right]\left(\frac{1}{\text{s}}\right)$
${F}_{0}$	Parameter controlling the sensitivity of negative feedback of Rho to the concentration of F-actin	$0.5{ G}_{T}$
${k}_{n}$	Rate constant of F-actin polymerization	$0.1\left(\frac{1}{\text{s}}\right)$
${k}_{s}$	Rate constant of F-actin depolymerization	$0.025\left(\frac{1}{\text{s}}\right)$
${\alpha }_{\text{1,2}}$	Magnitude of noise	$[{10}^{-15},3] \left(\frac{\text{mol}}{\text{L s}}\right)$
$\Delta t$	Temporal step of finite difference scheme	$0.001$, ${10}^{-5}$ au ($1.8\times {10}^{-3}$ s, $1.8\times {10}^{-5}$ s)
$h$	Spatial step of finite difference scheme	$h=0.02$ au (0.3186 μm)
${\tau }_{sc}^{1}$	Temporal scaling factor for starfish experiment (see Supplemental Text for scaling method)	$\text{0.83}$ s
${\tau }_{sc}^{2}$	Temporal scaling factor for frog experiment	$\text{1.56}$ s
${r}_{sc}$	Spatial scaling factor	$15.93$ microns
$d$	Size of the simulation domain	4–14 au
$$\frac{d}{dt}{a}_{l}(t)$$	Threshold value for the slope of pattern amplitude in log scale defining the duration of the low-activity stage	$$0.001\frac{1}{au \; of \; time}$$$$(0.0083\frac{1}{\text{s}} )$$
${T}_{g}$	Duration of the low-activity stage	500–2471 s
${w}_{c}$	Size of the convolution window for calculating wave vector directions	5 pixels
${\tau }_{c}$	Time lag between frames for calculating wave vector directions	8 au (6.64 s)
${T}_{av}$	Time averaging window for calculating wave vector directions	300 au (249 s)
${C}_{cr}$	Threshold value for the circular standard deviation of wave vector directions defining the coherence distance	$0.4$
${\theta }_{m}$	Threshold value for the difference in mean values of wave vector directions defining the regions for merging	0.5 radians
${l}_{cr}$	Threshold value for the length of the interface between neighboring domains defining the regions for merging	10% of $\sqrt{\text{the \; area \; of \; the \; smaler domain}}$

## Supplementary Information

Supplementary Video 1.

Supplementary Video 2.

Supplementary Video 3.

Supplementary Video 4.

Supplementary Video 5.

Supplementary Video 6.

Supplementary Video 7.

Supplementary Video 8.

Supplementary Video 9.

Supplementary Video 10.

Supplementary Information.

## Data Availability

https://github.com/tsygankov-lab/WaveDomains.
