# Supplementary material for: Spatiotemporal development of coexisting wave domains of Rho activity in the cell cortex
Source: Sci Rep. 2021 Sep 30;11:19512. doi: 10.1038/s41598-021-99029-x (PMC8484676; doi:10.1038/s41598-021-99029-x)
Supplement: Supplementary file 11 — Supplementary Information. [file 41598_2021_99029_MOESM11_ESM.docx]

Supplemental Information for

**Spatiotemporal development of coexisting wave domains of Rho activity in cell cortex**

Siarhei Hladyshau^1,2^, Mary Kho^1^, Shuyi Nie^1^, Denis Tsygankov^2^*

^1^School of Biology, Georgia Institute of Technology, Atlanta, GA.

^2^Wallace H. Coulter Department of Biomedical Engineering, Georgia Institute of Technology and Emory University, Atlanta, GA.

*To whom correspondence should be addressed

Email: denis.tsygankov@bme.gatech.edu

**This PDF file includes:**

Figures S1 to S27

Supplemental Text

**Other supplementary materials for this manuscript include:**

Supplemental Videos S1 to S10.


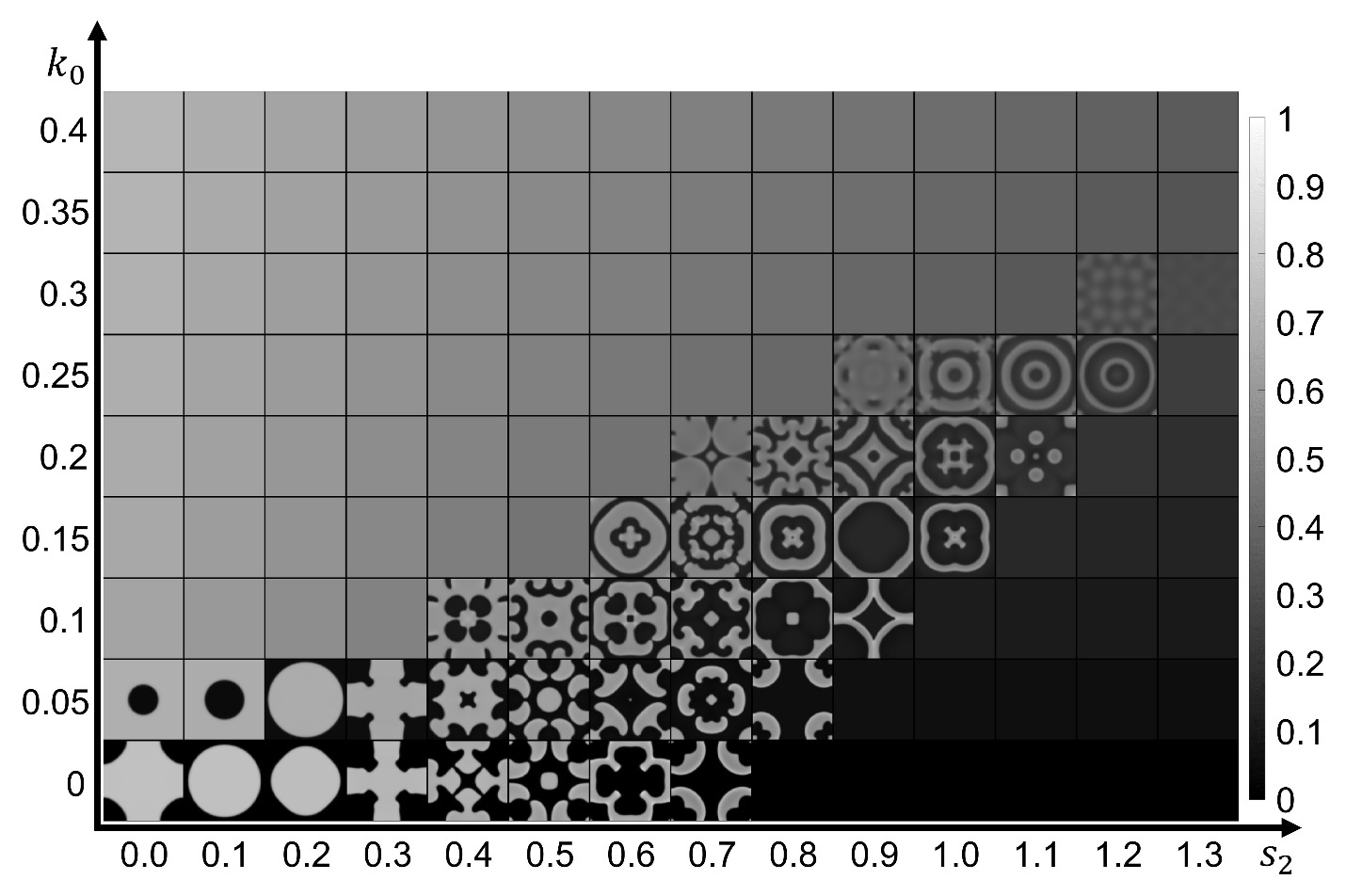


**Supplemental Figure S1.** The result of our model simulations with nonhomogeneous initial conditions for a range of parameters $k_{0}$ (the rate of basal activation) and $s_{2}$ (the strength of negative feedback). The initial spike of excitation of component *A* in the middle of the simulation domain had the magnitude of 5 units of total GTPase concentration and the size 10x10 grid steps (0.2x0.2 au, 3.19x3.19 microns). All simulations were performed on a square domain of size 200x200 grid steps (4.0x4.0 au, 63.7x63.7 microns) with a time step 0.001 au ($8.3\cdot{10}^{-4}$ s) and no-flux boundary conditions.


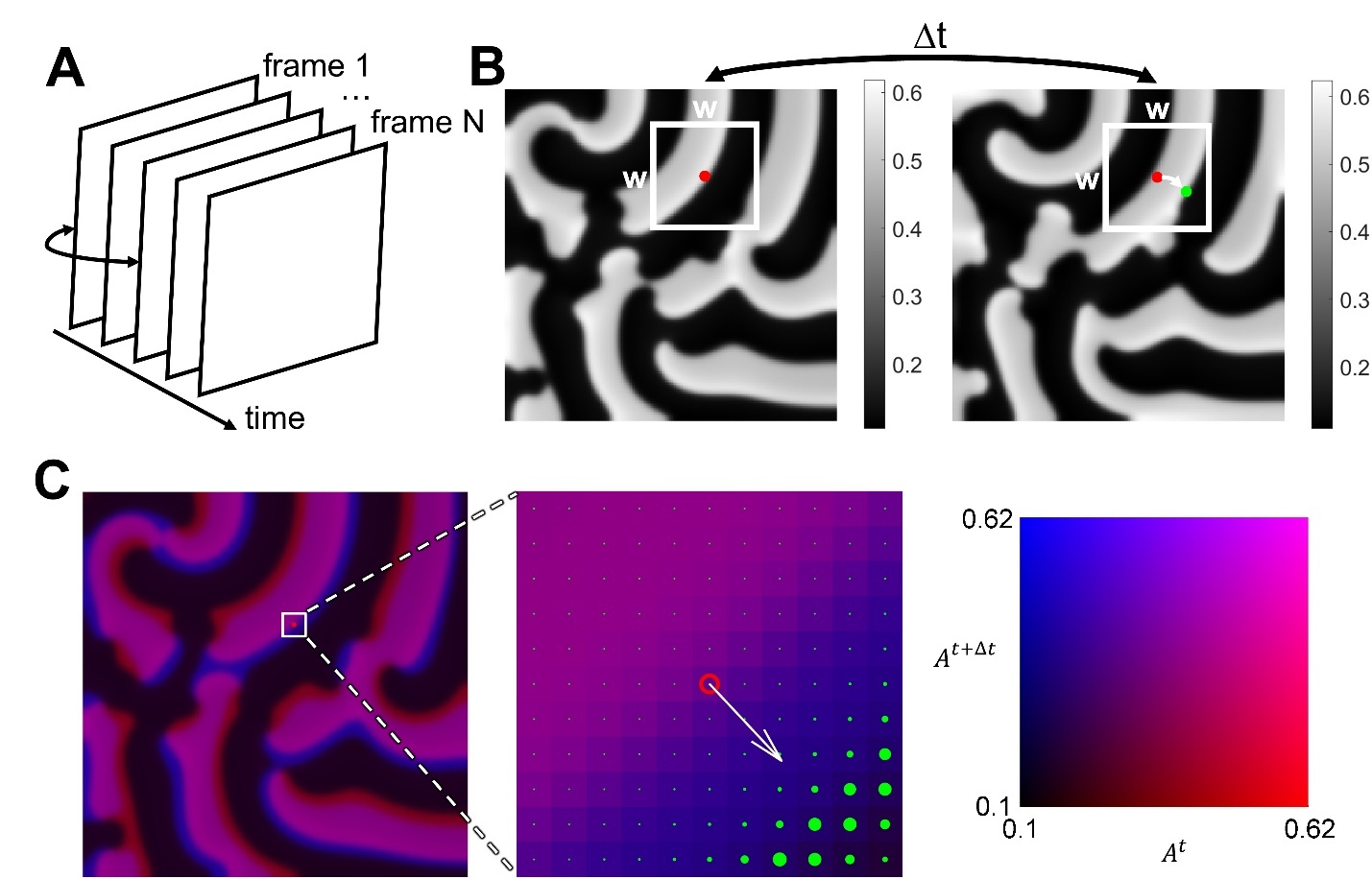


**Supplemental Figure S2.** **A.** Temporal autocorrelation analysis for the time-series data. For the concentration of activator $A$, we compute the correlation coefficients between a frame and the one that follows it after a time lag. To represent the dynamics of the pattern over time, we plotted the computed correlation coefficients as a function of time lag between frames. **B.** The algorithm for calculating the wave vector direction. For each node on the grid at the current state of the system (red dot) we calculate the weighted displacement of each node on the grid of the next time frame (green dot) within the convolution window of width $w$ (white box). The weights depend on the difference in the concentration values and the concentration gradient (see the mathematical expression in **Methods** of the main text). **C.** The size of green dots in the convolution window represents the magnitude of the weights (a measure of similarity) used in the convolution operator. The images are generated by overlapping two channels: the red channel represents the current values of activator concentration, while the blue channel represents the concentration of activator after the time lag. The right panel shows numerical values corresponding to the color scale.


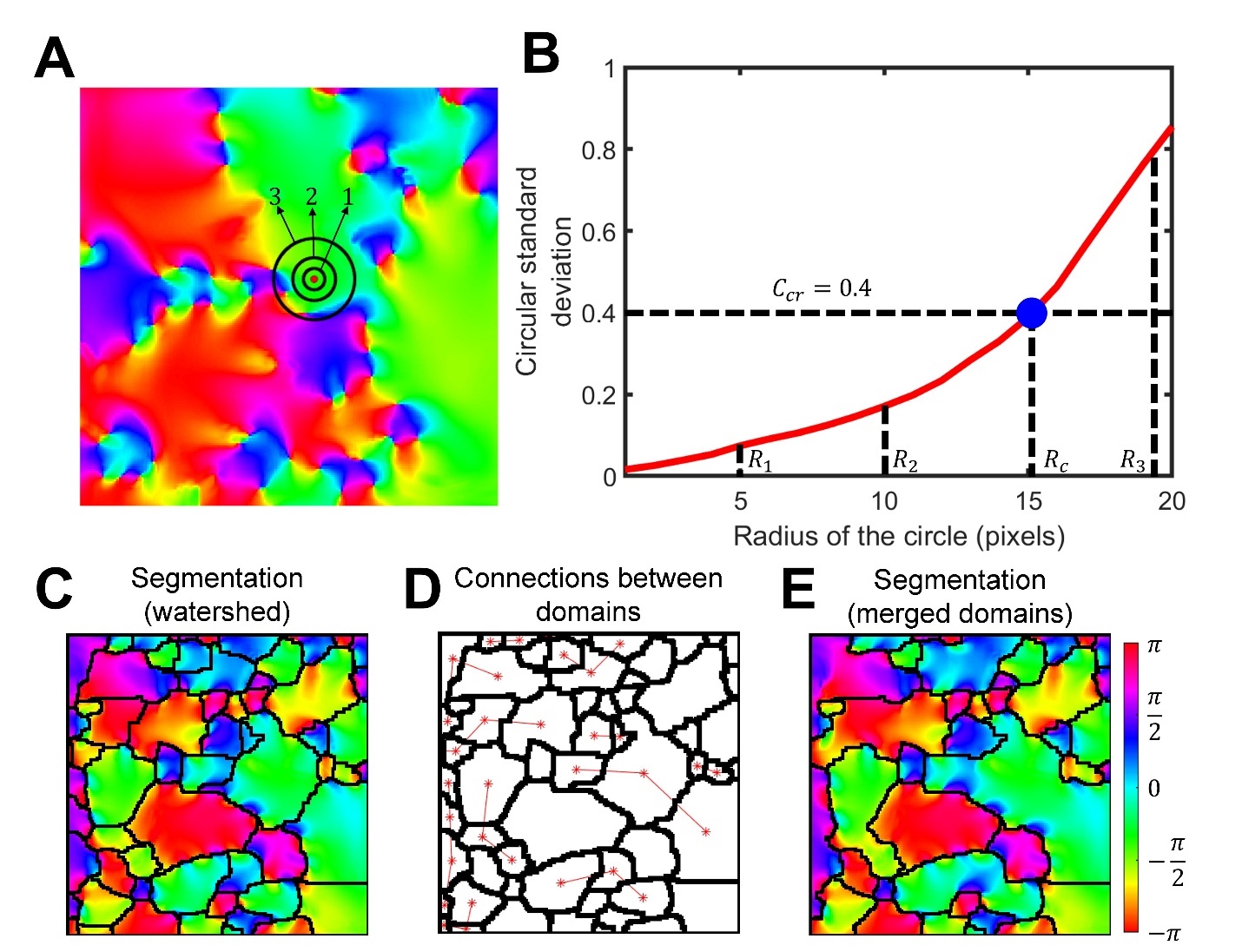


**Supplemental Figure S3.** **A, B.** A measure of coherence distance. For each grid point, we find the standard deviation of wave vector directions (see **Methods**) within a circle centered at the grid point as a function of the radius of the circle, $R$. **A.** An example of a grid point (red dot) and three circles of radius $R_{1}$, $R_{2}$, and $R_{3}$. **B.** The standard deviation as a function of radius $R$. The radius at which the standard deviation reaches a critical value $C_{cr}$ (blue dot), is defined as the *coherence distance*. The larger is the region with coherent propagation of waves, the larger is the coherence distance at the center of this region. **Fig. 2F** shows the coherence distance calculated for all grid points, $\left[ R_{C} \right]_{ij}$. Such coherence distance maps were used at the next step of the segmentation pipeline, Watershed Segmentation. In our analysis, we used the value of the critical parameter $C_{cr}=0.4$ that provided the most reliable (most consistent with a direct visual assessment) segmentation of the wave domains. **C, D, E.** The pipeline for the region merging algorithm to correct over-segmentation. **C.** Watershed algorithm applied the coherence distance map. **D.** To correct over-segmentation, we post-process watershed output and perform merging of domains. The plot shows the graphical representation of the connected domains as specified by the adjacency matrix $A=(a_{ij})$ build based on the pairwise similarity of the domains. Here, $a_{ij}=1$ if the difference between the mean values of the wave vector angles in domains $i$ and $j$ is less than 0.5 radians and if the length of the interface between the domains is larger than 10% of the square root of the area of domains are merged by removing the smallest of domains $i$ and $j$ . Otherwise, $a_{ij}=0$. **E.** All the groups of connected interfaces between the adjacent domains while maintaining the 4-connectivity of the merged regions.


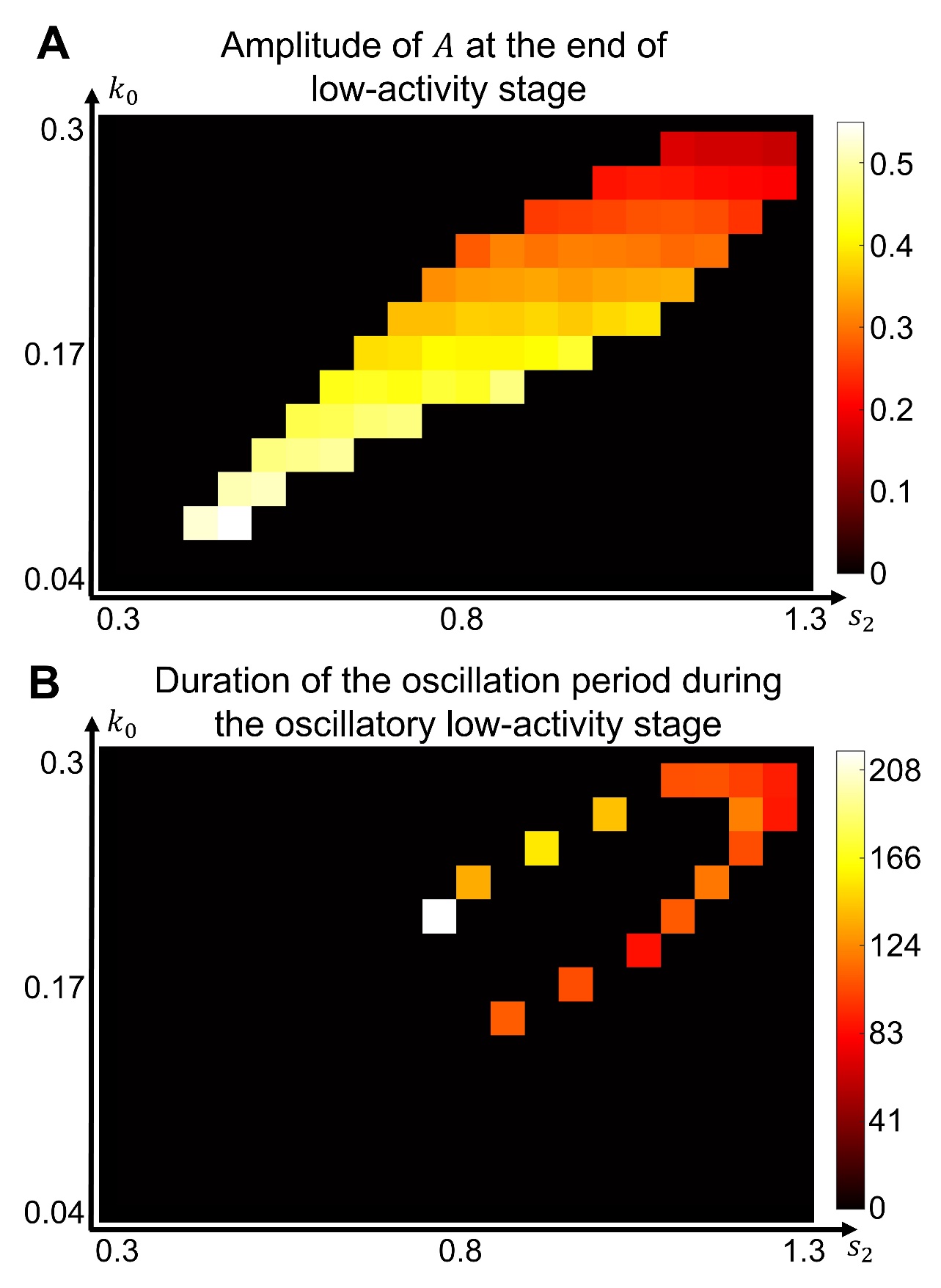


**Supplemental Figure S4.** **A.** The colormap representing the value of activator amplitude at the end of low-activity stage (see **Methods “Characterization of system’s dynamics over the course of pattern development”** for details), calculated for each simulation in the ${(s}_{2}$, $k_{0})$ parameter space. **B.** The colormap representing the period of activator oscillations (in seconds) during the oscillatory low-activity stage, calculated for each simulation in the ${(s}_{2}$, $k_{0})$ parameter space (see **Methods “Temporal autocorrelation analysis for details”**).


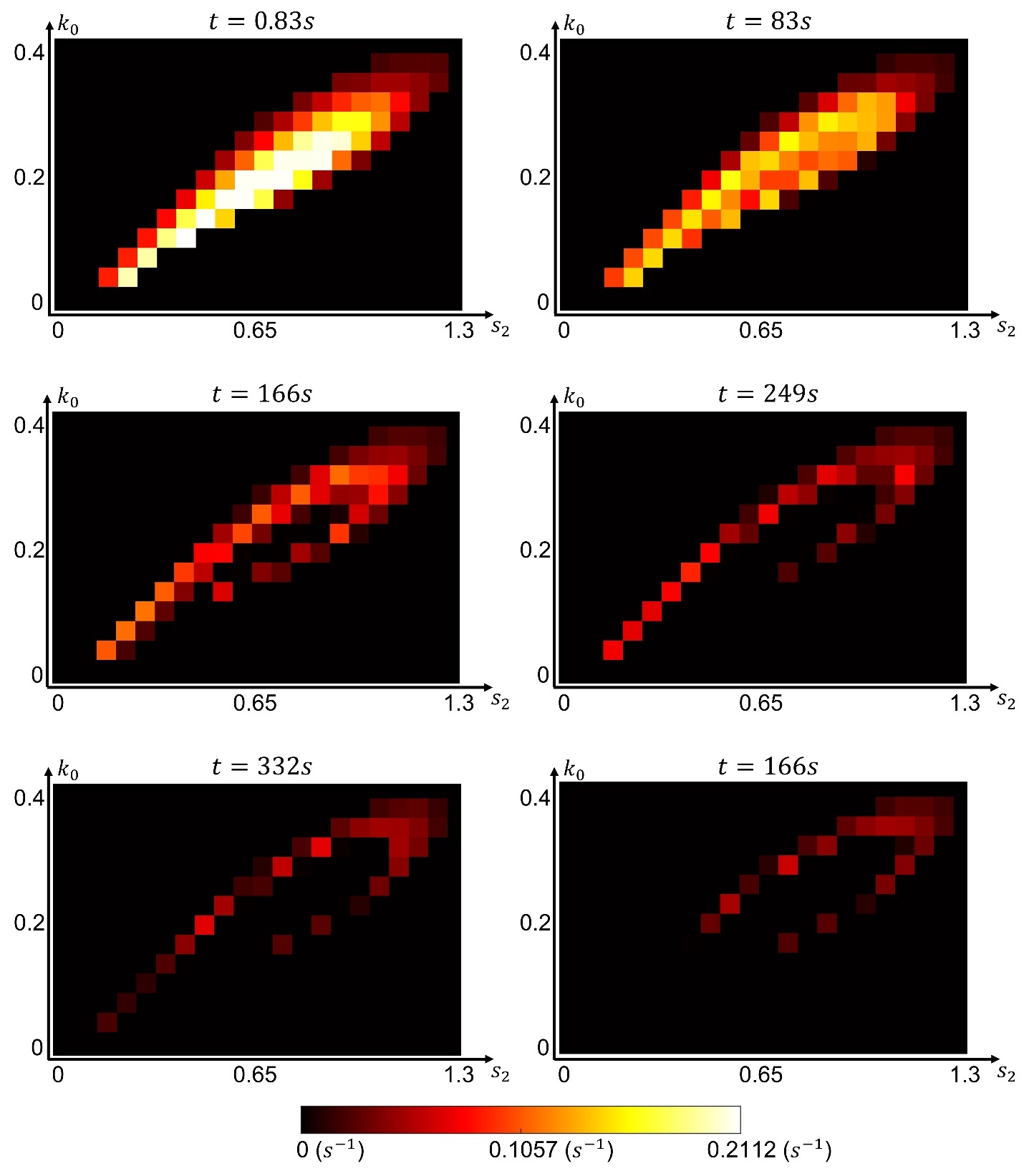


**Supplemental Figure S5.** The averaged slope of the pattern’s amplitude growth in the log scale for different values of the basal activation of Rho and negative feedback from F-actin (the slope values were smoothened with running average using the window of 166 s). This parameter was used to distinguish the low activity stage, during which the amplitude grows exponentially (linearly in the log scale), from the high activity stage, during which the amplitude remains nearly constant. At the beginning of simulations ($t=0.83s$), the slope is higher in the central part of the Turing-unstable region of the parameter space. Over the course of time, the slope decreases (see $t=83 s, 166 s, 249 s$) depending on the parameters. The regimes that are closer to the periphery of the Turing-unstable region of the parameter space have lower slopes and longer durations of the low-activity stage.


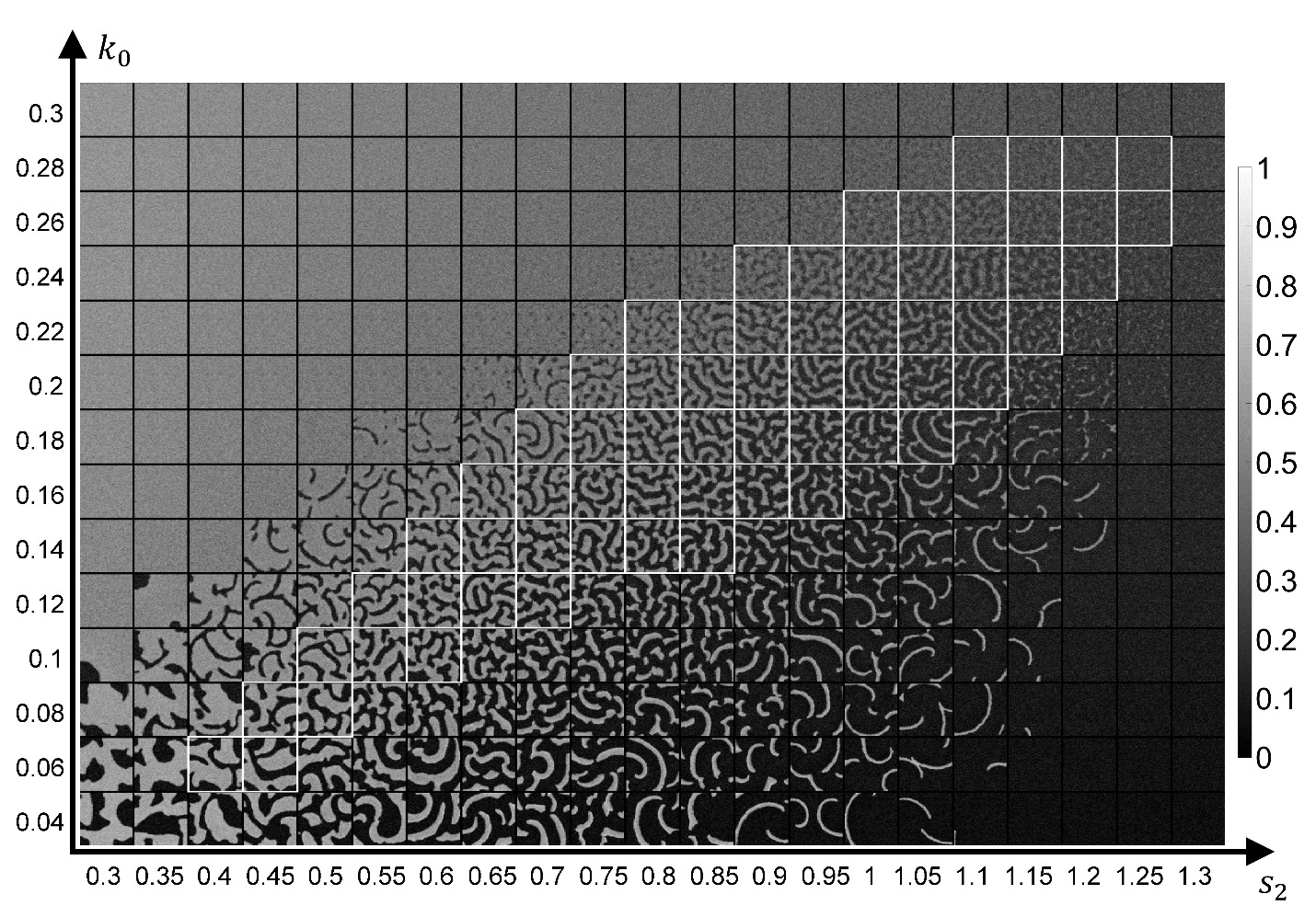


**Supplemental Figure S6.** The result of model simulations of active Rho for a range of parameters $k_{0}$ and $s_{2}$ (see also **Supplemental Video S7**) with high values of noise. The white outline represents the parameter regimes that form high-activity patterns with a minimal value of noise (see **Fig. 3A**).


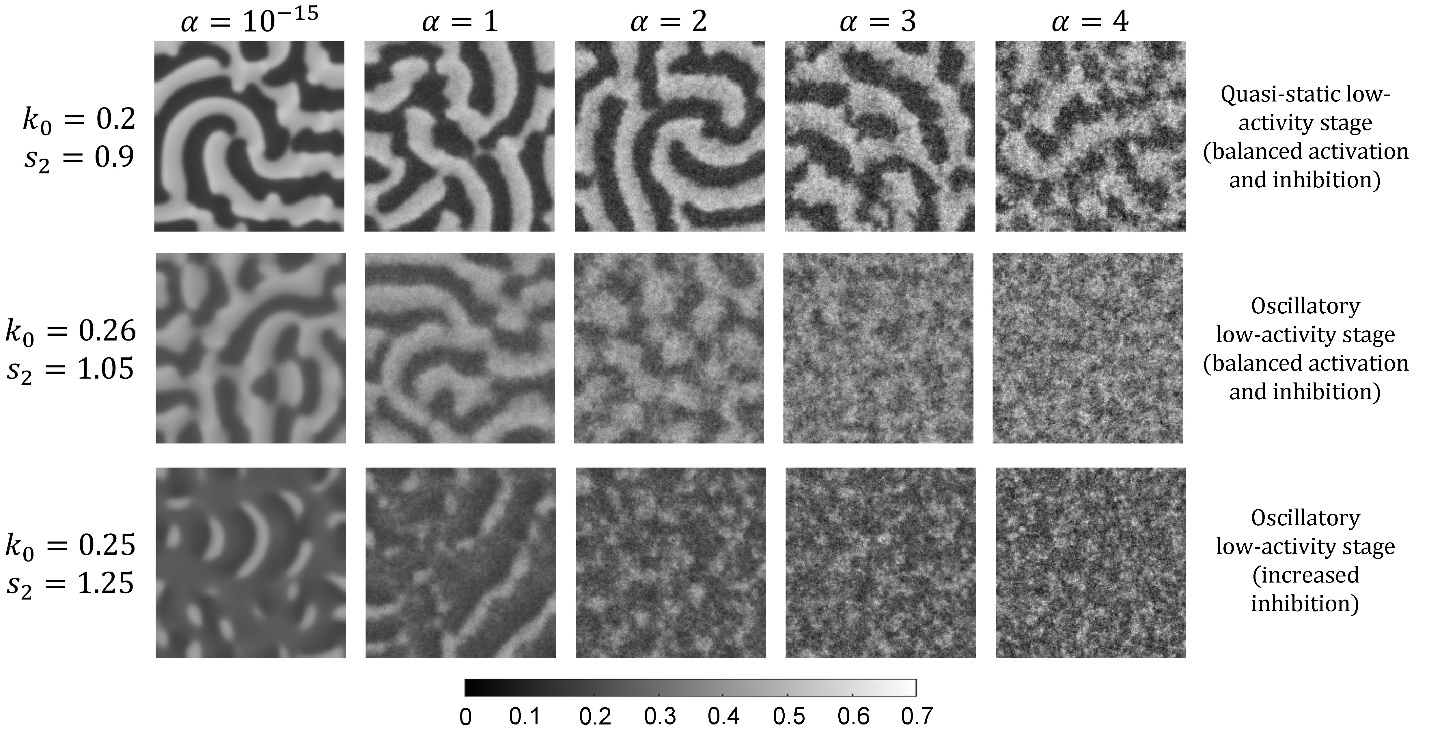


**Supplemental Figure S7.** The result of model simulations of active Rho for different values of noise ($\alpha_{1}=\alpha_{2}=\alpha$) in the cases of quasi-static low-activity stage (top row) and oscillatory low-activity stage with balanced (middle row) and increased (bottom row) inhibition. The parameter regime with quasi-static low-activity stage (white region in **Fig. 6A**) has higher amplitude of the patterns and generate wave domains that are more robust to high values of noise (resembling Rho dynamics in the starfish data) than it is in regimes with oscillatory low-activity stage (blue region in **Fig. 6A**) where wave domains are disrupted by noise (resembling Rho dynamics in the frog data).


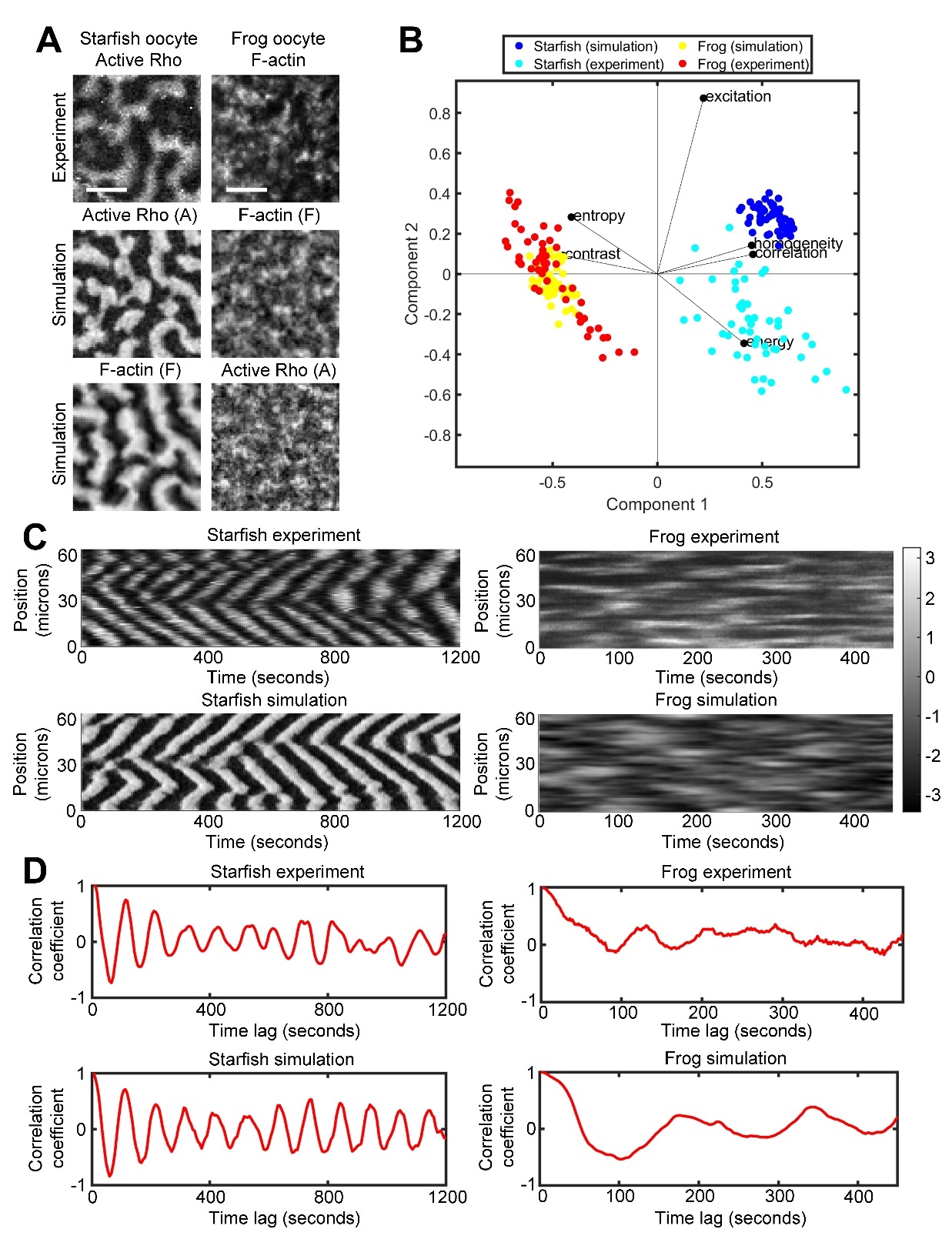


**Supplemental Figure S8. Quantitative analysis of active Rho and F-actin patterns in simulations and experiments (frog blastomere during the early stage of embryo development and starfish oocyte data from Bement *et al.* [31]). A.** Upper panels: examples of starfish data (active Rho) and our frog data (F-actin). Central and lower panels: the corresponding simulation results representing both active Rho and F-actin patterns. The scale bar for all panels in A is 20 microns. The experimental data for starfish is generated from (Supplementary Video 16 in [31] with the permission of the corresponding author). The presented regions of the cell from the experiment and the simulation domain have the size of 64x64 microns. **B.** Biplot representing the results of PCA analysis of the simulated and experimental patterns. Active Rho signal was used for the starfish data and F-actin for the frog data. 50 consecutive time frames were used from each dataset. **C.** Representative kymographs from the experimental data and the simulations. The patterns are scaled to have zero mean and standard deviation equal to one. **D.** Temporal autocorrelation plots for the experimental data and the simulation.


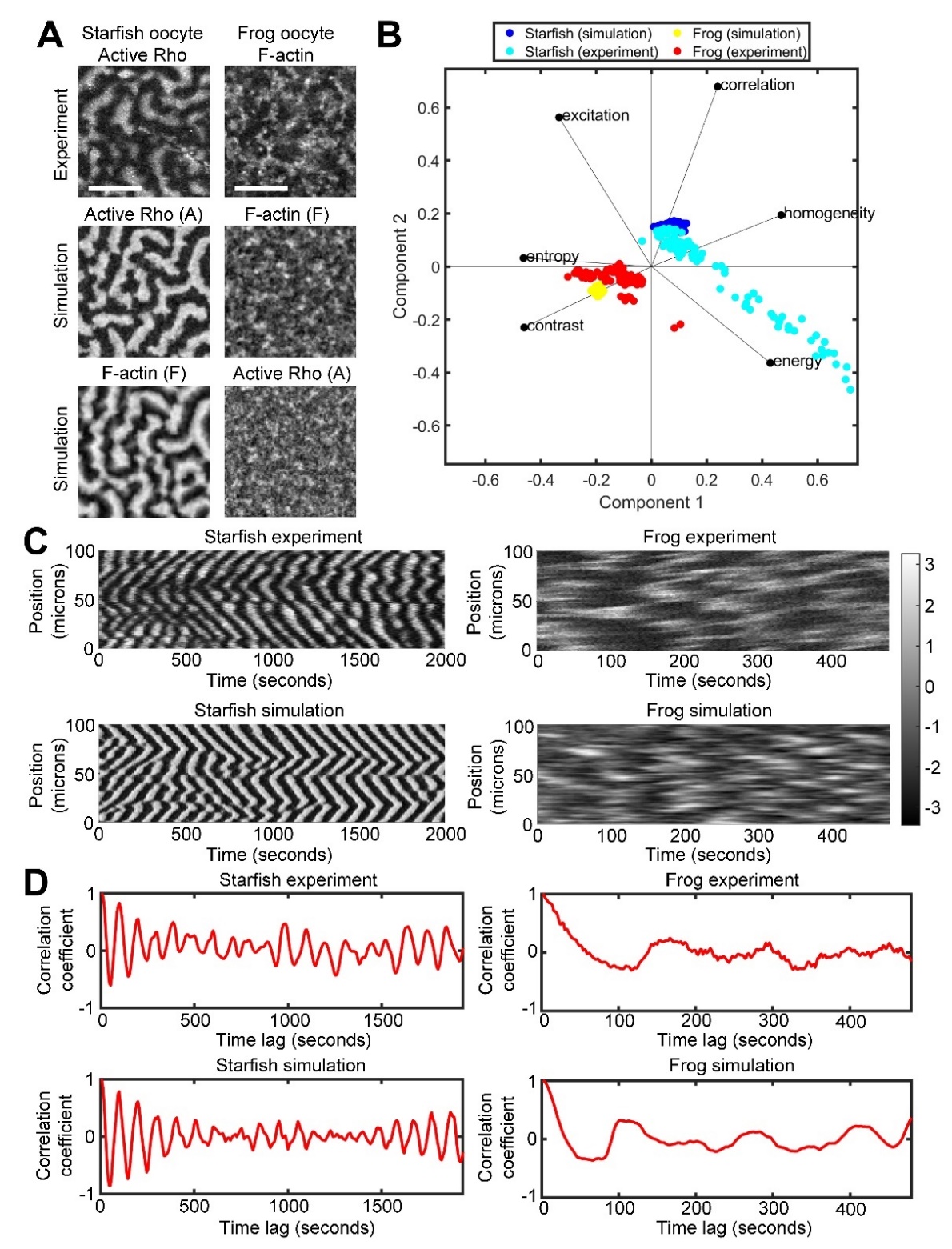


**Supplemental Figure S9.** **Quantitative analysis of active Rho and F-actin patterns in simulations and experimental data (frog and starfish oocyte data from Bement *et al.* [31]).** This figure is similar to **Supplemental Figure S8**, but since Rho/F-actin dynamics in frog oocytes and blastomeres is not identical, we performed the analysis separately for oocytes and blastomeres. **A.** An example of starfish (active Rho) and frog (F-actin) cortical dynamics (upper panels) with the corresponding simulation results (central and lower panels) representing both active Rho and F-actin signals. The scale bar is 40 microns. Here the images are generated from the data published in Bement *et al.* [31] with the permission of the corresponding author. The presented regions of the cell from the experiment and the simulation domain have the size of 100x100 microns. . **B.** Biplot representing the results of PCA analysis of simulations and experimental data. Active Rho signal was used for the starfish data and F-actin for the frog data. 100 consecutive frames were used from each dataset for the analysis. **C.** Representative kymographs from the experimental data and the simulations. The patterns are scaled to have zero mean and standard deviation equal to one. **D.** Temporal autocorrelation plots for the experimental data and the simulations.


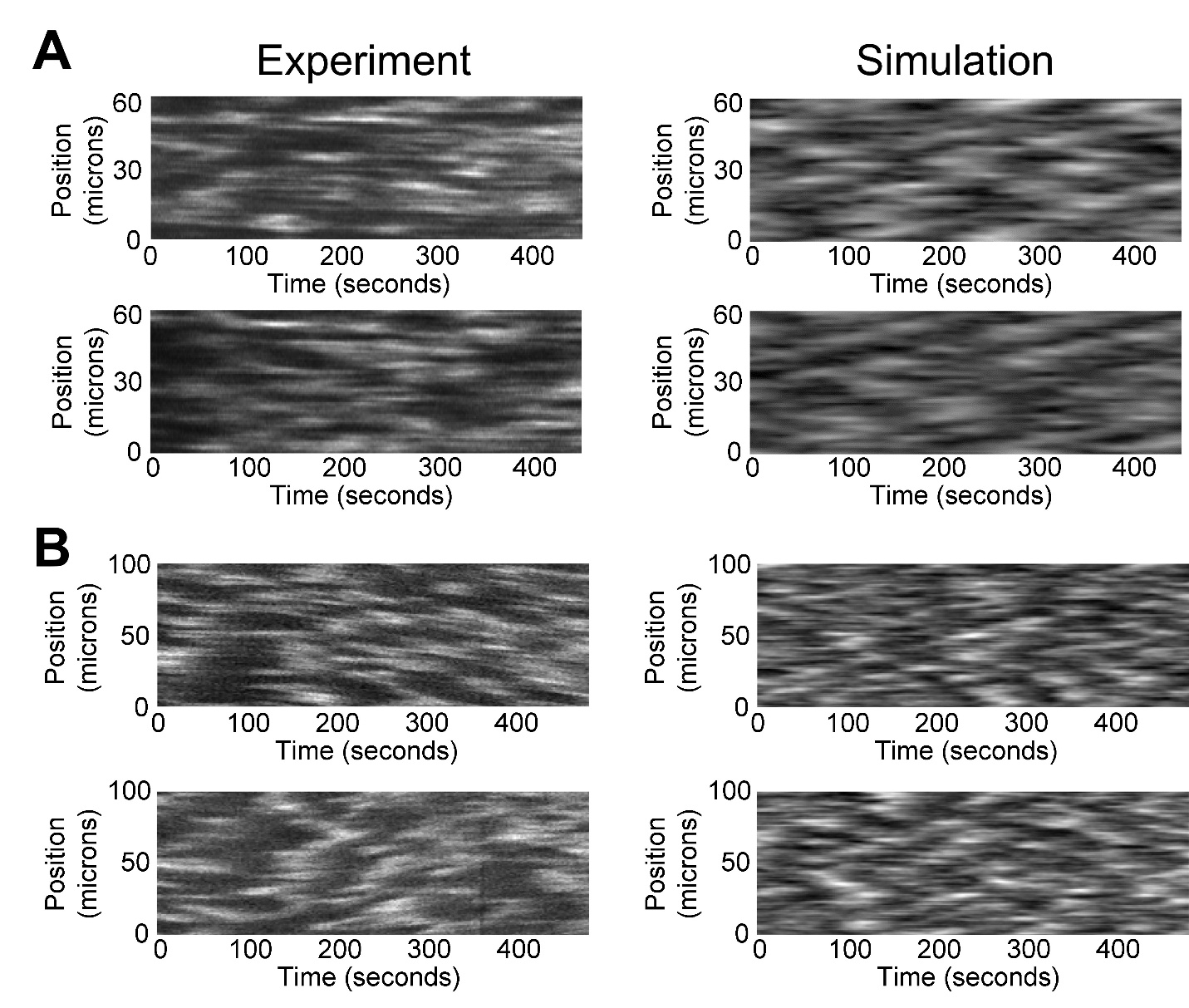


**Supplemental Figure S10.** Additional kymographs using different linear slices in the spatial domain to illustrate experimentally observed (frog oocytes and blastomeres) and simulated dynamics. **A.** F-actin dynamics in the cortex of untreated frog blastomeres during the embryo development. The simulation was performed with parameters $k_{0}=0.25$ and $s_{2}=1.20$. **B.** F-actin dynamics in the cortex of untreated frog oocytes. Experimental images are generated from the data published in Bement *et al.* [31] with the permission of the corresponding author. The simulation was performed with parameters $k_{0}=0.25$*,* $s_{2}=1.25.$


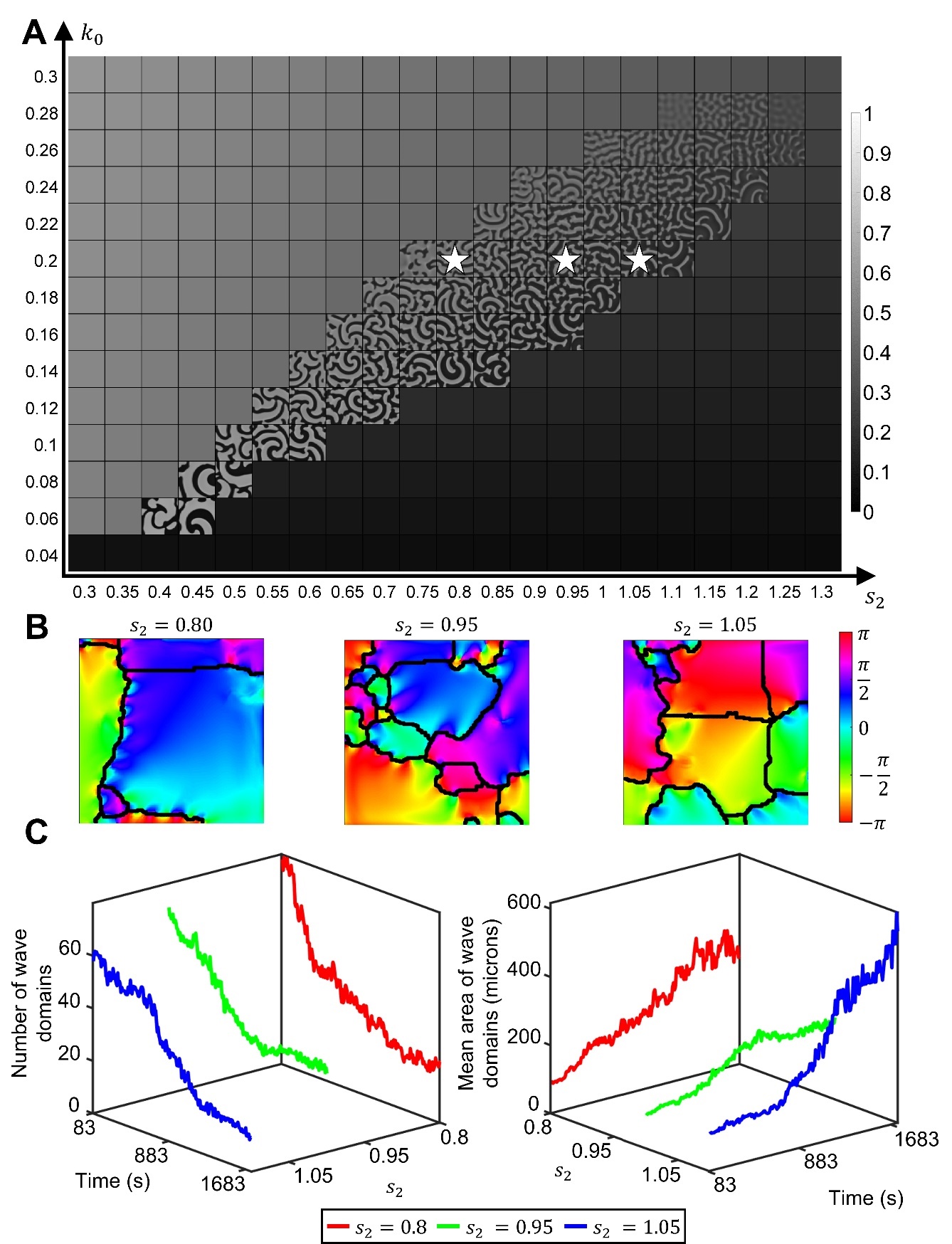


**Supplemental Figure S11.** **A.** Examples of the parameter regimes with different values of negative feedback (marked with white stars), for which the dynamics of wave domains is shown in B and C. **B.** The snapshots of wave domains at 2700 au (2241 seconds) after the start of high-activity stage at the end of six simulations. The duration is 4000 au (3320 seconds). Wave domains are larger in the regimes that are closer to the periphery of the investigated region of the parameter space (i.e., low and high values of the inhibition). In the central part (balanced activation and inhibition), the number of domains is larger, while their mean size is smaller. **C.** The time dependence of the number and the size of wave domains.


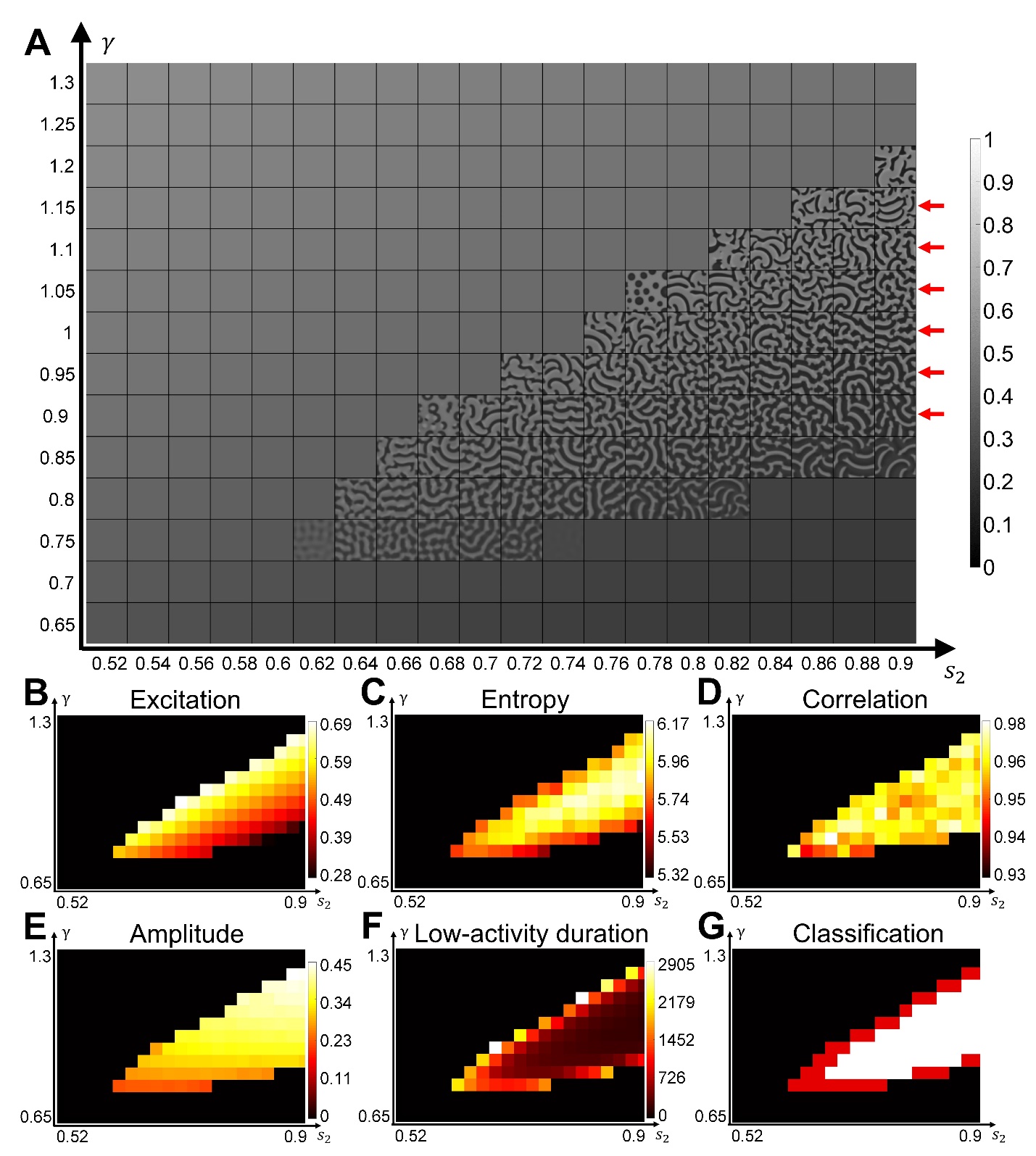


**Supplemental Figure S12. Analysis of the high-activity patterns in a range of parameters representing the positive (**$\gamma$**) and negative (**$s_{2}$**) feedback regulations.** **A.** Patterns resulted in the simulations for a range of parameters $\gamma$ and $s_{2}$ with $k_{0}=0.2$ (see also **Supplemental Video S10**). Red arrows indicate the simulations that are analyzed separately in **Supplemental Figure S13**. **B**. The colormap of the excitation measure (see **Methods**) for the simulations in A. **C, D**. The textural measures of pattern entropy and correlation for the simulations in A. Each measure was averaged over time window 200 au (166 seconds) after the formation of waves from the initial homogeneous state. **E.** The colormap representing the values of activator amplitude during the high activity stage for the simulations in A. **F.** The colormap showing the duration of the low-activity stage (in seconds). **G.** Classification of the patterns with white and red colors indicating the quasi-static and oscillatory regimes, respectively.


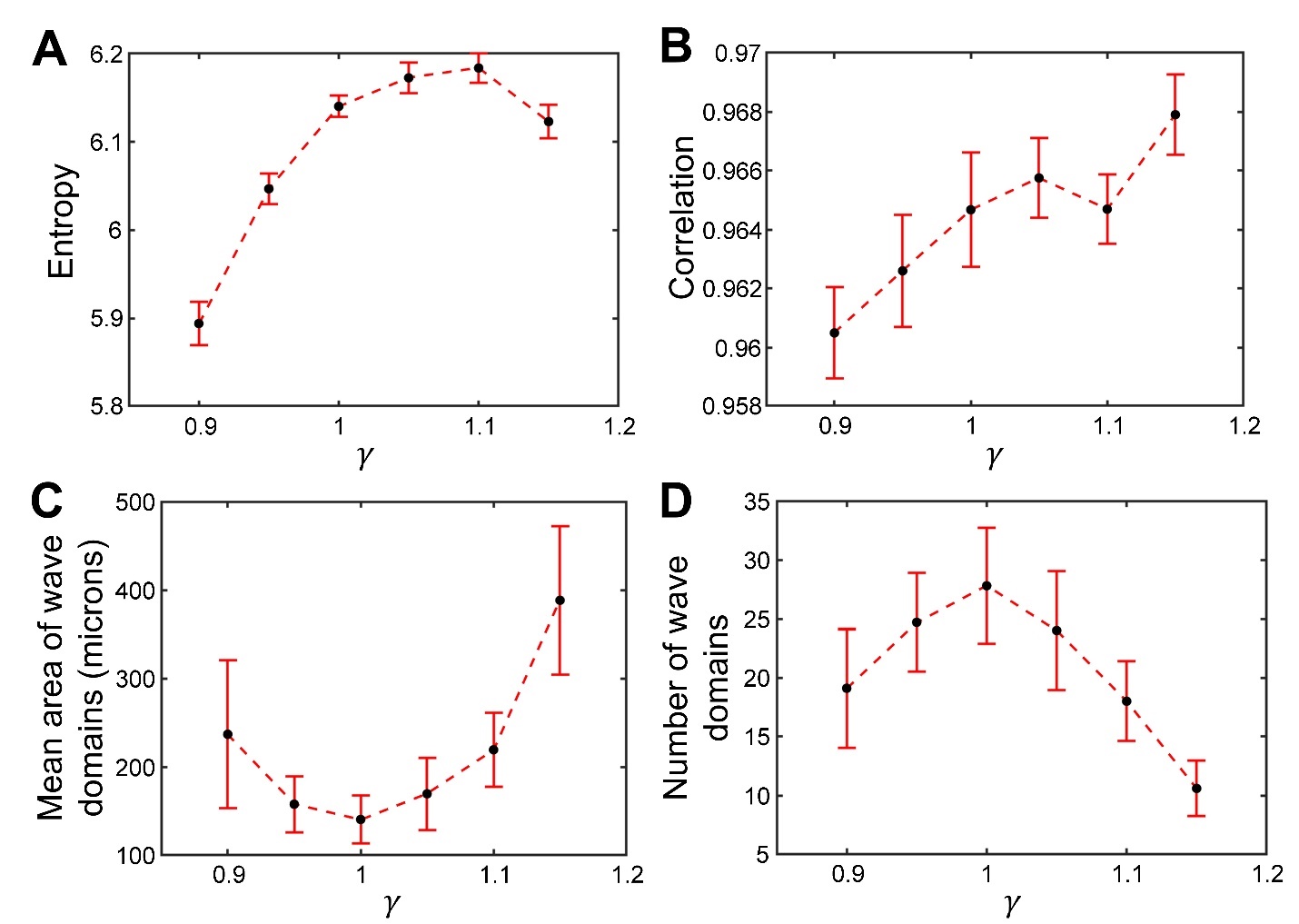


**Supplemental Figure S13.** Statistical analysis of pattern characteristics for the simulations indicated by red arrows in **Supplemental Figure S12**. For each value of $\gamma$ (while $s_{2}$ is fixed to 0.9) we performed 10 independent simulations with randomized initial conditions. **A, B.** Average values of the textural measures (entropy and correlation) at the time equal to 2000 au (1660 seconds) after the beginning of the high-activity stage. Before averaging over repeated simulations, each feature was averaged over the time window of 200 au (166 seconds). **D.** Mean area of wave domains. **E.** The number of wave domains. In all plots, error bars represent 95% confidence interval.


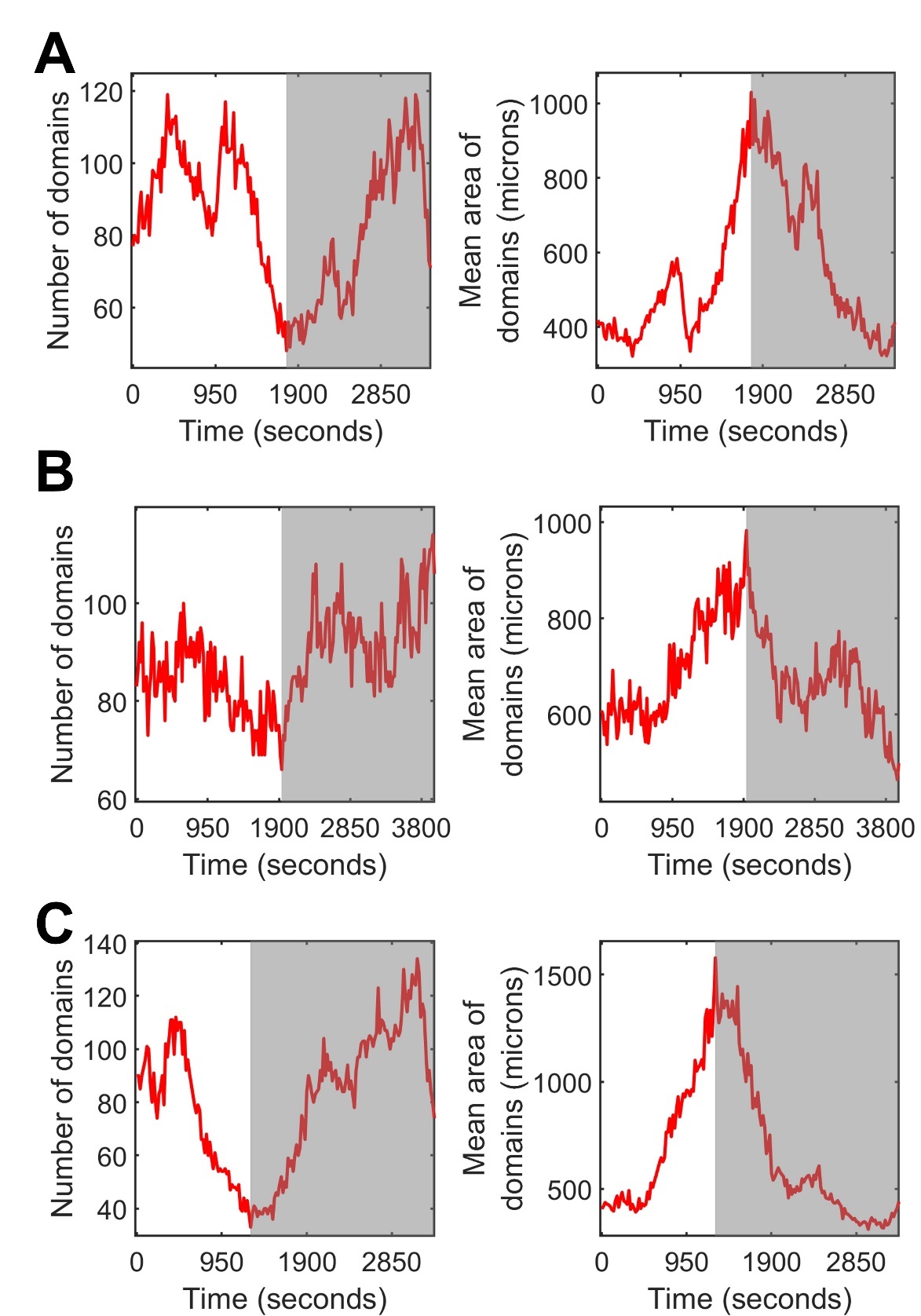


**Supplemental Figure S14.** Wave domains analysis for experimental starfish data from the paper of Bement *et al.* (Supplementary Video 16 in [31]). Here we use the same analysis as in **Fig. 7** but for additional three cells that were fully in the field of view during the whole time-record. In **A, B** and **C** the left plot represents the number of domains, the central plot shows the mean size of domains.


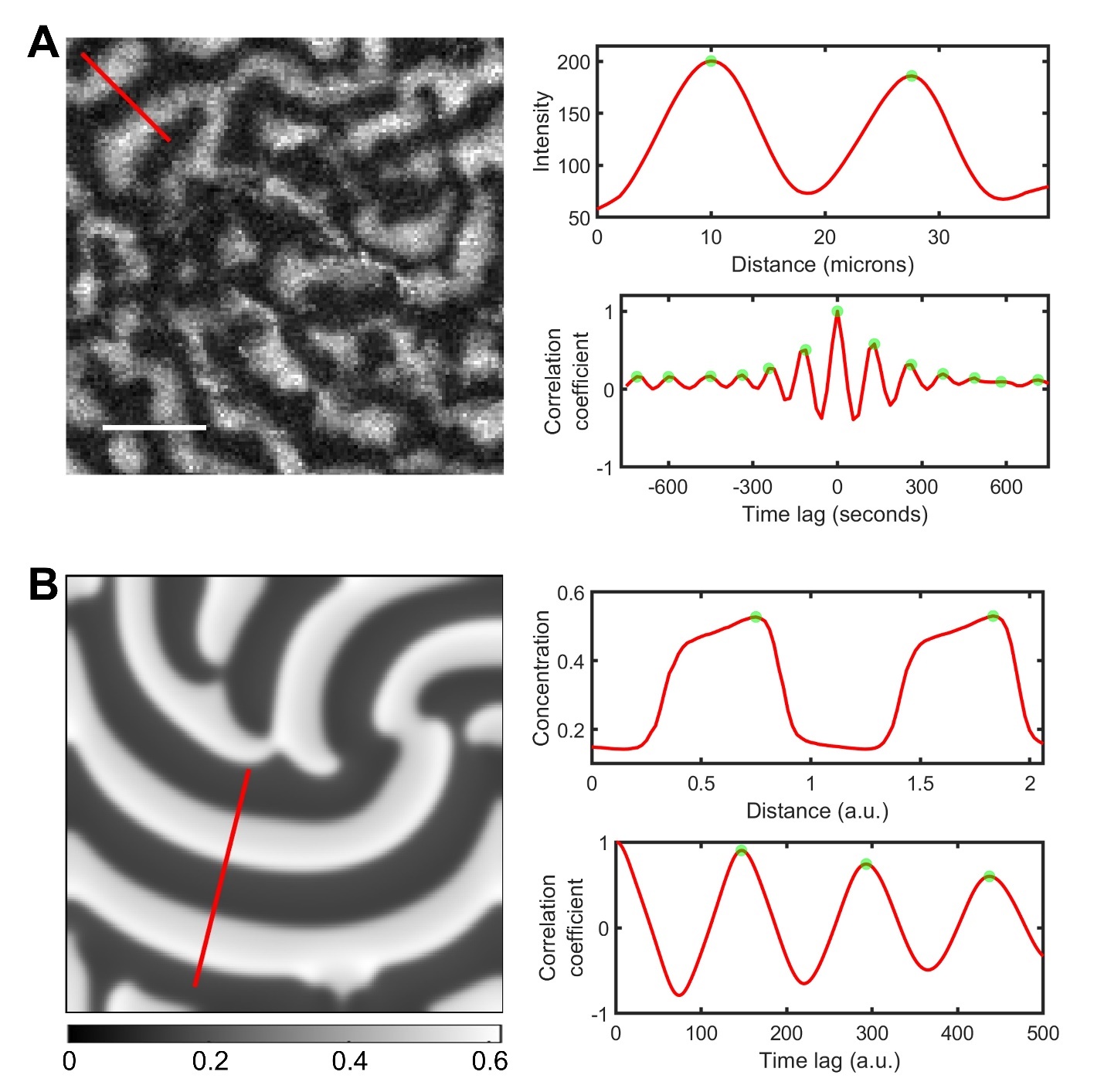


**Supplemental Figure S15.** An estimation of the scaling parameters of the model to relate its arbitrary units with the time (seconds) and space (microns) scales of the experiments. We estimated the temporal period based on the autocorrelation function and spatial period based on image region with a periodic pattern for both the experimental data (**A**) and the simulation (**B**). The green dots are the local maxima used for the measurements. Based on these estimations: $1$ au of time is $\text{0.83}$ seconds, and $1$ au of distance is $15.93$ microns. The scale bar in **A** is 20 microns.


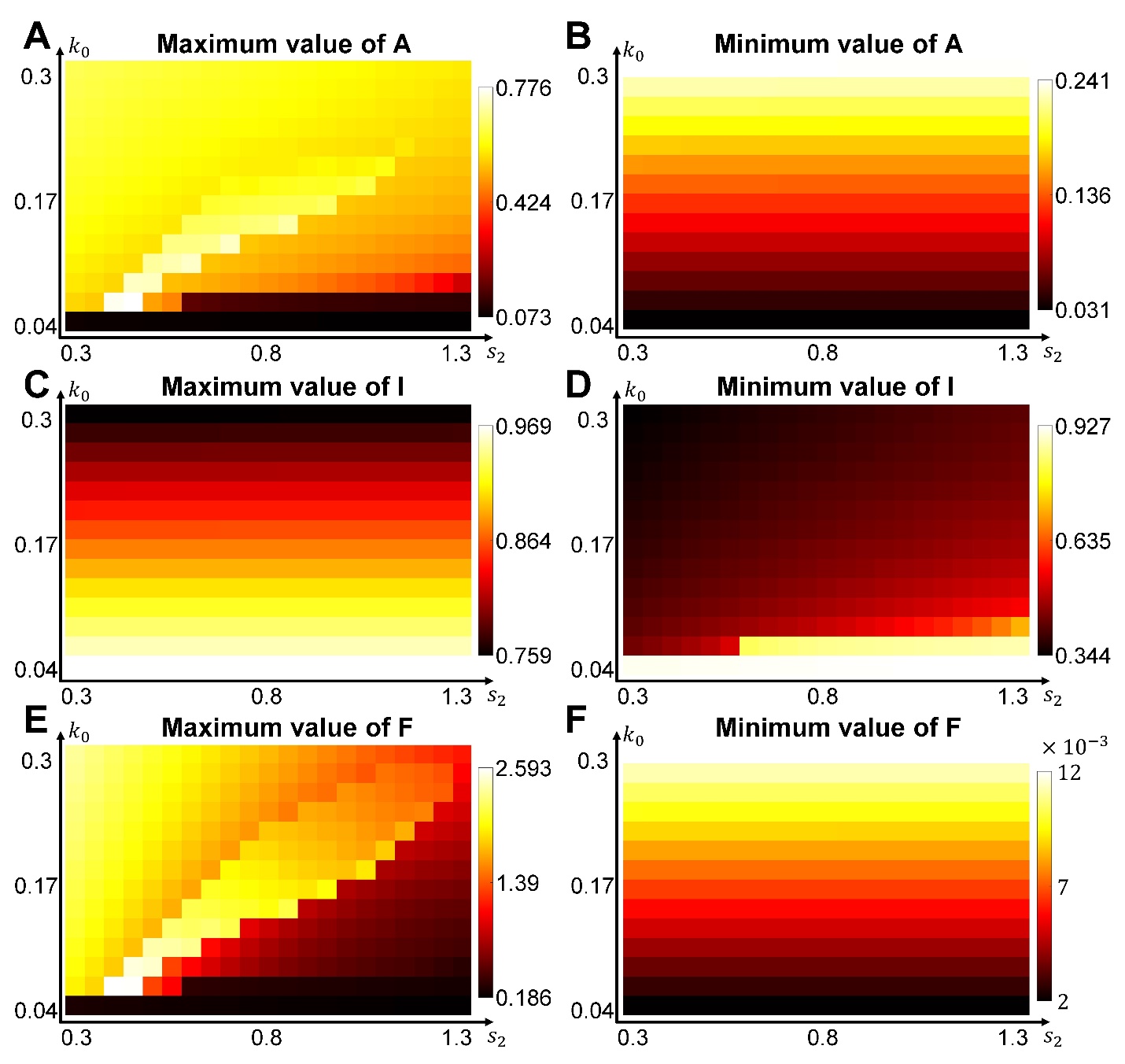


**Supplemental Figure S16.** Maximum and minimum values of the concentrations of the model component: A – active Rho, I – inactive Rho, F – filamentous actin. The ranges of the concentrations were computed for the states of the system saved every 1 au of time (0.83 seconds) for all simulations in the parameter scan with a range of the basal activation and negative feedback values using a small value of noise (see **Fig. 3A** for examples of solutions that we analyze here). Max and min values are taken over the whole simulation domain and over all saved time points. This result shows that in all cases the positivity of our numerical solutions was strictly preserved.


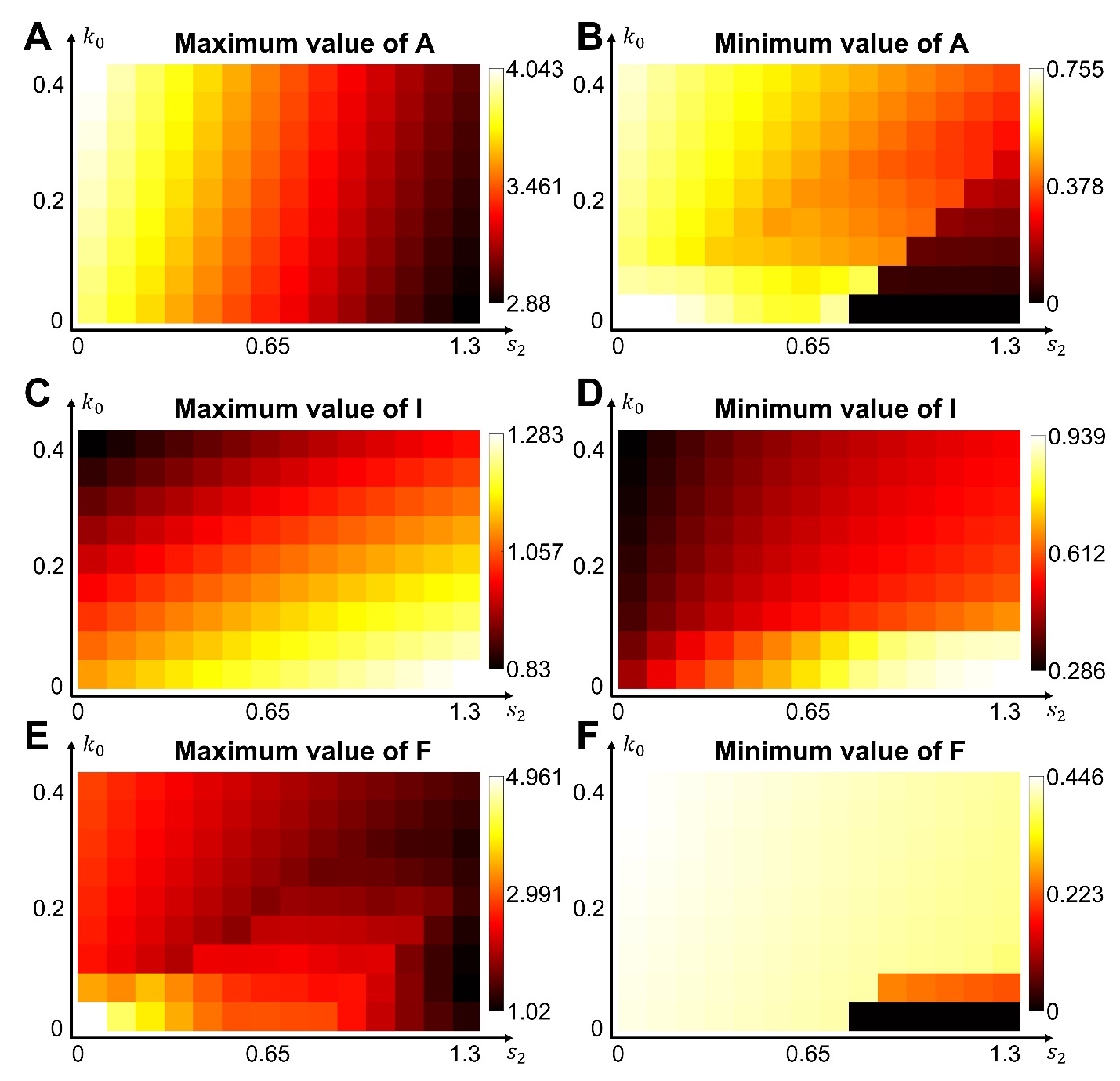


**Supplemental Figure S17.** Maximum and minimum values of the concentrations of the model component: A – active Rho, I – inactive Rho, F – filamentous actin. The ranges of the concentrations were computed for the states of the system saved every 1 au of time (0.83 seconds) for all simulations in the parameter scan with a range of the basal activation and negative feedback values using a spike of activity as initial condition and no noise in the system (see **Supplemental Figure S1** for examples of the patterns in this parameter scan). Max and min values are taken over the whole simulation domain and over all saved time points. This result shows that in all cases the positivity of our numerical solutions was strictly preserved.


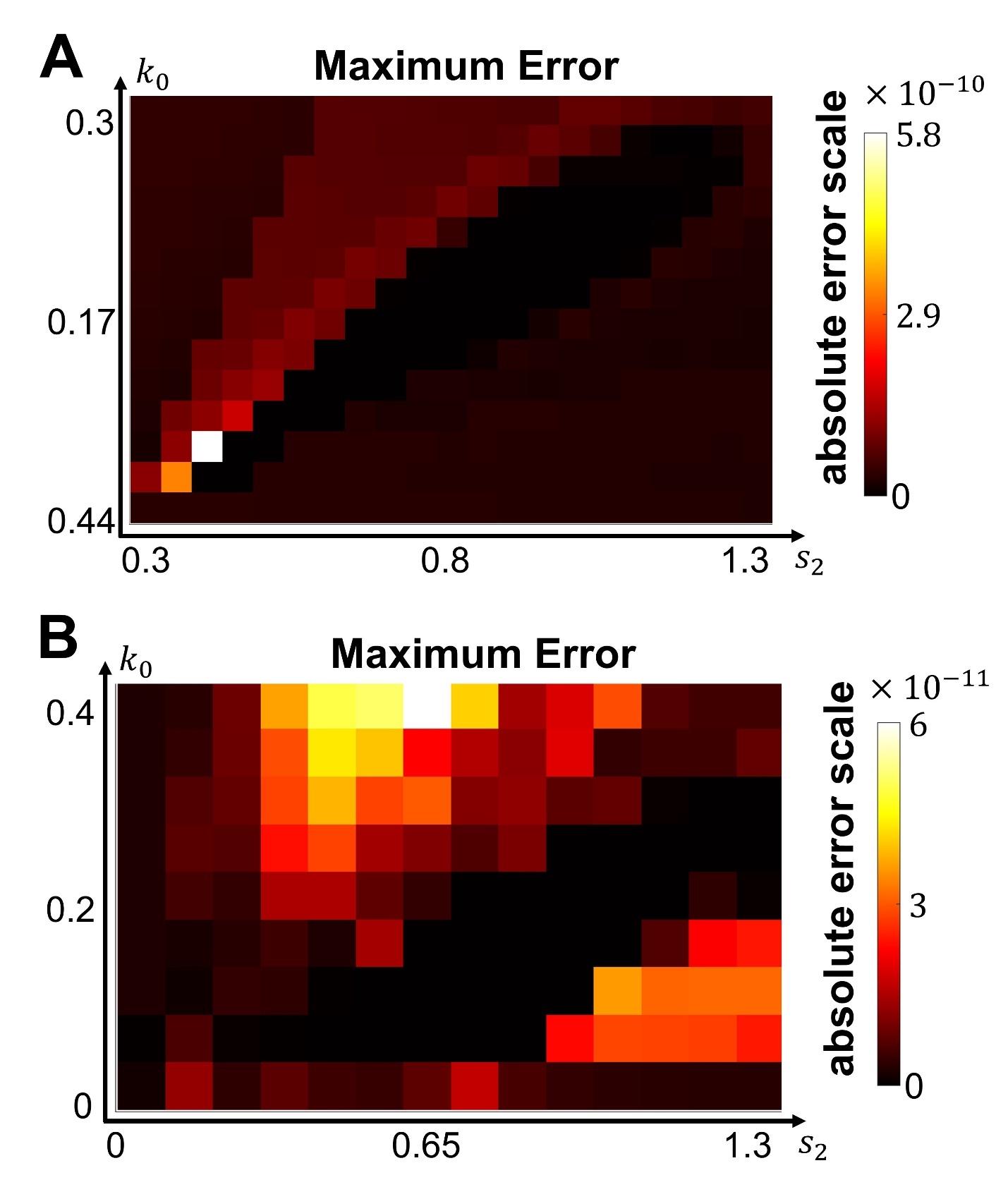


**Supplemental Figure S18.**  Absolute errors (i.e., deviation from the constant value) of the total Rho mass in our simulations with a small noise (**A**) and without noise (**B**). Errors are computed for the states of the system saved every 1 au of time (0.83 seconds) over the whole course of simulation with respect to the initial total Rho mass. The values of absolute errors are between single and double precision of floating-point operation errors. The results show that total mass of Rho in our numerical simulations is conserved with the precision exceeding 10^-11^.


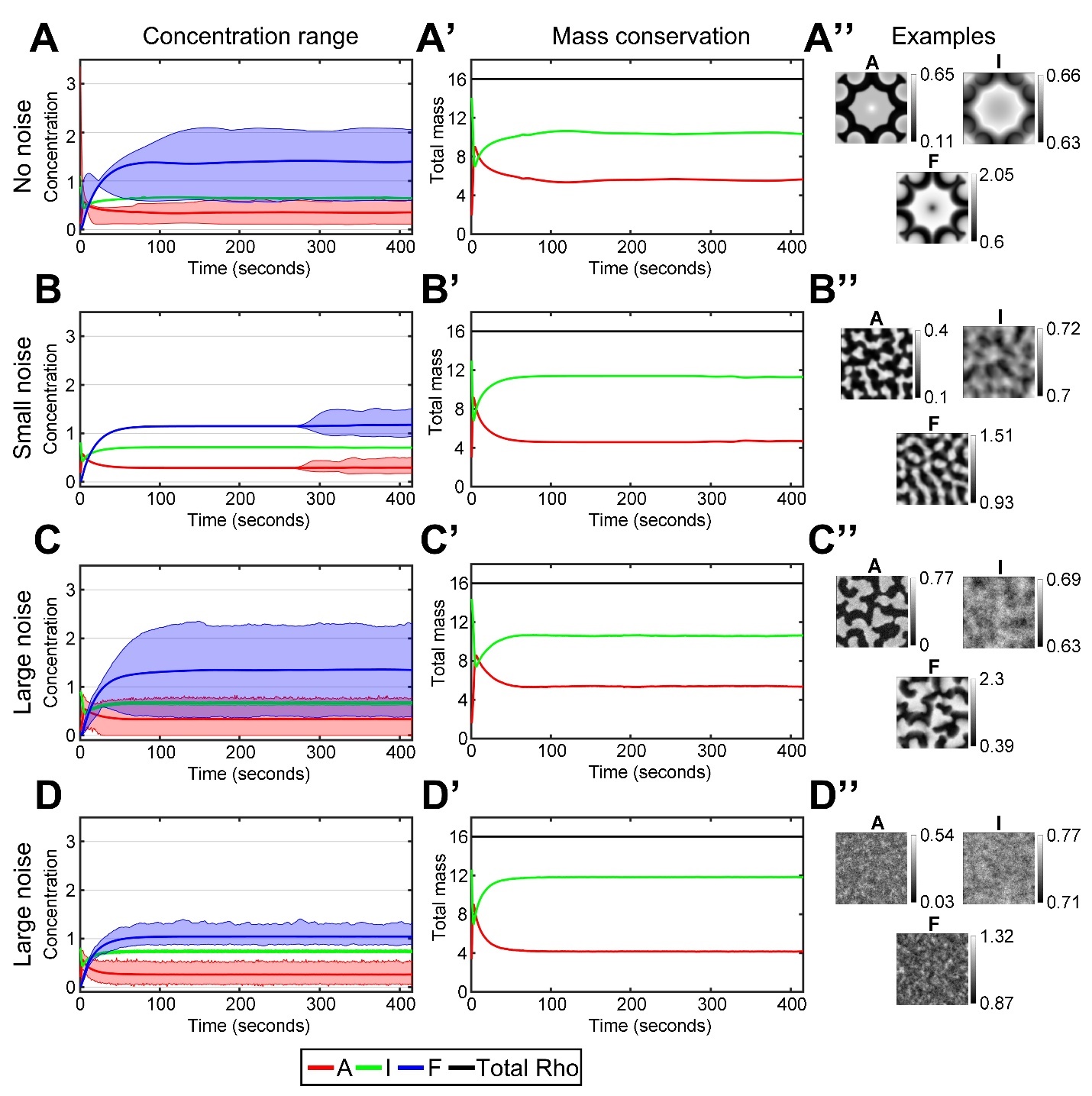


**Supplemental Figure S19.** The ranges of the concentrations of the model components (left panels**)**, the mean concentrations of the active and inactive forms of Rho and the total Rho (central panels), and examples of special patterns (right panels). **A, A’, A’’**. A simulation without noise and with initial excitation in the center of the simulation domain for $k_{0}=0.15, s_{2}=0.7$. **B, B’, B’’**. A simulation with a small value of noise ($\alpha_{1}=\alpha_{2}={10}^{-15})$and the homogeneous initial condition for $k_{0}=0.24, s_{2}=1.1$. **C, C’, C’’.** A Simulation with a large value of noise ($\alpha_{1}=3, \alpha_{2}=0.1)$ and the homogeneous initial condition for $k_{0}=0.1, s_{2}=0.6$, which corresponds to wave behavior of the starfish phenotype. **D, D’, D’’**. A simulation with a large value of noise ($\alpha_{1}=3, \alpha_{2}=0.1$) and the homogeneous initial conditions for $k_{0}=0.25, s_{2}=1.25$, which corresponds to irregular patches of Rho activity of the frog phenotype. These results illustrate that in all cases, regardless of the noise, our solutions do not have negative values of the concentrations (left panels), and the total concentration of Rho is conserved (central panels).


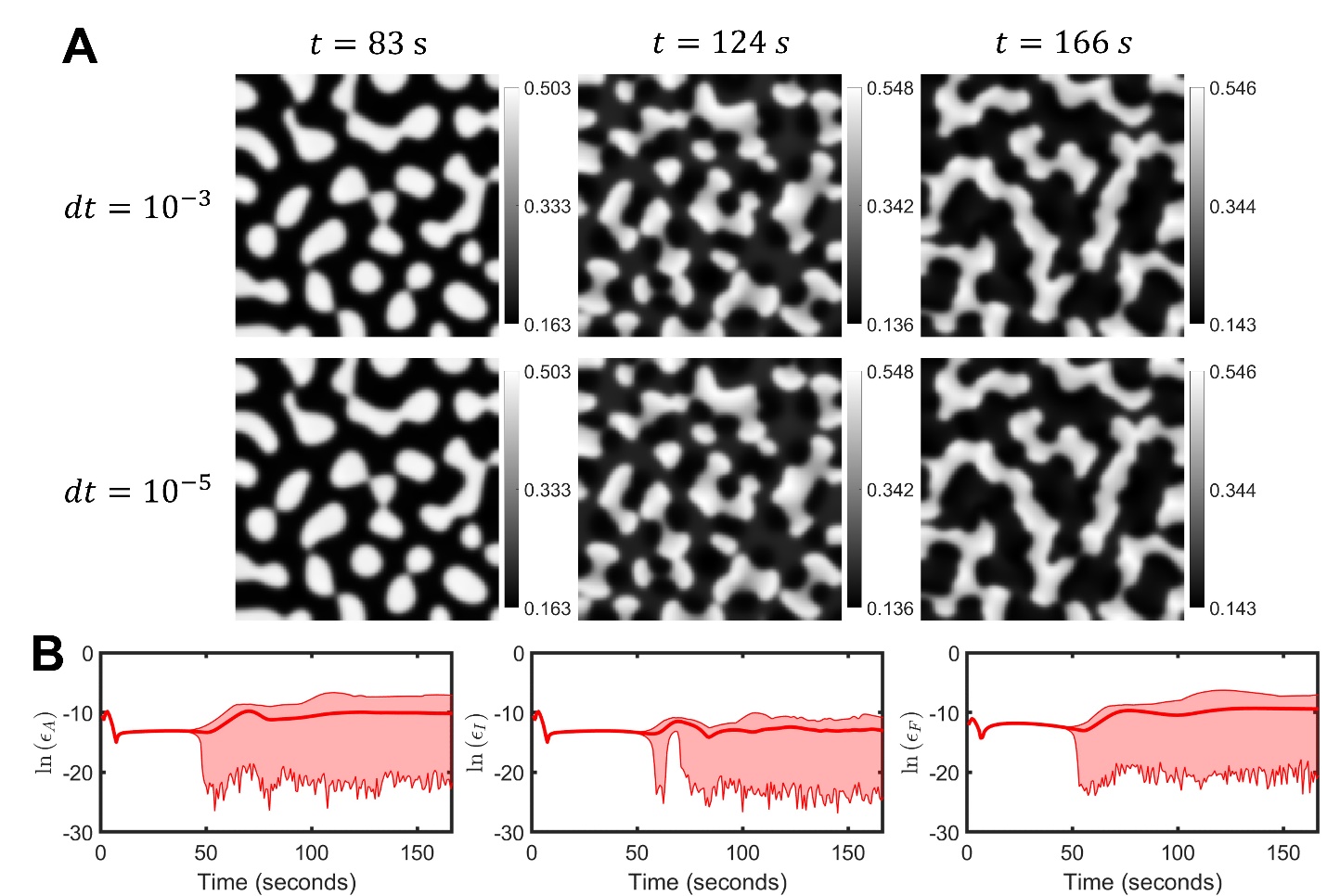


**Supplemental Figure S20.** Comparison of the simulations performed with the forward Euler finite difference scheme using two different time steps. The result shows that our finite difference scheme is numerically stable. Simulations were performed without noise but with identical random perturbation in the initial conditions. **A.** The patterns of the active form of Rho obtained with $t={10}^{-3}$au and $t={10}^{-5}$au are visually identical over the whole time of the simulations. **B.** The statistics of differences in the two solutions over the simulation domain. The red curves show the max, mean, and min of the absolute values of the differences from all grid points as a function of time. The maximal and mean differences taken over time are 0.012 and $2.39\cdot{10}^{-5}$ for the component $A$, $5.3\cdot{10}^{-5}$ and $3.45\cdot{10}^{-6}$ for the component $I$, and 0.0019 and $4.06\cdot{10}^{-5}$ for the component $F$, respectively.


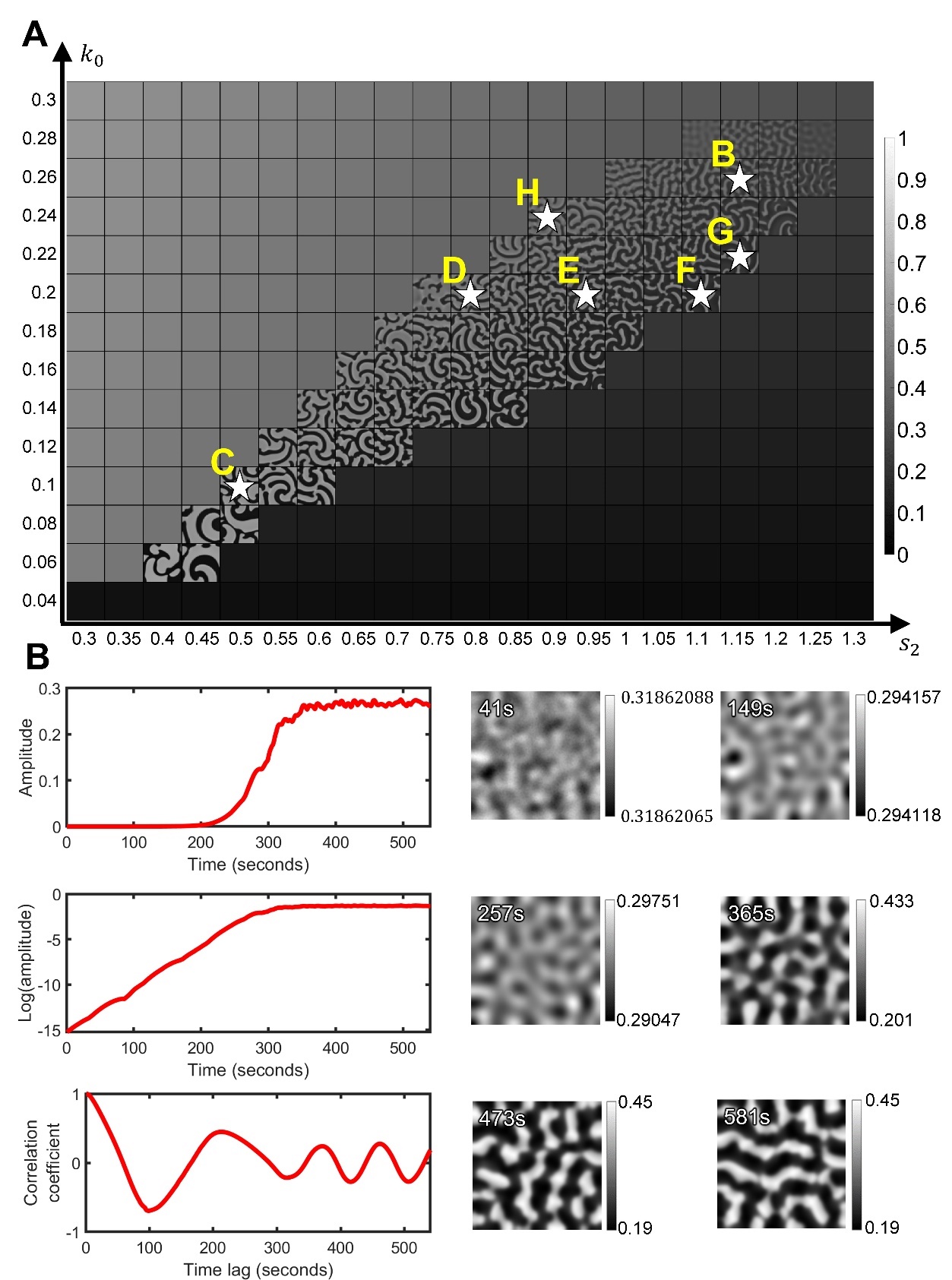


**Supplemental Figure S21.**  Properties of the quasi-static and oscillatory regimes of the low-activity stage were first obtained with the finite difference scheme (see **Fig. 6A**) and were verified with an alternative approach: the finite element method based on the built-in MATLAB implementation. Because MATLAB implementation does not allow to add noise in the system at each iteration, we added small noise only to the initial homogeneous state. **A.** Star symbols indicate parameter regimes, for which we performed simulations with the finite element method. **B** The amplitude of the pattern, logarithm of the amplitude, autocorrelation function, and examples of the solution for active Rho at different time points of the simulation in the parameter regime B ($k_{0}=0.26$, $s_{2}=1.25$). See **Supplemental Figures S22-S24** for the regimes C to H. In all cases, the quasi-static and oscillatory behaviors of the system in the low-activity stage were the same as obtained with the finite difference method.


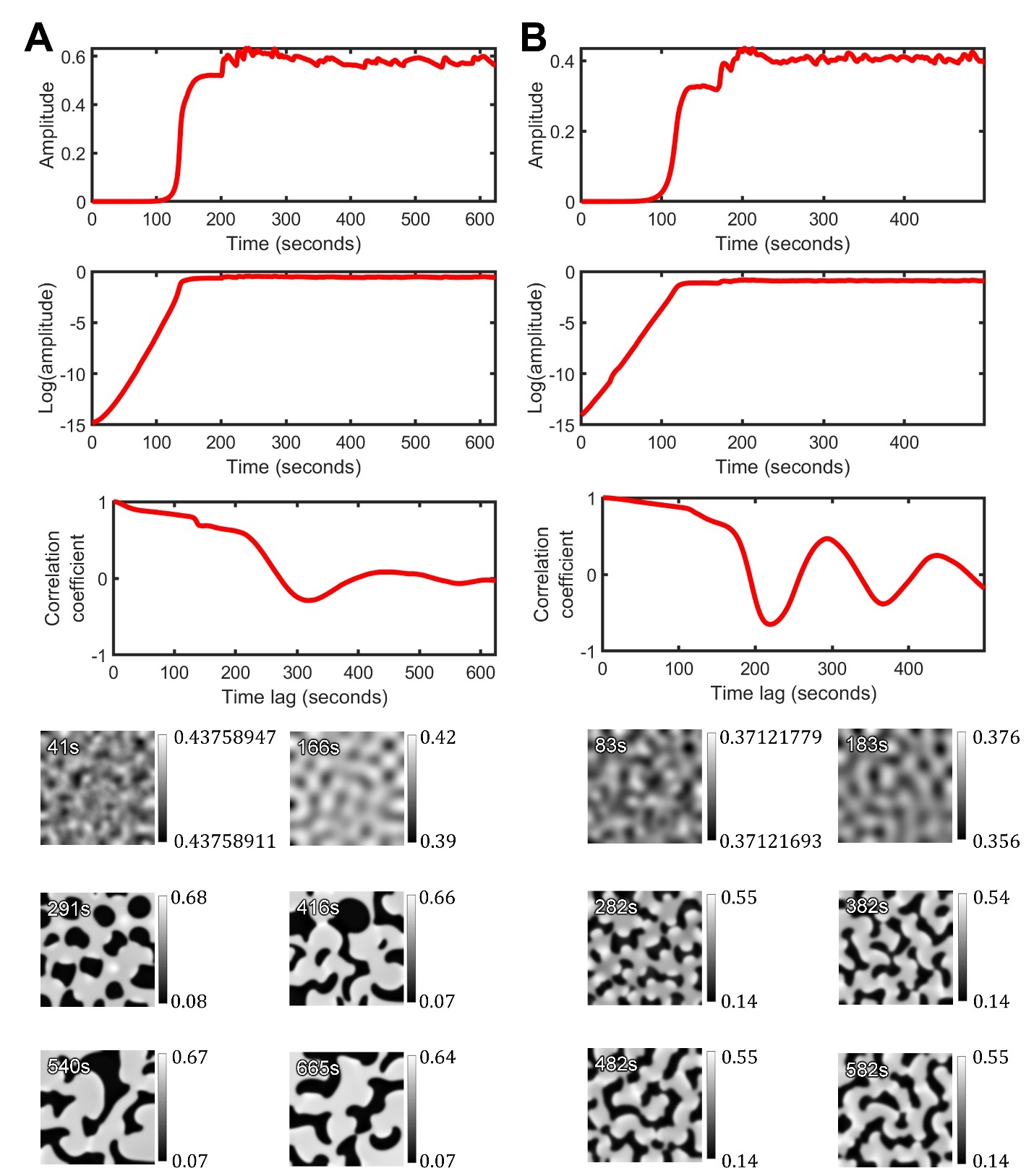


**Supplemental Figure S22. A, B** The amplitude of the pattern, logarithm of the amplitude, autocorrelation function, and examples of the solution for active Rho at different time points of the simulation in the parameter regimes C ($k_{0}=0.1$, $s_{2}=0.5$) and D ($k_{0}=0.2$, $s_{2}=0.8$) of the parameter space (see **Supplemental Figure S21A**), respectively.


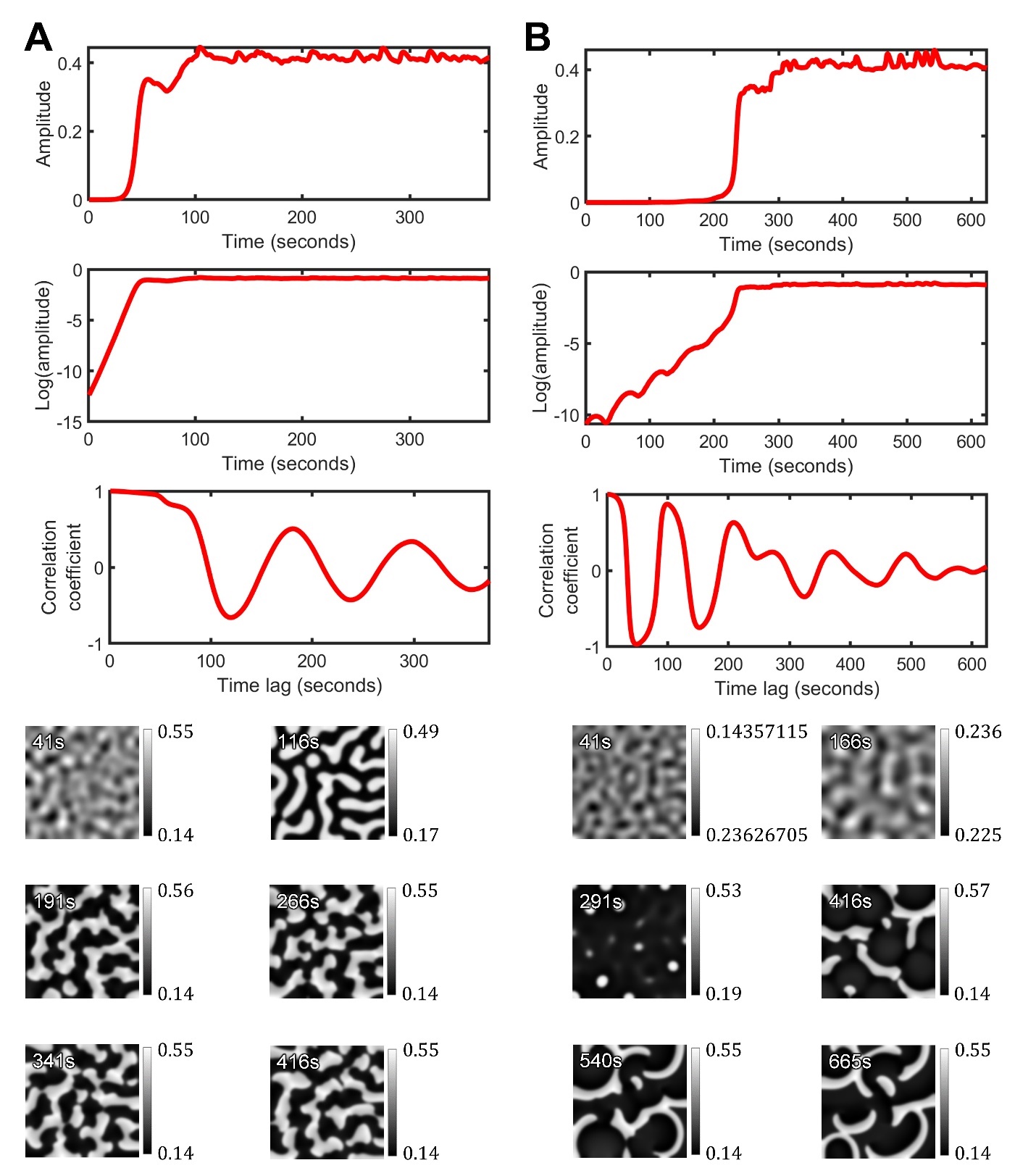


**Supplemental Figure S23. A, B** The amplitude of the pattern, logarithm of the amplitude, autocorrelation function, and examples of the solution for active Rho at different time points of the simulation in the parameter regimes E ($k_{0}=0.2$, $s_{2}=0.95$) and F ($k_{0}=0.2$, $s_{2}=1.1$) of the parameter space (see **Supplemental Figure S21A**), respectively.


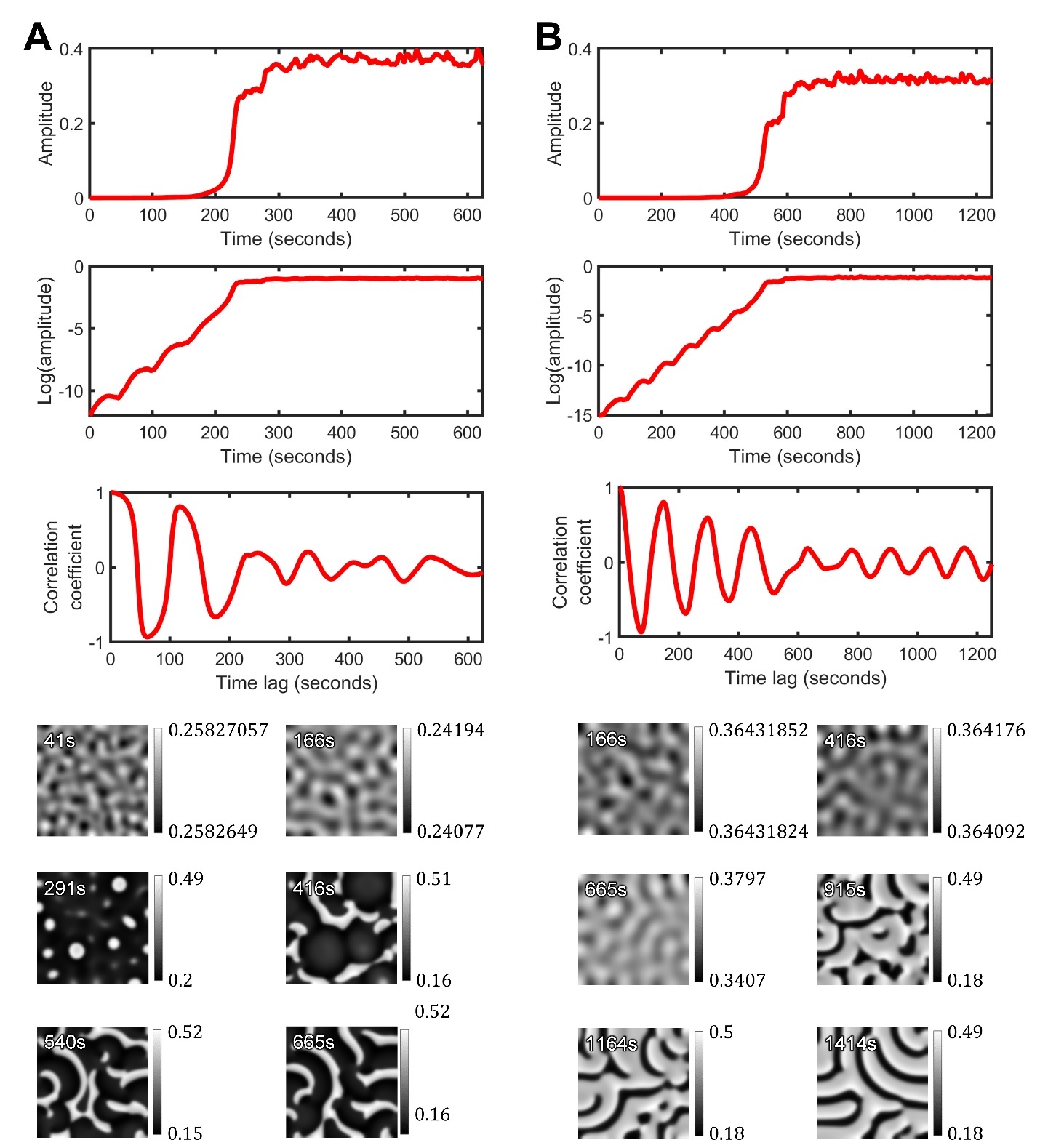


**Supplemental Figure S24. A, B** The amplitude of the pattern, logarithm of the amplitude, autocorrelation function, and examples of the solution for active Rho at different time points of the simulation in the parameter regimes G ($k_{0}=0.22$, $s_{2}=1.15$) and H ($k_{0}=0.24$, $s_{2}=0.9$) of the parameter space (see **Supplemental Figure S21A**), respectively.


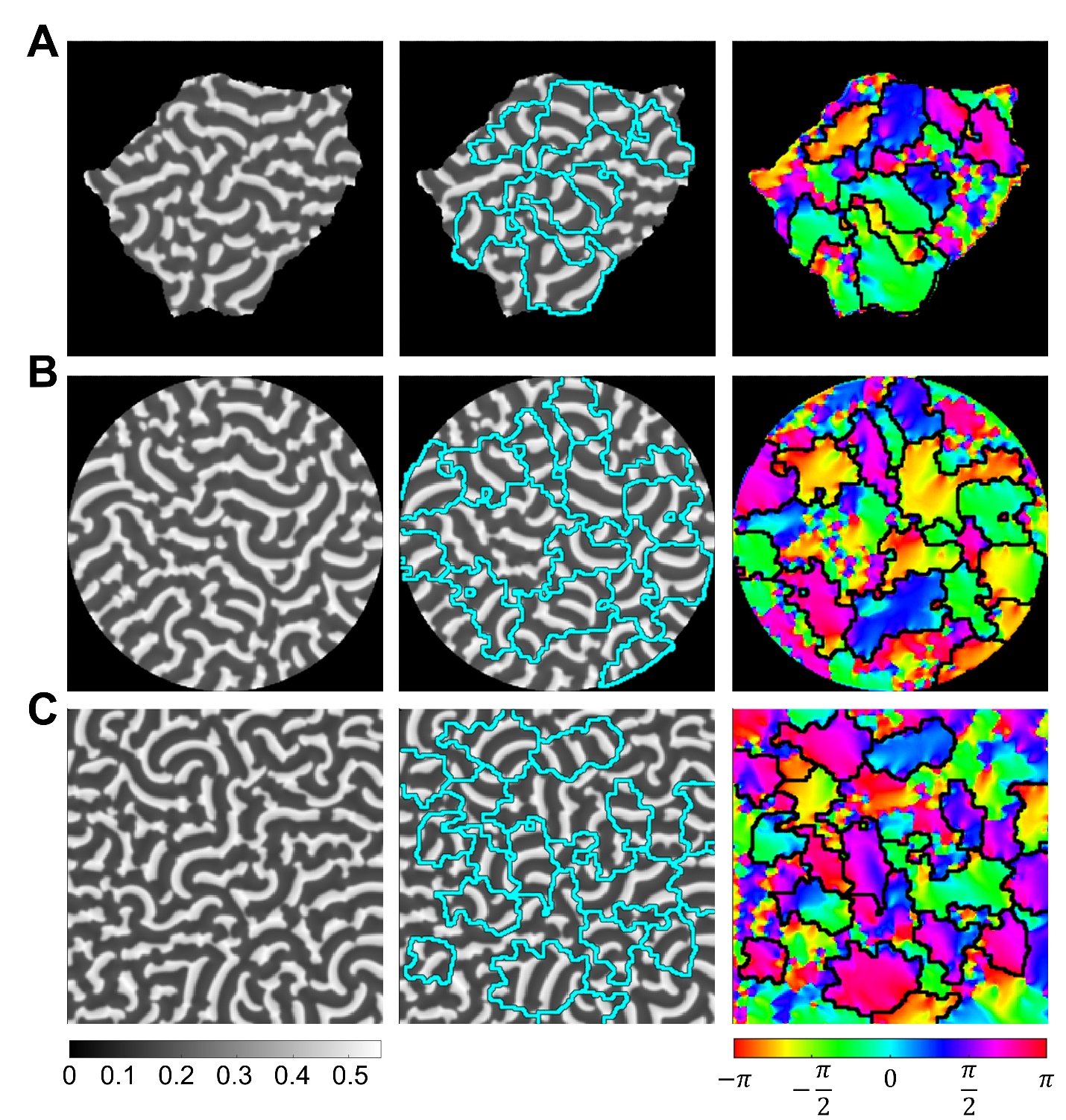


**Supplemental Figure S25.** Examples of wave-domains formation in the simulation domains of different shapes: irregular (**A**), circular (**B**), and square (**C**). In all cases, wave-domains are formed across the whole simulation domain. These results illustrate that the phenomenon of wave domain formation is not a result of a particular setup of the simulation domain. Parameters that were used for simulation: $k_{0}=0.2$, $s_{2}=1$.


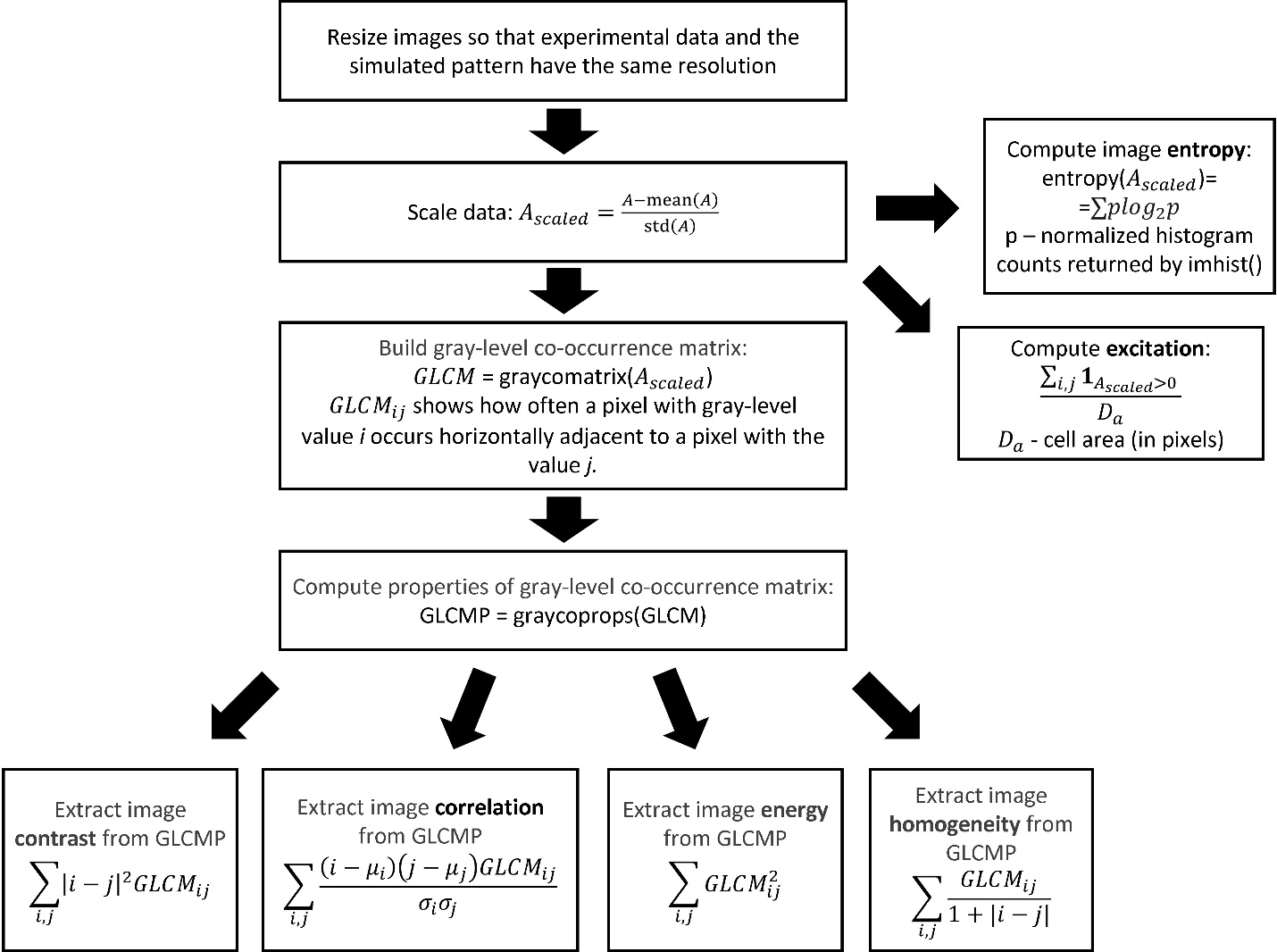


**Supplemental Figure S26.** The pipeline for our textural analysis. Before computing textural features with the built-in MATLAB functions, we resized the images (so that they have identical resolution) and identically scaled ranges of intensity values across the image.


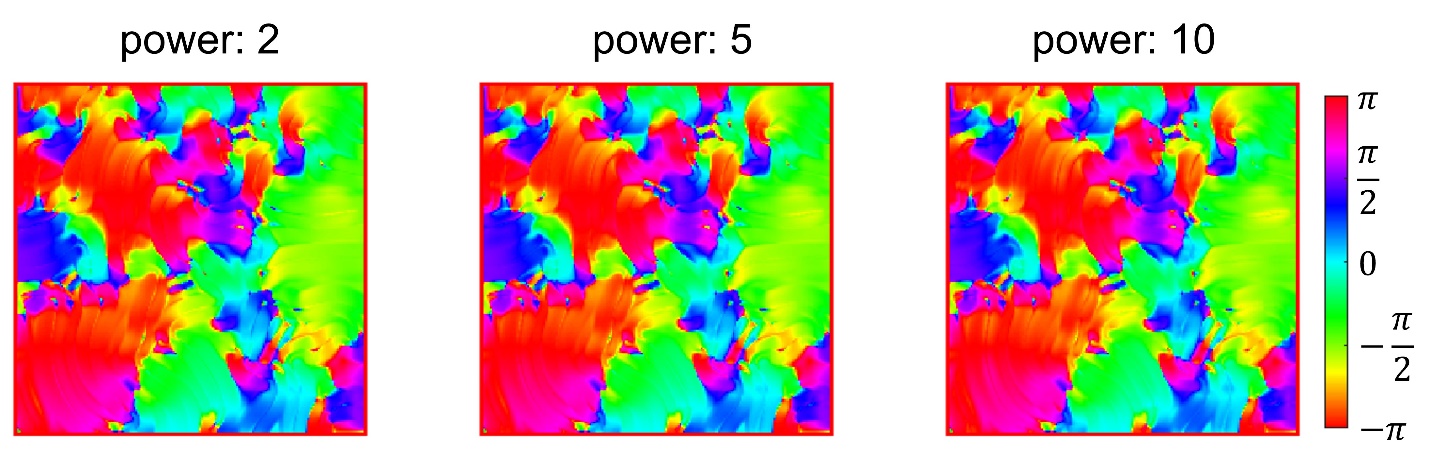


**Supplemental Figure S27.** Examples of the output for the algorithm of wave-direction detection for different values of the power parameters in Equation (7) of the main text. These results illustrate that the algorithm is not sensitive to this parameter.

**Supplemental Text**

**Comparison of Rho/F-actin dynamics in the model with increased noise and experimental data.**

In the parameter regimes of the model where high-activity patterns have relatively small amplitudes (cyan region in **Fig. 6A** and **Supplemental Figure S4A**), sufficiently large noise $\alpha$ disrupts wave propagation. As a result, instead of continuous wave fronts, the system develops irregular flickering of disjoint patches of activation (**Supplemental Figure S6**). If the system is shifted closer to the periphery of the Turing unstable regime by increased inhibition (**Supplemental Figure S7**), the distance between activity patches is increased and the resulting dynamics starts to resemble F-actin behavior in the cortex of frog (*Xenopus laevis*) oocytes in [31] and in blastomeres during the later stage of embryo development in our experiments. In contrast, in the parameter regimes with quasi-static low-activity stage (white region in **Fig. 6A**) the wave patterns are more robust to noise and for high values of $\alpha$ closely reproduce Rho activity in starfish oocytes (**Supplemental Figure S6 and S7**). For both frog-like and starfish-like parameter regimes, the model generates similar dynamic behavior of active Rho ($A$) and F-actin ($F$). However, because it is assumed that $F$ component has zero diffusion, it is easier to perturb this component by noise. Thus, to reproduce correctly both $A$ and $F$ channels, we applied smaller noise for $F$ ($\alpha_{2}=0.1$) then for $A$ ($\alpha_{1}=3$). Also, note that data from frog cells were collected under different experimental conditions than the data from starfish oocytes. While Rho-GEF Ect2 overexpression was required for starfish oocytes to produce waves in the whole cortex of the cells, in frog cells, F-actin and Rho changes are visible on the whole-cell scale under the wild-type conditions. The frog cells were also not perturbed by induced expression of ∆90 cyclin B or by roscovitine.

The comparison of model simulations with the experimental data was performed based on textural features of the resulting pattern (see **Methods** for more details). For starfish oocytes, we analyzed active Rho (because this measurement was reported in the original paper by Bement *et al.* [31]) but presented our simulation results for both $A$ (Rho) and *F* (F-actin). For frog blastomeres, we fitted the model parameters based on the available F-actin data but reported simulation results for both $A$ and $F$ (**Supplemental Figure S8A**). Cell regions of identical size (64x64 microns) were chosen both for starfish and frog cells. Based on the results of our PCA analysis (**Supplemental Figure S8B**) we determined the parameters of the model to be $k_{0}=0.19, s_{2}=0.9$ for starfish and $k_{0}=0.25, s_{2}=1.2$ for frog dynamics. The agreement between the experimental data and our simulations were also illustrated with kymographs (**Supplemental Figure S8C**), temporal autocorrelation plots (**Supplemental Figure S8D**), and side-by-side video (**Supplemental Video S8)**.

F-actin dynamics in frog oocytes is similar but not identical to frog blastomeres. Oocytes have smaller patches of activation and do not resemble parts of disjoint wavefronts as it is in the cortex of blastomeres. Thus, we performed a separate quantitative analysis of the set of experimental data from starfish and frog oocytes in Bement *et al.* [31]. The size of the cell regions in this analysis is larger (100x100 microns) due to the larger size of oocytes in comparison to blastomeres. Based on our PCA analysis of the textural features, the best fit of the model was achieved with slightly increased negative feedback ($s_{2}=1.25$) in the simulations of the frog-like F-actin dynamics (see **Supplemental Figure S9** and **Supplemental Video S9**). To provide a better sense of the F-actin dynamics in oocytes and blastomeres, we plotted additional kymographs using different line scans in the field of view (**Supplemental Figure S10**).

Our analysis shows that the transition between two distinct dynamic behaviors: continuous wavefronts that form wave domains as in treated starfish oocytes and flickering patches of activation as in wild-type frog blastomeres and oocytes, can be achieved through a proper adjustment of both the activation rate of Rho and negative feedback from F-actin.

**Model scaling**

Original equations:

$$\frac{\partial A}{\partial t}=\left( k_{0}+\gamma\frac{A^{3}}{{A_{0}}^{3}+A^{3}} \right)I-\left( s_{1}+s_{2}\frac{F}{F_{0}+F} \right)A+D_{A}\frac{\partial^{2}A}{\partial x^{2}}+D_{A}\frac{\partial^{2}A}{\partial y^{2}}+\alpha_{1}\xi_{1}$$

$$\frac{\partial I}{\partial t}=-\left( k_{0}+\gamma\frac{A^{3}}{{A_{0}}^{3}+A^{3}} \right)I+\left( s_{1}+s_{2}\frac{F}{F_{0}+F} \right)A+D_{I}\frac{\partial^{2}I}{\partial x^{2}}+D_{I}\frac{\partial^{2}I}{\partial y^{2}}-\alpha_{1}\xi_{1}$$

$$\frac{\partial F}{\partial t}=k_{n}A-k_{s}F+\alpha_{2}\xi_{2}$$

Scaling

$$\tilde{x}=rx$$

$$\tilde{y}=ry$$

$$\tilde{t}=\tau t$$

where $r$ – spatial scaling factor for conversion of arbitrary units of distance to microns, $\tau$ – temporal scaling factor for conversion of arbitrary units of time to seconds. Scaling factors were estimated from comparison of experimental data and simulations by matching spatial and temporal periods (see **Supplemental Figure S15**).

Scaled equation:

$$\frac{\partial A}{\partial\tilde{t}}=\left( \tilde{k}_{0}+\tilde{\gamma}\frac{A^{3}}{{A_{0}}^{3}+A^{3}} \right)I-\left( \tilde{s}_{1}+\tilde{s}_{2}\frac{F}{F_{0}+F} \right)A+\tilde{D}_{A}\frac{\partial^{2}A}{\partial\tilde{x}^{2}}+\tilde{D}_{A}\frac{\partial^{2}A}{\partial\tilde{y}^{2}}+\tilde{\alpha}_{1}\xi_{1}$$

$$\frac{\partial I}{\partial\tilde{t}}=-\left( \tilde{k}_{0}+\tilde{\gamma}\frac{A^{3}}{{A_{0}}^{3}+A^{3}} \right)I+\left( \tilde{s}_{1}+\tilde{s_{2}}\frac{F}{F_{0}+F} \right)A+\tilde{D}_{I}\frac{\partial^{2}I}{\partial\tilde{x}^{2}}+\tilde{D}_{I}\frac{\partial^{2}I}{\partial\tilde{y}^{2}}-\tilde{\alpha}_{1}\xi_{1}$$

$$\frac{\partial F}{\partial\tilde{t}}=\tilde{k}_{n}A-\tilde{k}_{s}F+\tilde{\alpha}_{2}\xi_{2}$$

Scaled values of parameters:

$$\tilde{k}_{0}=\frac{k_{0}}{\tau}=\left[ 0,0.3 \right] \left( \frac{1}{s} \right)$$

$$\tilde{\gamma}=\frac{\gamma}{\tau}=1 \left( \frac{1}{s} \right)$$

$$\tilde{s}_{1}=\frac{s_{1}}{\tau}=0.5 \left( \frac{1}{s} \right)$$

$$\tilde{s}_{2}=\frac{s_{2}}{\tau}=[0,1.3] \left( \frac{1}{s} \right)$$

$$\tilde{D}_{A}=\frac{D_{A}r^{2}}{\tau}=\frac{0.001}{3} \left( \frac{{\mu m}^{2}}{s} \right)$$

$$\tilde{D}_{I}=\frac{D_{I}r^{2}}{\tau}=\frac{0.1}{3} \left( \frac{{\mu m}^{2}}{s} \right)$$

$$\tilde{k}_{n}=\frac{k_{n}}{\tau}=0.1 \left( \frac{1}{s} \right)$$

$$\tilde{k}_{s}=\frac{k_{s}}{\tau}=0.025 \left( \frac{1}{s} \right)$$

In all our simulations the total mean concentration of Rho $G_{T}=\frac{A_{total}^{t}+I_{total}^{t}}{D_{a}}$, where $A_{total}^{t}=\sum_{ij} A_{ij}^{t}$, $I_{total}^{t}=\sum_{ij} I_{ij}^{t}$, $D_{a}$ is the total cell area (in number of grid points), and the total mass of Rho, $A_{total}^{t}+I_{total}^{t}=const$, is invariant. So, concentrations of all components are measured in units of total Rho concentration $G_{T}$:

$$A=\left[ 0, 1 \right] G_{T} \left( \frac{mol}{L} \right)$$

$$I=\left[ 0, 1 \right] G_{T} \left( \frac{mol}{L} \right)$$

$$F=\left[ 0, 3 \right] G_{T} \left( \frac{mol}{L} \right)$$

Similarly the values of $A_{0}$, $F_{0}$, and noise magnitudes are measured in the same units:

$$A_{0}=0.4 G_{T} \left( \frac{mol}{L} \right)$$

$$F_{0}=0.5G_{T} \left( \frac{mol}{L} \right)$$

$$\tilde{\alpha}_{1,2}=\frac{\alpha_{1,2}}{\tau}=[{10}^{-15},3] \left( \frac{mol}{L s} \right)$$

**Supplemental Video Captions**

**Supplemental Video S1**. Spatiotemporal distribution of Rho activity in the starfish oocyte experiment (left, oocytes have overexpressed Rho-GEF Ect2, induced expression of ∆90 cyclin B, and are treated with roscovitine) and simulation model (right). The images for the left panel are generated from the data published in Bement *et al.* [31] with the permission of the authors. Parameters values in simulation: $k_{0}=0.19$, $s_{2}=0.9$, $\alpha_{1}=3$, $\alpha_{2}=0.1$. The cell mask that was used in the simulation was extracted from experimental data.

**Supplemental Video S2**. The result of a series of simulations representing active Rho dynamics for different values of parameters $k_{0}$ (vertical axes) and $s_{2}$ (horizontal axis) with nonhomogeneous initial conditions (a spike of activity in the center) and without noise.

**Supplemental Video S3**. The results of a series of simulations representing active Rho dynamics for different values of parameters $k_{0}$ (vertical axes) and $s_{2}$ (horizontal axis) with homogeneous initial conditions and small noise $\alpha_{1,2}={10}^{-15}$.

**Supplemental Video S4**. A simulation of wave formation from homogeneous initial conditions in the quasi-static regime of the low-activity stage for a weak inhibition ($k_{0}=0.15$, $s_{2}=0.6$, $\alpha_{1,2}={10}^{-15}$).

**Supplemental Video S5**. A simulation of wave formation from homogeneous initial conditions in the quasi-static regime of the low-activity stage for a balanced (intermediate) inhibition ($k_{0}=0.2$, $s_{2}=0.9$, $\alpha_{1,2}={10}^{-15}$).

**Supplemental Video S6**. A simulation of wave formation from homogeneous initial conditions in the oscillatory regime of the low-activity stage for a strong inhibition ($k_{0}=0.2$, $s_{2}=1$, $\alpha_{1,2}={10}^{-15}$).

**Supplemental Video S7.** The result of a series of simulations representing active Rho dynamics for different values of parameters $k_{0}$ (vertical axes) and $s_{2}$ (horizontal axis) with high value of noise ($\alpha_{1}=3$, $\alpha_{2}=0.1$).

**Supplemental Video S8**. Spatiotemporal distribution of F-actin in the frog blastomeres in our experiment (left, wild type) and our simulation model (right) in the part of cell cortex of the size 64x64 microns. Parameters values in simulation: $k_{0}=0.25$, $s_{2}=1.2$, $\alpha_{1}=3$, $\alpha_{2}=0.1$.

**Supplemental Video S9**. Spatiotemporal distribution of F-actin in the frog oocyte experiment (left, wild type) and simulation model (right) in the part of cell cortex of the size 100x100 microns. The images for the left panel are generated from the data published in Bement *et al.* [31] with the permission of the authors. Parameters values in simulation: $k_{0}=0.25$, $s_{2}=1.25$, $\alpha_{1}=3$, $\alpha_{2}=0.1$.

**Supplemental Video S10**. The results of a series of simulations representing active Rho dynamics for different values of parameters $\gamma$ (vertical axes) and $s_{2}$ (horizontal axis) with homogeneous initial conditions and small noise $\alpha_{1,2}={10}^{-15}$.
